# Elites in social networks: An axiomatic approach to power balance and Price’s square root law

**DOI:** 10.1371/journal.pone.0205820

**Published:** 2018-10-24

**Authors:** Chen Avin, Zvi Lotker, David Peleg, Yvonne-Anne Pignolet, Itzik Turkel

**Affiliations:** 1 Ben Gurion University of the Negev, Be’er-Sheva, Israel; 2 Bar-Ilan University, Ramat Gan, Israel; 3 The Weizmann Institute of Science, Rehovot, Israel; 4 ABB Corporate Research, Baden, Switzerland; Northwestern University, UNITED STATES

## Abstract

A common two-tier structure for social networks is based on partitioning society into two parts, referred to as the *elite* and the *periphery*, where the “elite” is the relatively small but well-connected and highly influential group of powerful individuals around which the society is centered, and the “periphery” consists of the rest of society. It is observed that the relative sizes of economic and social *elites* in various societies appear to be continually declining. One possible explanation is that this is a *natural* social phenomenon, resembling Price’s “square root” law for the fraction of good scientists in the scientific community. We try to assess the validity of this explanation by studying the elite-periphery structure via introducing a novel axiom-based model for representing and measuring the *influence* between the elite and the periphery. The model is accompanied by a set of axioms that capture the elite’s *dominance*, *robustness* and *density*, as well as a *compactness* property. Relying on the model and the accompanying axioms, we are able to draw a number of insightful conclusions about the elite-periphery structure. In particular, we show that in social networks that respect our axioms, the size of a compact elite is *sublinear* in the network size. This agrees with Price’s principle but appears to contradict the common belief that the elite size tends to a linear fraction of society (recently claimed to be around 1%). We propose a natural method to create partitions with nice properties, based on the key observation that an elite-periphery partition is at what we call a *‘balance point’*, where the elite and the periphery maintain a balance of powers. Our method is based on setting the elite to be the *k* most influential nodes in the network and suggest the balance point as a tool for choosing *k* and therefore the size of the elite. When using nodes degrees to order the nodes, the resulting *k*-rich club at the balance point is the elite of a partition we refer to as the *balanced edge-based partition*. We accompany these findings with an empirical study on 32 real-world social networks, which provides evidence that balanced edge-based partitions which satisfying our axioms commonly exist.

## 1 Introduction

### 1.1 Elites in society

Almost all societies exhibit an (often radically) uneven distribution of power, influence, and wealth among their members (rare exceptions are utopian or totally egalitarian societies). This may be related to a well known and by now widely accepted observation made by the pioneering sociologist Vilfredo Pareto in his book *Mind and Society* [1]: “Every people is governed by an *elite*, by a chosen element of the population”. Indeed, social inequality is particularly notable when comparing the *elite*, namely, the small, powerful and influential group at the center of society, against its typically larger, less organized, and less dominant complement, sometimes referred to as the *masses* or the *periphery*.

The relative size of the elite from the population, is an ancient source of interest in the social sciences. Jean-Jacques Rousseau, an 18^th^-century political philosopher (among other things) who influenced the early French Revolution, stated a recommendation, sometimes known as his “law of elites” [2, 3]. Rousseau claimed that a democratic government should be formed of a number of people equal to the square root of the total number of citizens in the state.

A more recent and controversial observation was made by Derek DeSolla Price, a famous historian of science and known as the father of scientometrics. In his classic book *Little Science, Big Science* [4] Price wrote that “the total number of scientists goes up as the square, more or less, of the number of good ones”, where the good scientists are noted as the *elite group*. This became to be known as “Price’s square root law,” which, more formally, claimed that half of the scientific papers are contributed by the square root of the total number of scientific authors. In Price’s words, half of the papers form a ‘point of *symmetry*’ [4], hereafter referred to as a ‘balance point’. Price’s claim was based on an empirical law for productivity, named “Lotka’s law” [5], which is similar in spirit to other *power law* distributions like Zip’s law and Pareto distribution [6, 7]. Price’s law was indeed controversial to many scientists and raised an heated discussion [8, 9] about its accuracy. Empirical results on scientific contributions seem not to match with the (too) strong and (too) exact statement of Price [9].

In economy, recent reports show that the gap between the richest people and the masses keeps increasing, and that decreasingly fewer people amass more and more wealth [10, 11]. Claims like “The top 10 percent no longer takes in one-third of our income – it now takes half,” made by former president Obama [12] when addressing the issue, are interpreted as implying that the economic and political elites become increasingly more greedy and overbearing. Such claims are often used in order to criticize governments and regulatory financial institutions for neglecting to cope with this disturbing development. The question raised by us is: can society help it, or is this phenomenon an unavoidable by-product of some inherent natural properties of society? We claim that in fact, one can predict the shrinkage of elite size over time (as a fraction of the entire society size) based on the very nature of social elites. In particular, in our model, such shrinkage is the natural result of a combination of two facts: First, *society grows*, and second, *elites are much better connected* than peripheries. Combining these facts implies that the fraction of the total population size comprising dense elites will decrease as the population grows with time. And this is what we call Price’s square root law in networks and, in particular, Price’s law for elites in social networks.

Taking a broader look at Price’s law, it states two fundamental principles. First, it claims that to define the elite group, one should look at a *balance point* between the elite and the rest of the population. Second, it claims that the elite size at this point is *sub-linear*, namely, the fraction of the elite out of the total population tends to zero as the population increases. Price claimed the elite size is about $\sqrt{n}$ for a population of *n* individuals. We study a weaker statement where the elite is *n*^*x*^ of the population (for $\frac{1}{2}\leq x<1$), taking the stand that the important property is the *sub-linearity* of the elite and not the exact exponent *x*. As opposed to Price and subsequent studies, we do not rely our statements on a particular family of exact distributions of individuals influence (or productivity) like power law distributions, but take an *axiomatic* approach to the study of elites.

The division between elite and periphery in social networks can be thought of as an instance of a more general phenomenon, referred to as a *core-periphery* partition of the network [13], which is exhibited by most complex networks. The resulting core-periphery structure may be rightfully considered the most visible high-level structure of society. Consequently, considerable attention was recently given to the questions of identifying this partition and studying its basic properties [14–16]. The common features of the core nodes are high connectivity and higher centrality values than the periphery nodes. These properties are exhibited also by elites in social networks. At the same time, social elites have some additional properties of interest, which set them apart and justifies their independent study. In particular, two notable characteristics of elites are that they are relatively *small* and that they possess a *disproportionate* fraction of the power, resources, and influence in society.

### 1.2 Axioms of elite-centered networks

This paper concentrates on studying the properties of social elites. Its main contribution is a characterization of elites, given in the form of a set of properties (stated as axioms) concerning influence (on itself and on the periphery) that any elite must possess. Our axioms attempt to capture the key ingredients occuring in elite definitions like the one from Wikipedia:

*“In political and sociological theory, an elite is a small group of people who control a disproportionate amount of wealth or political power”*.

We conceptualize these informal notions by employing the fundamental notion of *influence* among groups of nodes, and proposing three independent properties related to the influence between the elite and the periphery, named *dominance*, *robustness* and *density*. (Note that our treatment of these notions expands on our preliminary results, presented in [17]. In particular, our results are now stated for general settings obeying a natural set of assumptions, and not just for an influence model based on edges, as done in [17]. Moreover, in Sect. 4 we now provide empirical support for our claims.) We also look at a fourth property, the elite *compactness*, and examine its implications.

It should be realized that our axioms are not claimed to hold for *every* core-periphery partition. Moreover, we do not claim that *every* social network has a core-periphery partition that satisfies the axioms. In fact, there are examples of both real complex networks and known evolutionary network models in the literature, where our axioms are not satisfied by *any* core-periphery partition. Our claim is, rather, that there exists a class of natural social networks that *do* admit core-periphery partitions that satisfy our axioms. This class is hereafter referred to as the class of *elite-centered* social networks. We provide empirical data supporting the claim that elite-centered social networks commonly exist in practice, and analyze the properties of these social networks, with a focus on the size of the elite.

**Fig 1:**
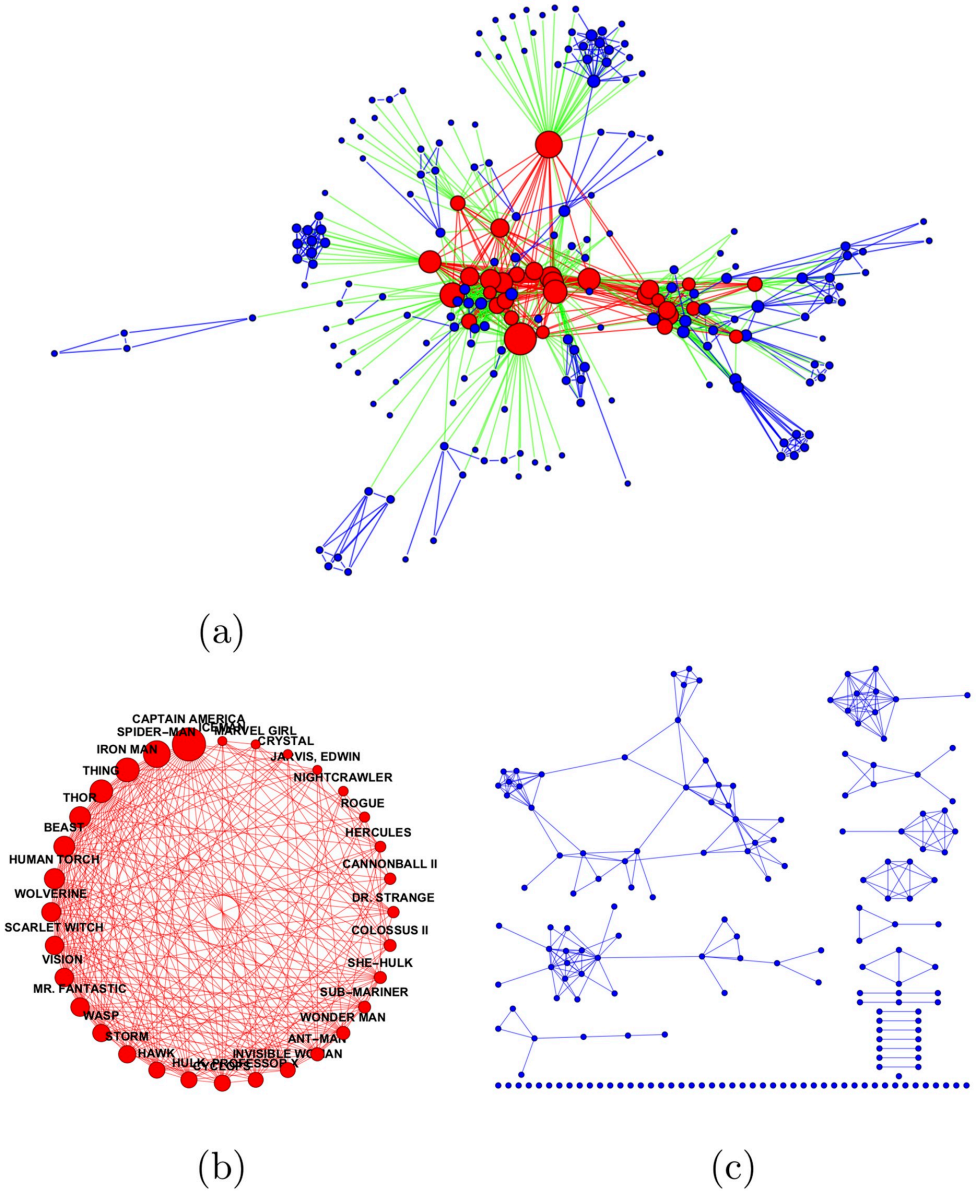
Fictional illustrative example: The social network of the Marvel’s superheroes. Two heroes are linked if they appeared together in at least 50 comic book titles [18]. (a) The network (208 superheroes, 912 edges), partitioned into an elite (red nodes and internal edges) and a periphery (green nodes and internal edges). Blue “crossing” edges connect elite and periphery nodes. (b) The (dense) elite subgraph (32 nodes, 259 edges). (c) The (sparser) periphery subgraph (176 nodes, 257 edges).

A small illustrative example of the terms we use is provided in Fig 1. It presents the network of the top 208 Marvel [18] superheroes and the 912 links interconnecting them, where two heroes are connected by a link if they appeared together in at least 50 comic book titles. The superheroes are partitioned into an elite and a periphery as shown by different node colors. The 32 superheroes with the highest degrees are assigned to the elite, the remaining 176 superheroes form the periphery. Two striking features can be clearly observed in this figure. First, the elite (containing, e.g., Captain America, Spiderman, and Thor), depicted in Fig 1(b), is dense and organized, while the periphery, presented in Fig 1(c), is much sparser and less structured. Second, despite their considerable size difference, the elite and the periphery have almost the same number of internal edges (259 and 257, respectively), namely, they are *balanced*. Moreover, the number of “crossing” edges connecting the core to the periphery is much larger (396), reaching most of the nodes in the periphery.

Are those properties an artifact of our selected example and partitioning method? Or are they *universal*, and should be expected to recur in many (or even most) networks? In particular, what are the rules that govern the elite size? We attempt to answer these questions in what follows.

We argue using our axioms, supported by evidence collected from real network data, that it is more accurate to consider 32 as about $\sqrt{912}$ in the Marvel example, and more generally, view the elite size as roughly the square root of the number of *edges* in the network. This is our “Price’s law” equivalent for elite size in social networks.

It should be clear that our axiomatic characterization does not provide a unique definition for the elite, nor does it lead to a single group being identified as the elite in a given social network. In fact, it seems unlikely that a single formal definition exists that suits all elite types in all social networks. However, it narrows down the range of subsets of society that are suitable candidates to be the elite, and moreover, the axioms are powerful enough to allow us to derive several conclusions concerning basic properties about the distribution of power and the size of the elite in society.

### 1.3 Balance point and balanced edge-based partition

It is not clear that the elite must be a unique subset of elements in a network. To illustrate this last point, let us consider the following mental experiment. Sort the members of society by decreasing order of influence. (The exact definition of “influence” is immaterial here, and will be discussed later.) Add the society members to a set $\hat{E}$ (representing the intended elite) one by one in this order. After the first step, $\hat{E}$ contains only one member of society, albeit the most influential one, hence clearly it cannot yet be thought of as “the elite”—it simply has insufficient power. This holds true also for the next few sets obtained in this way. On the other extreme, if the process is continued to its conclusion, we end up with $\hat{E}$ containing the entire society, which is clearly too large to be considered “the elite”. The question is therefore: at which point along this process does $\hat{E}$ qualify as an elite?

Intuitively, the “break-point” where the process should be halted is the point where adding new members into $\hat{E}$ no longer serves to significantly strengthen the group but rather “dilutes” its power relative to its size. Following Price’s principle, in this paper we propose a concrete choice for this break-point, referred to as the *balance point* of the elite. At its balance point, an elite-periphery partition exhibits a balance of influence of the elite and of the periphery on each other.

We show that partitioning the network at the balance point has nice properties. For concreteness, we focus on the special case where influence is represented by a large number of connections, or in other words by a high degree. We refer to this concrete choice as the *edge-based* interpretation of influence, or the edge-based influence model. Given this interpretation, we sort the nodes by their degrees in nonincreasing order (called the degree ordering *π*_*deg*_) and refer to the corresponding elite-periphery partition obtained at the balance point by the *balanced edge-based partition*. In other words, we build an elite of the *k* nodes with the highest degree, such the so-called *k*-rich club [19] is close to the balance point (*bp*). We demonstrate empirically that for partitions based on this ordering, the maximum number of crossing edges between the core and the periphery occurs in many cases at or near *bp*. (Note that other ordering methods can be used to analyze the properties of cores of different size. In particular, any node centrality can be used. In addition to the degree ordering presented in this article, we have also run our experiments for the ordering derived from the *c*-core decomposition [20] and results were very similar.) We also show theoretically that in the random configuration model [21], such a partition maximizes the expected number of crossing edges between the core and the periphery, for any ordering.

**Fig 2:**
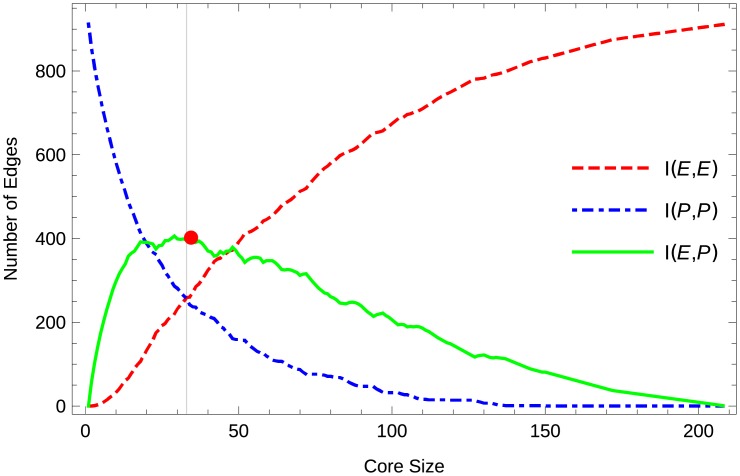
Illustration of the balance point of the Marvel superheroes network. The *influence shift diagram*, based on adding the 208 nodes to the core in order of their degree *π*_*deg*_, depicts the influence of the elite and periphery on themselves and each other with growing elite size. The balance point is noted by a grey line. At this point we choose the 32 high degree nodes at the *elite*. They hold the same power as the rest of the population. The red dot marks the maximum number of crossing edges.

To illustrate these points let us consider the diagram of the influence between the elite and periphery (called the *Influence Shift Diagram*) for growing rich clubs as the elite for the Marvel example in Fig 2. In this plot we can see the change of influence between the elite and the periphery for varying size, where we interpret the number of edges between sets of nodes *X*, *Y* as a measure of influence *I*(*X*, *Y*). We observe that the influence of the elite on the periphery is maximized at the balance point, *bp*, i.e., for the balanced edge-based partition, where the influence of the two partitions on themselves are equal.

### 1.4 Methods

The common approach to explaining empirical results on social networks is based on providing a new concrete (usually random) *evolutionary model* and comparing its predictions to the observed data. In contrast, we follow an *axiomatic approach* to the questions at hand. This approach is based on postulating a small set of axioms, capturing certain expectations about the network structure and the basic properties that an elite must exhibit in order to maintain its power in the society. We then use these axioms to infer some additional properties, such as bounds on the elite size.

Employing an axiomatic approach instead of providing an evolutionary model has two main advantages for studying social and complex networks. First, while a basic random model provides us with a mechanism that generates networks with properties similar to the ones observed empirically, a mechanism alone does not necessarily advance our understanding of the *meaning* of the phenomenon. In contrast, a suitable set of axioms attaches an “interpretation” or “semantics” to observed phenomena. The second advantage is that given the axioms, it becomes possible to draw conclusions using logical arguments. E.g., it may be possible to infer some information on the *asymptotic* behavior of a growing network, which is not always clear from empirical findings. As a consequence, the axiomatic approach is, in some sense, stronger than providing a particular model, since once agreeing on the axioms and their implications, *every* model should be consistent with them.

The underlying assumption is that the excess influence of the elite allows it on the one hand to control the rest of the population, and on the other to protect its members from being controlled by others outside the elite. We refer to these two properties as *dominance* and *robustness* respectively. In addition, the “wealth” is shared by the elite few, implying that on average, the elite members hold much more influence than individuals in the periphery. We refer to this property as *density*. We characterize *elite-centered* social networks as the class of social networks that admit core-periphery partitions satisfying these three properties (which are made more formal in what follows).

### 1.5 Contributions and organization

This paper makes the following contributions:

**Theoretical Results**
1. *Elite Axioms:* Based on the definition of influence in social networks, we introduce three axioms that capture crucial properties of elites and show their independence. The axioms concern the *dominance, robustness* and *density* of elites.
2. *Balance Point:* We prove (in Theorem 3.2) that elites satisfying the axioms of our model and the compactness property are at their power balance point. This implies that the power of the periphery is similar to that of the elite, or in other words, that as society evolves, the “natural” core-periphery partition maintains a balance between these two groups.
3. *Elite Size:* We analyze the *size* of the elite. The combination of the facts that society grows and that elites are denser than peripheries implies that the fraction of the total population size decreases with time. We prove this by showing that the size of a compact elite is a sublinear function of the population size if the axioms are satisfied. In general, elites of constant size can exist in societies where influence might be sharply asymmetric or unbounded. In contrast, we prove that in bounded and influence-symmetric social networks an elite cannot be smaller than roughly the square root of the total influence in the system. In particular, assuming the edge-based influence model, the elite size is $\Omega (\sqrt{m})$, where *m* is the number of network edges.

We stress again that our theoretical results (with the exception of the last statement) do not depend on the specific choice of using the edge-based influence model.

**Empirical Results** In addition to the theoretical results on elite axioms and properties, we studied 32 real networks, in order to examine the extent to which our axioms are manifested in reality, and check if there are core-periphery partitions that evolve over time according to the axioms. In particular we have following results

1. *Influence Shift Diagrams:* We observe that in most networks, the growing rich-club cores feature very similar behavior with respect to the influence between the core and periphery. Among other results, we show that the number of crossing edges is maximized at the balance point and provide an analytical explanation for this phenomenon under the configuration model. Furthermore, for completeness, we discuss network topologies that deviate from the majority of the networks under scrutiny.
2. *Temporal Analysis, balanced edge-based partition:* As a second step, we investigate how core-periphery partitions evolve over time to gain a deeper understanding of their asymptotic behavior. In particular, we study the core-periphery partition according to the degree ordering of nodes at its balance point, providing evidence for the existence of real elite-centered social networks, satisfying all axioms. Moreover, we compare this partition with partitions at other breakpoints.

The rest of this article is organized as follows. The next section presents our model of influence, the notion of influence shift diagrams, the balance point and the balanced edge-based partition. In Section 3 we introduce our axioms and derive the theoretical results of this article. Subsequently, in Section 4 we present our empirical study of the balanced edge-based partition in real world networks. Related work is provided in Section 5, and finally, we conclude with a discussion in Section 6.

## 2 Influence, shift diagrams, balance points and core-periphery partitions

### 2.1 An abstract influence model

We start by formally defining the notion of *influence*. We consider influence to be a measurable quantity between any two *groups* of the population, *X* and *Y*. The influence of *X* on *Y* is denoted by *I*(*X*, *Y*). The groups *X* and *Y* are not necessarily distinct, and we are also interested in the *internal influence* exerted by the nodes of a group *X* on themselves, referred to by *I*(*X*, *X*).

We assume that influence satisfies an *additivity property*, namely,

**(P1) Additivity:** *I*(*X* ∪ *Y*, *Z*) = *I*(*X*, *Z*) + *I*(*Y*, *Z*),
         *I*(*Z*, *X* ∪ *Y*) = *I*(*Z*, *X*) + *I*(*Z*, *Y*)).

We are interested in two special subclasses of social networks, exhibiting symmetric and bounded influence. Note that in general *I*(*X*, *Y*) ≠ *I*(*Y*, *X*). The subclass of *influence-symmetric* networks consists of networks in which influence satisfies the following *symmetry property*:

**(P2) Symmetry:** *I*(*X*, *Y*) = *I*(*Y*, *X*) for every *X*, *Y* ⊆ *V*.

Networks satisfying property (P2) are hereafter referred to as *influence-symmetric* networks. The next subclass of interest is the subclass of bounded influence. To begin with, in order to reflect the self-influence of every individual’s opinion on itself, and at the same time calibrate the measure of influence, we assume the following property.

**(P3) Self-influence:** *I*(*x*, *x*) = 1 for each individual *x*,
  i.e., self-influence is a basic unit of influence. (Throughout we slightly abuse notation by writing *x* instead of {*x*}.) Since we are interested in the *relative* (and not absolute) influence between individuals, we shall assume that all other influences are measured in terms of this unit.

Networks of bounded influence are ones where self-influence is at least as large as (a fraction of) the influence of any other individual, or formally, where the following property holds, for some positive constant *c*_*b*_:

**(P4) Bounded influence:** *I*(*x*, *y*) ≤ *c*_*b*_ for any two individuals *x* and *y*.

Networks satisfying properties (P3) and (P4) are hereafter referred to as *bounded* networks.

Note that the definitions here are more elaborate than, and slightly different from, those given in [17]. In particular, the introduction of properties (P1)-(P4) allows us to prove our main results in general, and not just for the edge-based model.

We define the *total power* exerted by a set X to be

$$\begin{array}{c} I(X)\;=\;I(X,X)+I(X,V\backslash X)\;. \end{array}$$ (1)

Specifically, denote the total amount of influence in the network by

$$\begin{array}{c} I_{tot}\;\equiv \;I\left(V\right)\;=\;I\left(V,V\right)\;. \end{array}$$

A *core-periphery* partition of a network is a pair $\left(\hat{C},\hat{P}\right)$ where $\hat{C}\cap \hat{P}=\emptyset$, $\hat{C}\cup \hat{P}=V$ and $\hat{C}$ is the smaller set. We denote the set of core nodes by $\hat{C}$ and the rest of society (the periphery) by $\hat{P}$ and study the four influence quantities $I(\hat{C},\hat{C})$, $I(\hat{P},\hat{P})$, $I(\hat{C},\hat{P})$ and $I(\hat{P},\hat{C})$.

### 2.2 Edge-based model of influence for social networks

In Section 3 we present a theoretical analysis of the properties of elites, which is based on the above abstract definition of influence. For the purpose of empirical analysis, however, it is convenient to employ a concrete interpretation and measure of influence. In a social network, a network edge represents some social relation between the two connected nodes, such as friendship, co-authorship, following on Twitter, etc. It is therefore natural to interpret influence using the edges, by simply stating that a directed edge (*x*, *y*) connecting the node *x* to the node *y* represents the influence *I*(*x*, *y*) of *x* on *y*. The amount of influence can be represented by associating a *weight* with each edge. (To represent the notion of self-influence discussed above, we assume that each node in the network has a *self-loop*, namely, an edge connecting it to itself, and its weight is 1.) It follows that under this interpretation, the total influence exerted by a node (or a group of nodes) equals the total weight of the edges emanating from it, and similarly, the total influence exerted on a node (or a group of nodes) equals the total weight of the edges entering it. Note that assuming the symmetry property (P2) is tantamount to assuming that the weights of the directed edges (*x*, *y*) and (*y*, *x*) are equal, or more simply, that the network is undirected. Hereafter, we refer to this interpretation of influence as the *edge-based influence model*, and use it to illustrate our result as well as for our empirical study.

It should be clear that focusing on one concrete interpretation involves a certain loss of generality, as certain aspects get fixed in a specific way. For example, note that under the edge-based interpretation of influence, non-adjacent individuals (namely, ones not directly connected by an edge) have zero influence. Needless to say, this is not necessitated by the notion of influence, and one may think of other natural interpretations that do not possess this property. For example, one may consider a *flow-based influence model*, where the influence between two nodes *x* and *y* is defined to be the maximal amount of *flow* that can be transferred between them in the network. If one is interested in a binary measure of influence, then it is possible to define the influence between *x* and *y* as 1 if the maximal flow between them exceeds some threshold *f*, and 0 otherwise. Clearly, such a *flow-based* interpretation and measure of influence is independent of the existence of edges, in the sense that it may assign an influence value of *I*(*x*, *y*) = 1 to some non-adjacent nodes *x* and *y*, or a value of *I*(*x*′, *y*′) = 0 to some adjacent *x*′ and *y*′. Nevertheless, we feel that for the sake of illustrating the concepts and notions introduced and discussed in this paper, it is useful to adopt a single concrete interpretation, as done herein with the edge-based influence model.

Formally, we model a social network as a directed, weighted graph *G* = (*V*, *E*, *ω*), with a set *V* of *n* nodes representing the members of society, connected by a set *E* ⊆ *V* × *V* of *m* directed edges, and a positive weight function ω:E→ℝ such that *ω*(*e*) > 0 for every *e* ∈ *E*. For a set of edges *E*′ ⊆ *E*, define the weight of *E*′ as *ω*(*E*′) = ∑_*e*∈*E*′_ *ω*(*e*). Given an undirected network, we consider each undirected edge as two equal weight directed edges of opposite directions. Given an unweighted network, we consider all edges to have weight one.

For every node *v* and set of nodes *X*, let the set of directed edges leading from *v* to nodes in *X* be denoted by *E*(*v*, *X*). Similarly, for node sets *X*, *Y* ⊆ *V*, let *E*(*X*, *Y*) denote the set of directed edges leading from nodes in *X* to nodes in *Y*. Relying on the edge weights, we define the influence of *X* on *Y*, for *X*, *Y* ⊆ *V*, as

$$\begin{array}{c} I(X,Y)\;=\;\omega (E(X,Y))\;. \end{array}$$ (2)

Given a core-periphery partition $\left(\hat{C},\hat{P}\right)$ of *V*, the edge set *E* can be partitioned into four disjoint edge sets $E(\hat{C},\hat{C}),E(\hat{C},\hat{P}),E(\hat{P},\hat{C})$ and $E(\hat{P},\hat{P})$. Looking at the *adjacency matrix* *A*(*G*) of the core-periphery network *G* [22], these sets correspond to the four basic parts of the *block-model representation* [23] of *A*(*G*). The matrix *A*(*G*) can now be written as in the following figure.

$$A\left(G\right)=[\begin{array}{cc} \begin{array}{c} E(\hat{C},\hat{C}) \end{array} & \begin{array}{c} E(\hat{C},\hat{P}) \end{array}\\ \begin{array}{c} E(\hat{P},\hat{C}) \end{array} & \begin{array}{c} E(\hat{P},\hat{P}) \end{array} \end{array}]$$

Note that in this case $I_{tot}=I(\hat{C},\hat{C})+I(\hat{C},\hat{P})+I(\hat{P},\hat{C})+I(\hat{P},\hat{P})$.

### 2.3 Influence shift diagram

As a fundamental tool for our analysis we consider the *influence shift diagram*. Consider an influence-symmetric social network *G*(*V*, *E*) and assume some suitable ordering *π* of the nodes of *V*, reflecting node influence, centrality, connectivity or any other measure of power. Recall the gradual core building process mentioned in the introduction. It starts with the core defined as the empty set, and the periphery containing all the nodes of the network, namely, ${\hat{C}}_{0}^{\pi }=\emptyset$ and ${\hat{P}}_{0}^{\pi }=V.$ One by one, move the nodes from the periphery to the core, according to the assumed ordering *π*, starting with the highest ranked nodes. As this transition evolves, and the core ${\hat{C}}_{k}^{\pi }$ after *k* iterations consists of *k* nodes, the total influence of the core and the periphery undergo a gradual shift, where the number of internal edges $I({\hat{C}}_{k}^{\pi },{\hat{C}}_{k}^{\pi })$ increases, the number of internal edges $I({\hat{P}}_{k}^{\pi },{\hat{P}}_{k}^{\pi })$ decreases, and the number of crossing edges $I({\hat{C}}_{k}^{\pi },{\hat{P}}_{k}^{\pi })$ first increases and then decreases. The influence shift diagram presents these changes in $I({\hat{C}}_{k}^{\pi },{\hat{C}}_{k}^{\pi })$, $I({\hat{P}}_{k}^{\pi },{\hat{P}}_{k}^{\pi })$ and $I({\hat{C}}_{k}^{\pi },{\hat{P}}_{k}^{\pi })$ graphically as the core ${\hat{C}}_{k}^{\pi }$ grows from its minimum size of 1 to its maximum size of *n*. Let us remark that influence shift diagrams can be used also for directed (asymmetric) networks. In that case the plots will include $I({\hat{C}}_{k}^{\pi },{\hat{P}}_{k}^{\pi })$ and $I({\hat{P}}_{k}^{\pi },{\hat{C}}_{k}^{\pi })$. In this work we focus on undirected networks where $I({\hat{C}}_{k}^{\pi },{\hat{P}}_{k}^{\pi })=I({\hat{P}}_{k}^{\pi },{\hat{C}}_{k}^{\pi })$.

Formally, we use the following definition.

**Definition 2.1** (Influence Shift Diagram). *Let π be a total order over the n nodes of the graph G*(*V*, *E*). *Denote the k*^*th*^ *ranked node according to the order π by v*_*k*_. *The k-core according to π is* ${\hat{C}}_{k}^{\pi }=\{v_{1},v_{2},...,v_{k}\}$. *The k-periphery according to π is* ${\hat{P}}_{k}^{\pi }=\{v_{n},v_{n-1}...,v_{n-k}\}$. *The Influence Shift Diagram, ISD*(*G*, *π*), *over the graph G and the order π, consists of the three functions* $I({\hat{C}}_{k}^{\pi },{\hat{C}}_{k}^{\pi })$, $I({\hat{P}}_{k}^{\pi },{\hat{P}}_{k}^{\pi })$ *and* $I({\hat{C}}_{k}^{\pi },{\hat{P}}_{k}^{\pi })$ *for k* = 1 … *n*.

When the order *π* is clear from the context, we omit the superscript *π* from ${\hat{C}}_{k}$ and ${\hat{P}}_{k}$ to increase readability.

To facilitate a comparison of influence shift diagrams across networks with different numbers of nodes and edges, we need to normalize the X and Y axes in a meaningful way. There are several plausible options for scaling of the X-axis when plotting an influence shift diagram. The simplest one is a node-normalized linear scale, i.e., $x=\frac{\left|\hat{C}\right|}{n}$, which represents the relative size of the core with respect to the number of nodes in network. Another option is a logarithmic scale, where a point *x* represents a core of size $|\hat{C}|=n^{x}$ so $x=\frac{\text{log}|\hat{C}|}{\text{log}n}$. A third option is a normalized influence scale, $x=I(\hat{C})/I_{tot}$. To scale the values for the *Y*-axis in a consistent way, we let it represent the fraction of the total influence contained in the relevant influence sets, namely, the values of $I(\hat{C},\hat{C})/I_{tot}$, $I(\hat{P},\hat{P})/I_{tot}$, and $I(\hat{C},\hat{P})/I_{tot}$ respectively. (Recall that under the edge-based interpretation, *I*_*tot*_ = *m*).

Thus, it holds for all of these scaling options that *x*, *y* ∈ [0, 1]. This enables us to compare networks of different size and calculate their mean easily. The choice of the preferred scale is dictated by the phenomenon that we wish to explore, thus making the influence shift diagram a useful analysis tool.

**Fig 3:**
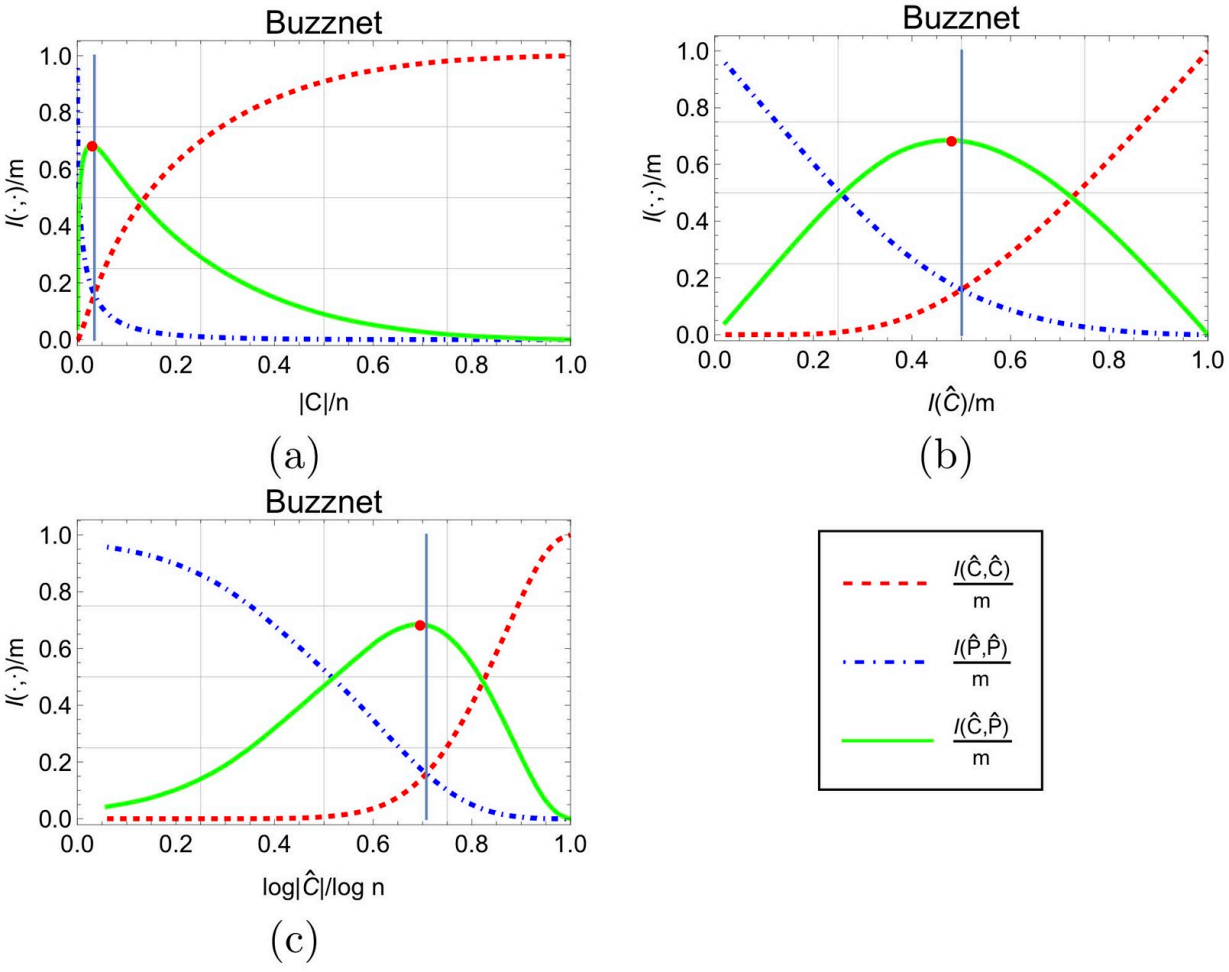
Influence shift diagram of Buzznet, containing *n* = 101, 163 nodes and *m* = 2, 763, 066 edges. $I({\hat{C}}_{k})=I({\hat{P}}_{k})$, for the balance point *k* = 3, 472. (a) Using the node-normalized scale, the balance point *k* = 3.4% is far to the left and it is hard to study how the core and periphery influence each other around it. (b) In the plot with the normalized influence scale (under the edge-based interpretation) the balance point is right at the center, while the third plot with a logarithmic scale (c) hints at the asymptotic behavior *k* = *n*^0.707^. The balance point is marked by a grey line and the red dot marks the maximum number of crossing edges.

Fig 3 depicts examples of influence shift diagrams of a real social network for the different X-axis scaling methods. It uses the degree for the ordering of nodes (*π*_*deg*_), i.e., the elite corresponds to a *k*-rich club, containing the *k* highest degree nodes.

### 2.4 Balance point and balanced edge-based partition

Core-Periphery partitions of a given complex network are used to reveal the basic structural properties of the network. A simple approach to selecting a partition for a given social network *G*(*V*, *E*) requires a suitable stopping criterion for the process presented above: given some ordering *π* on the nodes of *V*, the top *k* nodes in this ordering would then constitute the core, yielding ${\hat{C}}_{k}^{\pi }$, the *k*-core according to *π* (see Def. 2.1). However, it is still necessary to identify an appropriate break-point *k*.

Our proposal for a suitable break-point relies on the key notion of a *balanced* distribution of power between the core and the periphery. Aiming for such a balance, a natural break-point for a given ordering *π*, referred to as the *π-balance point*, would be the *k*-core-periphery partition $\left({\hat{C}}_{k}^{\pi },{\hat{P}}_{k}^{\pi }\right)$ satisfying that

(1) $I({\hat{C}}_{k}^{\pi })\geq I({\hat{P}}_{k}^{\pi })$, but
(2) $I({\hat{C}}_{k-1}^{\pi })<I({\hat{P}}_{k-1}^{\pi })$

We say that the partition $\left({\hat{C}}_{k}^{\pi },{\hat{P}}_{k}^{\pi }\right)$ is *at the π-balance point*, and summarize the relations (1) and (2) by writing $I({\hat{C}}_{k}^{\pi })\overset{\pi }{\approx }I({\hat{P}}_{k}^{\pi })$.

While the above definition provides a *local* balance point appropriate for a specific ordering *π*, we are also interested in a more global notion of balance, applicable to all orderings. This leads us to the following definition.

**Definition 2.2** (Global Balance Point). *A given core-periphery partition* $\left(\hat{C},\hat{P}\right)$ *is at a* global balance point *iff*

(1) $I(\hat{C})\geq I(\hat{P})$, *but*
(2) *for every* ${\hat{C}}^{\prime }$ *such that* ${\hat{C}}^{\prime }\subset \hat{C}$, *the partition* $\left({\hat{C}}^{\prime },{\hat{P}}^{\prime }\right)$ *satisfies* $I({\hat{C}}^{\prime })<I({\hat{P}}^{\prime })$.

*We summarize* (1) *and* (2) *by writing* $I(\hat{C})\overset{\Delta }{\approx }I(\hat{P})$.

We are also interested in partitions that are not at the global balance point, or at the *π*-balance point for any particular ordering *π*, but are nevertheless close to being balanced. The partition $\left(\hat{C},\hat{P}\right)$ is said to be *near* its balance point, denoted $I(\hat{C})\approx I(\hat{P})$, if $I(\hat{C})=\Theta (I_{tot})$ and $I(\hat{P})=\Theta (I_{tot})$. (The Θ()-notation refers to asymptotic behavior as the network size grows).

Note that for influence-symmetric networks, by the symmetry property (P2), $I(\hat{C},\hat{P})=I(\hat{P},\hat{C})$, thus by Eq (1)

$$\begin{array}{c} I\left(\hat{C}\right)=I\left(\hat{P}\right)\;\;\Rightarrow \;\;I\left(\hat{C},\hat{C}\right)=I\left(\hat{P},\hat{P}\right). \end{array}$$

Note also that for a given network there are many partitions at or near a balance point. In fact, for each ordering *π* of the nodes there is a *k*-core ${\hat{C}}_{k}^{\pi }$ such that the partition $\left({\hat{C}}_{k}^{\pi },{\hat{P}}_{k}^{\pi }\right)$ is at its *π*-balance point, as discussed above.

#### The balance point in edge-based partitions

We are particularly interested in the special case of the edge-based influence model with the degree ordering *deg*. In fact, our experiments focus on this model: we apply it to real-world networks and study the resulting *rich club* core-periphery partition at the balance point of this ordering, which we denote by *bp*. This partition is defined formally as follows.

**Definition 2.3** (Balanced Edge-Based Partition). *The* balanced edge-based partition *of a network is the partition* $\left({\hat{E}}^{*},{\hat{P}}^{*})\;=\;({\hat{C}}_{bp}^{deg},{\hat{P}}_{bp}^{deg}\right)$ *at the balance point bp with respect to the edge-based influence model and the degree ordering deg*.

It is important to observe that this particular setting yields a partition $\left({\hat{E}}^{*},{\hat{P}}^{*}\right)$ that is not only locally balanced (w.r.t. the ordering *deg*), but also *globally balanced* (according to Def. 2.2). Formally, we make the following observation.

#### Fact

The balanced edge-based partition $\left({\hat{E}}^{*},{\hat{P}}^{*}\right)$ is both a (local) *deg*-balance point, satisfying $I({\hat{E}}^{*})\overset{deg}{\approx }I({\hat{P}}^{*})$, and a global balance point, satisfying $I({\hat{E}}^{*})\overset{\Delta }{\approx }I({\hat{P}}^{*})$.

We use the notation ${\hat{E}}^{*}$ (instead of ${\hat{C}}^{*}$) to emphasize that we use this partition to define an *elite* for the social network.

In the graphic presentation of our empirical results, the choice of the X-axis to represent $I({\hat{C}}_{bp}^{\pi })/I_{tot}$ aligns the balance point to *x* = 0.5. This alignment allows us to focus on the balance point, observe its properties, and examine how the core and periphery influence measures behave around this point. We mark the balance point in influence shift diagrams by a vertical line (and when relevant, a red point marks $\text{max}(I(\hat{C},\hat{P}))$), see Fig 3.

As we will see later, the Balanced Edge-Based Partition of many real world social networks have interesting properties. The elite axioms formulated in the next section can help to explain these.

## 3 Elite axioms

### 3.1 Dominance, robustness and density

The three simple axioms presented in this section capture basic structural properties required of the core-periphery partition $\left(\hat{E},\hat{P}\right)$ in *elite-centered* social networks. To emphasize our focus on networks whose core is an elite, we denote the core set of the partition by $\hat{E}$ rather than $\hat{C}$ where the core is a social elite.

In order to state our three axioms, we first define three corresponding measures for quantifying the *dominance*, *robustness*, and *density* of an elite $\hat{E}$ in a $\left(\hat{E},\hat{P}\right)$ partition.

**Dominance:** This measure concerns the balance between forces exerted on the *periphery*, that is, it compares the influence of the elite on the periphery with the internal influence of the periphery. Formally, for a given $\left(\hat{E},\hat{P}\right)$ partition

$$\begin{array}{c} \text{dom}\left(\hat{E}\right)=I\left(\hat{E},\hat{P}\right)/I\left(\hat{P},\hat{P}\right). \end{array}$$

The first axiom states the following.

**(A1) Elite-dominance:** $\text{dom}\left(\hat{E}\right)\geq c_{d}$, for a fixed constant *c*_*d*_ > 0

This essentially says that the elite *dominates* the rest of society, in the sense that the *external* influence maintained by the elite $\hat{E}$ on the periphery $\hat{P}$ is higher, or at least not significantly lower, than the *internal* influence of the periphery on itself. Intuitively, the elite must have high dominance in order to maintain its superior status in society.

We remark that our definition of dominance is different from the notion of domination commonly used in graph theory. Furthermore, while dominance is related to the concept of conductance (also known as Cheeger’s constant), the two measures are not identical, as the terms appearing in their denominators are different (see, e.g., the definitions in [24, 25]).

**Robustness:** The robustness measure concerns the forces exerted on the *elite*, and compares the internal influence of the elite with the influence of the periphery on the elite. Formally, for a given $\left(\hat{E},\hat{P}\right)$ partition

$$\begin{array}{c} \text{rob}\left(\hat{E}\right)=I\left(\hat{E},\hat{E}\right)/I\left(\hat{P},\hat{E}\right). \end{array}$$

The second axiom states the following.

**(A2) Elite-robustness:** $\text{rob}\left(\hat{E}\right)\geq c_{r}$, for a fixed constant *c*_*r*_ > 0

This axiom ensures that elite is *robust*, and is capable of maintaining its cohesiveness, sticking to its opinions, and resisting “outside” pressure (in the form of the periphery’s external influence). This is guaranteed by requiring that the *internal* influence of the elite $\hat{E}$ on itself is greater, or at least not significantly less, than the *external* influence exerted on $\hat{E}$ by the periphery.

**Density:** This measure concerns the *disproportionality* between the elite’s *power* and *size*. Let

$$\begin{array}{c} \delta \left(X\right)=\frac{\log I(X)}{\log|X|} \end{array}$$

denote the *log-density* of a set *X* ⊆ *V*. (Note that this measure is similar to (but slightly stronger than) the notion of the average degree of a set of nodes *X*, which is formally defined as (*w*(*E*(*X*, *X*)) + *w*(*E*(*X*, *V* \ *X*))) / |*X*| = *I*(*X*) / |*X*|.) Then, for a given $\left(\hat{E},\hat{P}\right)$ partition

$$\begin{array}{c} \text{dens}\left(\hat{E}\right)=\frac{\delta (\hat{E})}{\delta (V)}. \end{array}$$

The third axiom states the following.

**(A3) Elite-density:** $\text{dens}\left(\hat{E}\right)\geq 1+c_{c}$, for a fixed constant *c*_*c*_ > 0

This axiom (named “elite compactness” in [17]) says that the subnetwork spanned by the elite is denser than the entire network. The implication is that on average, an elite member holds significantly more power than an arbitrary member of society.

**Fig 4:**
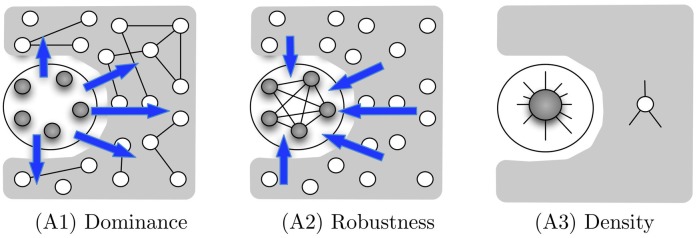
Graphical illustration of the three axioms. Elite nodes are gray. (A1) The elite’s external influence (blue edges), $I(\hat{E},\hat{P})$, dominates the periphery’s internal influence (black edges), $I(\hat{P},\hat{P})$. (A2) The internal influence of the elite, $I(\hat{E},\hat{E})$, is robust to the periphery’s external influence, $I(\hat{P},\hat{E})$. (A3) The elite is denser and its average individual is more powerful than an average individual in the society.

The three axioms are illustrated graphically in Fig 4. We say that a family of *n*-node networks *G*_*n*_, for growing *n*, satisfies the axiom *A* if there exists some *n*_0_ such that *G*_*n*_ satisfies *A* for every *n* ≥ *n*_0_.

We also examine the impact of another possible property of elites. The members of an elite often strive to maintain their status by keeping the elite as small as possible. This tendency conflicts with the need to maintain the elite’s robustness and dominance. Consequently, these conflicting needs may yield convergence to a natural equilibrium point, characterized by the following property.

**(P5) Elite-compactness:** The elite is a *minimal* set of individuals that satisfies the dominance and robustness axioms (A1) and (A2).

While we do not assume this property to be an axiom (in fact, it was not considered in [17]), we are still interested in its implications. In fact, we show that whenever this property holds, the system attains a *balance point*, where the elite and the periphery have about equal total power. One may argue that the dynamic forces that generate the social structure of elite and periphery cause it to converge to this balance point: going below this point will endanger the elite’s robustness or dominance; going above it will yield an “inefficient” elite, namely, one that is larger than is absolutely necessary.

### 3.2 Axiom independence

We begin our analysis by showing that the three axioms are independent of each other, that is, for any two axioms out of the three, there exist a social network and a core-periphery partition that satisfies the two axioms but not the third.

**Theorem 3.1** (Axiom independence). *Axioms* (*A*1), (*A*2), (*A*3) *are independent, namely, assuming any two of them does not imply the third*.

**Fig 5:**
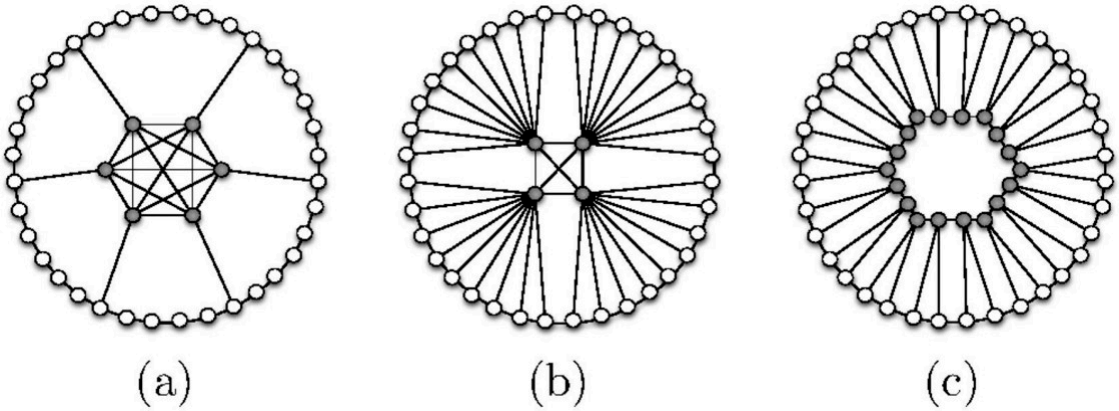
Network examples demonstrating the independence of the axioms (the gray nodes form the core). Here we use the edge-based influence model. (a) The core is robust and dense but not dominant. (b) The core is dominant and dense but not robust. (c) The core is dominant and robust but not dense. (d) An example of a network satisfying all three axioms.

See Appendix A.1 for the proofs of the theorems in this section. Fig 5(a)–5(c) depicts networks that illustrate Theorem 3.1. Each of them satisfies exactly two of the three axioms. Note that there are certain networks for which no core-periphery partition satisfies all three axioms (A1), (A2), and (A3) simultaneously.

Interestingly, in the special case of influence-symmetric networks, axioms (A1) and (A2) are “inversely dependent”, namely, every influence-symmetric network and every core-periphery partition must satisfy at least one of them. (More precisely, if the partition $\left(\hat{C},\hat{P}\right)$ disobeys both axioms (A1) and (A2), then the dual partition $\left(\hat{P},\hat{C}\right)$, with $\hat{P}$ as the core and $\hat{C}$ as the periphery, satisfies both (A1) and (A2), with different constants.) This implies that there are no influence-symmetric networks that disobey all three axioms.

### 3.3 Power distribution

We can now use our axioms to provide bounds for the influence of the elite and periphery on each other and for the elite size. Recall that the class of *elite-centered social networks* consists of social networks that admit a core-periphery partition satisfying all three axioms. For such networks we can draw the following result about the connection between the axioms and the compactness property on the one hand, and the balance point on the other.

**Theorem 3.2** (Balance Point). *Let* $\left(\hat{E},\hat{P}\right)$ *be a core-periphery partition that satisfies the dominance, robustness and density axioms (A1), (A2), (A3), and the compactness property (P5). Then the partition is near the balance point, i.e.*, $I(\hat{E})\approx I(\hat{P})$, *or*, $I(\hat{E})=\Theta (I_{tot})$ *and* $I(\hat{P})=\Theta (I_{tot})$. *Specifically, using the edge-based influence model, both* $I(\hat{E})\;=\;\Theta (m)$ *and* $I(\hat{P})\;=\;\Theta (m)$.

This means that for any elite that satisfies Axioms (A1), (A2), (A3) and property (P5), the overall influence of the elite, $I(\hat{E})$, is (nearly) equal to the overall influence of the periphery, $I(\hat{P})$, which makes the elite-periphery *power balance* a universal property. In other words, an elite that satisfies the axioms must be close to the balance point. We expect this “balance of powers” between the elite and the periphery to be recognized as a significant element in understanding the internal balances in social networks.

### 3.4 Elite size

Our next theorem concerns the size of the elite. It shows that elite-centered social networks have a sublinear elite. This is formalized as follows.

**Theorem 3.3** (Elite Size). *Let* $\left(\hat{E},\hat{P}\right)$ *be a core-periphery partition that satisfies the dominance, robustness and density axioms (A1), (A2) and (A3). Then the elite size is* sublinear *in the size of society, namely*,

$$\begin{array}{c} c\cdot n^{\frac{\delta (V)}{\delta (\hat{E})}}\leq \left|\hat{E}\right|\leq n^{\frac{1}{1+c_{c}}}\;. \end{array}$$

Note that the above discussion leaves open the question of upper bounding the size of a “real” elite. As our results provide only asymptotic bounds, it is impossible to ascertain whether the “universal” size of elites (if it exists) converge to a linear or sublinear function of the network size. For illustration, consider the US population of about 314 million people. An elite with a linear size of 0.1% of the whole population will consist of 314,000 people, while an elite of sublinear size, e.g., $n^{1/2}=\sqrt{314M}$, will consist of only about 18,000 people. These numbers differ by an order of magnitude; which of them is more plausible?

For growing networks, the above Theorem implies that the fact that elites are better connected than peripheries leads to the often observed phenomenon that the fraction of the total population size decreases with time. This is due to the fact that the elite size is a sublinear function of the population size if the axioms are satisfied.

In Section 4 we present evidence that many social networks are elite-centered (namely, satisfy our axioms), which implies elites of sublinear size are common.

Interestingly, if Axiom (A3) does not hold, then it is possible for the elite to be of linear size. This will be the case, for example, in social networks where Dunbar’s theory holds [26]. Dunbar found a correlation between primate brain size and average social group size, and of people with whom one can maintain stable social relationships. Since then, this limit has been estimated to fall between 100 and 250, with a commonly used value of 150. If this is the case, then one can show that an elite that satisfies Axioms (A1) and (A2) *must* be of linear size. One can even claim a slightly stronger result, stating that the elite’s average degree is bounded from above by a constant times the average degree in the network (which necessitates linear elite size).

Formally, we state the following.

**Lemma 3.4** (Large Cores). *Consider an influence-symmetric network. Let* $\left(\hat{E},\hat{P}\right)$ *be a core-periphery partition that satisfies the dominance (A1) and robustness (A2) axioms. If there exists some constant c, s.t*.

$$\begin{array}{c} I\left(\hat{E}\right)/\left|\hat{E}\right|\leq c\cdot I\left(V\right)/\left|V\right|, \end{array}$$

*then* $|\hat{E}|\geq c_{3}\cdot n$ *for some constant c*_3_.

We now turn lower bounds on the elite size. How small can the elite be while still maintaining its power and satisfying the axioms? Here we again focus on the edge-based influence model. Let us first observe that in the general case of asymmetric networks (allowing unequal influences between two individuals, such as in twitter for example), or in unbounded networks (violating the bounded influence property (P4) above), no nontrivial lower bounds hold, and the network may have an extremely small elite (possibly even constant size) that satisfies our axioms.

**Lemma 3.5** (Constant Elite Size). *In an asymmetric or unbounded network, there may exist core-periphery partitions* $\left(\hat{E},\hat{P}\right)$ *that satisfy the dominance, robustness and density axioms (A1), (A2) and (A3), where the elite* $\hat{E}$ *has a constant number of members*.

In the proof of Lemma 3.5, we observe that elites of constant size can only exist in societies with extremely asymmetric influence distributions, either by the weights or the direction of the influence relationship.

At the other extreme, consider the typical situation occurring in networks where social interactions are direct and personal. In such networks the influence relationship is usually bi-directional and the “quality” of this influence is similar across different influence pairs, or there is a bounded difference between the maximum and minimum weight. More formally, such networks are influence-symmetric and bounded (i.e., satisfy properties (P2), (P3) and (P4)). For such networks, a much higher lower bound for the elite size can be established. More precisely, the elite size must be at least in the order of the square root of the total influence in the network (and under the edge-based influence model, the square root of *m*, the number of edges).

**Theorem 3.6** (Square Root Lower Bound). *Consider an influence-symmetric and bounded network of m edges. Let* $\left(\hat{E},\hat{P}\right)$ *be a core-periphery partition that satisfies the dominance, robustness and density axioms (A1), (A2), (A3). Then the size* $\left|\hat{E}\right|$ *satisfies* $|\hat{E}|\geq c_{4}\cdot \sqrt{I_{tot}}$ *for some constant c*_4_ > 0. *Specifically, for the edge-based influence model we get* $|\hat{E}|\geq c_{4}\cdot \sqrt{m}$.

One can show an example of what we call a *purely elitistic society* where, under the edge-based influence model, the elite reaches its minimum possible size of $\Theta (\sqrt{m})$ in influence-symmetric and bounded (i.e., undirected and unweighted) networks. See Fig 5(d) for an illustrative example. In this network, the elite is formed by a clique of five nodes, each connected to five nodes of the periphery. In the periphery are connected to two other nodes in the periphery. More generally, we can construct similar networks of *n* nodes with an elite being clique of $\sqrt{n}$ nodes, each connected to $\sqrt{n}-1$ nodes of the periphery. The total number of edges in such networks is Θ(*n*) and in this scenario the dominance, robustness and density constants are 1.

We find it remarkable that these simple and intuitive assumptions lead to such a strong implication on the total power distribution and the elite size. Note that Theorem 3.3 is controversial to the common belief that the elite size converges to a linear fraction of a society’s size (most recently claimed to be 1% [27]). This discrepancy may perhaps be attributed to the fact that our axiom-based approach characterizes the elite differently than in previous approaches.

## 4 Empirical results: Balanced edge-based partition

In addition to the theoretical results on elite axioms and properties, we also studied some real networks, in order to examine the extent to which our axioms manifest in reality, and provide evidence for the existence of real elite-centered social networks.

We investigate both the static and dynamic properties of the balanced edge-based partition of real networks. In total we analyzed 32 social networks (the full list is available at S1 Supporting Information). Their observed behavior w.r.t. core and periphery relationships is surprisingly consistent. We begin by investigating influence shift diagrams followed by providing evidence that the balanced edge-based partition of many social networks satisfy our axioms, and therefore, their implication about the asymptotic size of the elite apply.

### 4.1 The shape of the influence shift diagram

**Fig 6:**
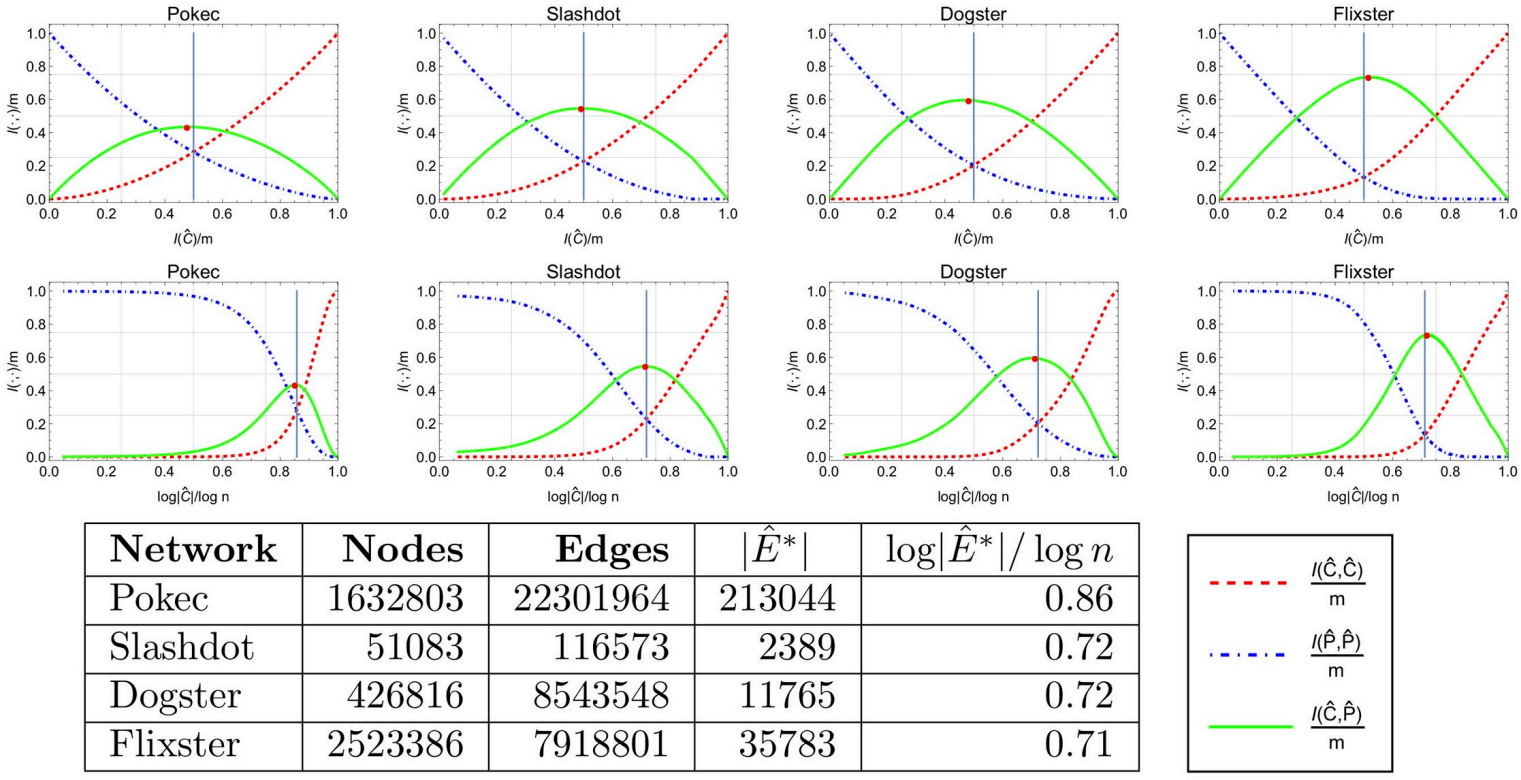
The influence shift diagrams for the degree ordering of four example networks, plotting the influence ratios ($I(\hat{C},\hat{C})/m$, $I(\hat{P},\hat{P})/m$ and $I(\hat{C},\hat{P})/m$). The diagrams in the top row feature an edge-normalized x-axis where the balance point is at *x* = 0.5. In the second row a logarithmic axis, where *x* = 0.5 represents $k=\sqrt{n}$, is used. The last row contains network details for the balanced edge-based partition.

For each social network in our data set we examined the influence of the core and periphery. To this end, we plot their influence shift diagrams for the degree ordering, *π*_*deg*_, presenting the changes in $I({\hat{C}}_{k},{\hat{C}}_{k}),I({\hat{P}}_{k},{\hat{P}}_{k})$ and $I({\hat{C}}_{k},{\hat{P}}_{k})$ as the core ${\hat{C}}_{k}$ (i.e., the *k*-rich club) grows from its minimum size, 1, to its maximum size, *n*. Fig 6 shows the influence shift diagrams of the *k*-rich-club of four example networks. The influence shift diagrams of all networks we studied can be found in S1 Supporting Information. Here, we present the plots of two different scaling methods for the *X*-axis. The top row uses the edge-normalized influence scale where the balance point is at *x* = 0.5. In the second row, the diagrams are plotted for a logarithmic scale where *x* = 0.5 corresponds to $I(\hat{C})=\sqrt{n}$. (Plots with a node-normalized scale can be found in S1 Supporting Information for completeness).

The influence shift diagrams for most networks exhibit a similar pattern: as the rich-club size *k* grows, the value $I({\hat{C}}_{k},{\hat{E}}_{k})/m$ grows from 0 to 1, and the value $I({\hat{P}}_{k},{\hat{P}}_{k})/m$ decreases from 1 to 0. The number of “crossing” edges (connecting the rich-club and the periphery), $I({\hat{C}}_{k},{\hat{P}}_{k})$, grows with *k* up to some maximum, and then starts decreasing. An interesting and less obvious pattern is that $I({\hat{C}}_{k},{\hat{P}}_{k})$ grows faster than $I({\hat{C}}_{k},{\hat{C}}_{k})$ right from the beginning, and remains larger until the maximum point. The relation between these numbers changes only after $I({\hat{C}}_{k},{\hat{P}}_{k})$ begins to decrease, while $I({\hat{C}}_{k},{\hat{C}}_{k})$ continues to grow. This seems to imply that during their growth, rich clubs acquire external edges faster than internal edges.

The consistency in the shapes of the influence shift diagrams for the edge-normalized scale for different networks, also enables some analytical observations which we will discuss later in the section.

One limitation of plots with an edge-normalized scale, is the fact that the size of the elite at the balance point is not revealed. On the other end, the logarithmic scale, enables us to examine this aspect the balance point more closely. Recall that the *balance point*, is the size *bp* where the internal influences of the core and the periphery are roughly equal; formally, $I({\hat{C}}_{bp},{\hat{C}}_{bp})\overset{deg}{\approx }I({\hat{P}}_{bp},{\hat{P}}_{bp})$. Graphically, *bp* is the point where the red (dashed) and blue (dot-dashed) lines intersect in Fig 6.

**Fig 7:**
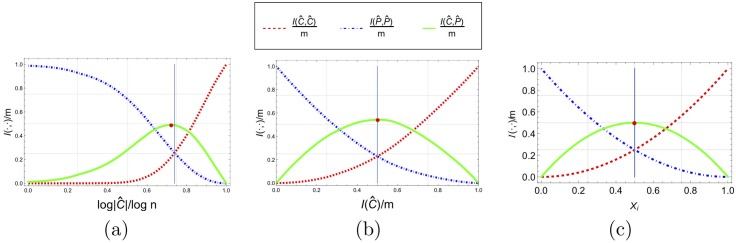
The average influence shift diagrams for the balanced edge-based partition of all 32 networks examined in our study (a) using the logarithmic scale axis (b) using the edge-normalized axis and (c) the closed form shape of the expected influence shift diagrams of the configuration model.

We summarizes the influence shift diagrams of all 32 networks in Fig 7 where we show the average results for all of the 32 networks in our experiments. Fig 7(a) present the average of the influence shift diagrams of all networks using the logarithmic scale. From this figure we see that the average exponent of the elite size at the balance point, i.e., when $I(\hat{E})=n^{x}$, is *x* = 0.75. For most of the networks in our dataset the balanced edge-based partition core size lies between *n*^0.6^ and *n*^0.85^, indicating a sublinear size of the elite. Fig 7(b) and 7(c) facilitates an analytical understanding of the shape of the influence shift diagram. The figure compares the average influence shift diagrams of our networks using the edge-normalized scale to the expected influence shift diagrams of a well known random model for social networks, the random configuration model [21]. The expected shape of the random configuration model can be analyzed and presented in a closed form, see Appendix B for details. The similarity of these two figures is striking. This may indicate that most of the edges in real networks are, in some sense, distributed similarly to the random configuration model. They are “like” random edges, making the influence shift diagram behave in a “nice” an expected way. Defining this more formally is deferred to future work.

Two important additional observation are in place. First we note that the shape of the influence shift diagram does not have to behave “nice”. In Appendix 4.3 we examine theoretically and empirically networks with different trends. The second observation is related to the maximal “crossing” edges between the core and periphery. One of the most interesting properties of partitioning the network at the balance point is that in most networks, the maximal number of crossing edges occurs at the balance point or near it. We mark this maximum point, $\text{max}(I({\hat{C}}_{k},{\hat{P}}_{k}))$, by a red dot on the influence shift diagram. We discuss this farther in Appendix C for the configuration model. We note that this topics offers many opportunities for explorations in other generative network models and other orderings for an empirical investigation beyond the scope of this work.

Returning to Fig 7, we highlight again that Fig 7(b) and 7(c) do not reveal the size of the elite at the balance point. To understand the asymptotic size of elites better we must turn to growing networks.

### 4.2 Axiom stability and elite size in growing networks

Recall that Theorems 3.2 and 3.3 state that any elite-centered partition, i.e., a partition containing a core that satisfies Axioms (A1), (A2), and (A3) asymptotically, is close to the balance point and that the core must be of sublinear size. In the previous section we presented evidence that the balanced edge-based partition representing rich-clubs at the balance point generates a relatively small elite. Although this observation concerns networks of different sizes, all of them were static, i.e., made from a network snapshot at a single time instance. Thus one cannot answer the question of the *asymptotic* size of the elite of networks of increasing size with certainty. Hence to validate our axioms and study the asymptotic size of the elite, it is necessary to turn to networks that grow over time. This requires available data on the creation time of every node and edge in the network. Some network data includes edges time stamps for the point in time when edges are added or removed. For those we can examine how the elite metrics vary over time for the balanced edge-based partition $\left({\hat{E}}^{*},{\hat{P}}^{*}\right)$. If the axioms are indeed satisfied as the network evolves over time, then we may draw conclusions about the *asymptotic* size of elites.

Thus, we evaluated 5 networks for which information was available about the creation time of each edge. These include the coauthorship graph of DBLP, the trust network of Epinions, a friendship network from Facebook, a network formed by Wall-To-Wall communication on Facebook, the friendship network of Flickr, and the reply network among Slashdot users. The full details of the datasets are available in S1 Supporting Information. Using this information, we simulated the evolution of each network over time. As data on the appearance time of each network node was not available, we made the assumption that each node joined the network at the same time as the first edge incident to it appeared. We then divided the evolution time of the network into 30 time frames, each corresponding to a time period during which the network size increased by a constant factor, starting at 0.01*n* up to the final *n*. At each point in time, the balanced edge-based partition, $\left({\hat{E}}_{t}^{*},{\hat{P}}_{t}^{*}\right)$, was calculated and the corresponding observed axiom measures were computed for the elite. More precisely, we computed the observed dominance, robustness and density measures, $dom({\hat{E}}_{t}^{*})$, $rob({\hat{E}}_{t}^{*})$, and $dens({\hat{E}}_{t}^{*})$, respectively, for each time frame *t*.

**Fig 8:**
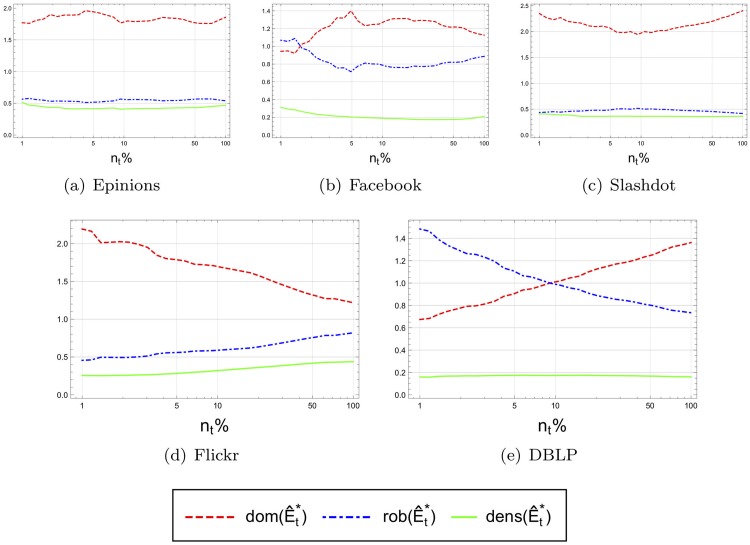
Observed dominance, robustness and density at the balance point of time-varying networks as the networks grows in size over time. To improve the presentation, we plot $\text{dens}({\hat{E}}_{t}^{*})-1$ for the density measure.

Fig 8 presents the performance of these metrics for our five networks with temporal information in their dataset. Note that the balanced edge-based partition (i.e., the rich clubs at the balance point) for the networks ‘Epinions’, ‘Facebook’ and ‘Slashdot’ exhibit relatively stable observed dominance, robustness, and density values, therefore validating the three axioms for the balanced edge-based partition. We call these observed values stable since the trend on non of them indicate they might converge to zero and therefore might violate one of the axioms asymptotically. This implies that the core of the balanced edge-based partition for these network is, asymptotically, of sublinear size. On the other hand, the dominance of the rich club in the ‘Flickr’ network and the robustness of the rich club in ‘DBLP’ decrease with time, indicating that the respective axioms are not valid.

A possible explanation for the ‘Flickr’ case is that this network is composed of a community of professionals and semi-professionals, strongly connected to each other and therefore robust and dense, coexisting side by side with a larger community of occasional users of image storage facilities (the periphery), which are more isolated, and mostly disconnected from the core. Hence abstractly, this case resembles the one depicted in Fig 5(a) which does not satisfy the dominance axiom.

A possible explanation for the ‘DBLP’ case is that the balanced edge-based partition core ${\hat{E}}^{*}$ of this coauthorship network, consisting of the most successful and productive researchers, is dominant and dense since these researchers collaborate extensively both with each other and with individuals from the periphery (such as their students), whereas individuals of the periphery are much less active overall, and each of them tends to work mainly with one (or a few) core member (e.g., the advisor and a few colleagues). At the same time, this core is not robust, since each core member is typically involved more in collaborations with certain individuals from the periphery (such as her students) than with other core members. Hence despite the fact that the ${\hat{E}}^{*}$ is dense, $I({\hat{E}}^{*},{\hat{P}}^{*})$ is larger than both $I({\hat{E}}^{*},{\hat{E}}^{*})$ and $I({\hat{P}}^{*},{\hat{P}}^{*})$, implying that ${\hat{E}}^{*}$ is dominant but not robust. This case abstractly resembles that of Fig 5(b) which does not satisfies the robustness axiom.

The lack of robustness in the ‘DBLP’ network may be partially related to the fact that although this network covers mainly Computer Science and related areas, its scope includes many different and sometimes distant research areas. This implies that the network members belong to different communities, possibly each with its own elite, and the amount of collaborations between members of different communities is relatively small.

**Fig 9:**
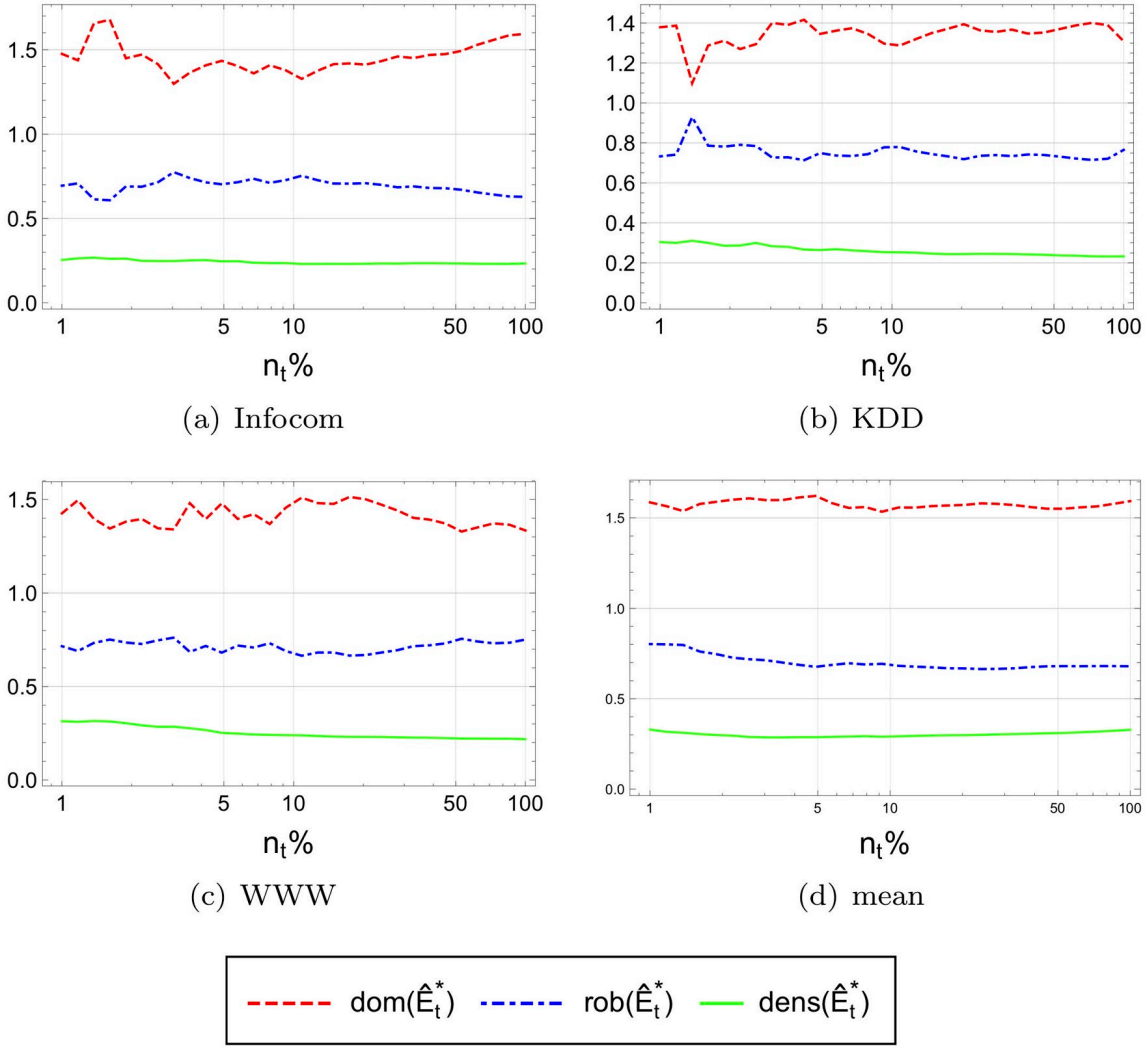
Observed dominance and robustness and density at the balance point of the networks formed by conference communities in DBLP as the networks grows in size over time. To improve the presentation, we plot $\text{dens}({\hat{E}}_{t}^{*})-1$ for the density measure.

To eliminate this factor, we extracted several smaller and more cohesive communities from ‘DBLP’ and re-ran the evaluation on them. We focused on communities built around conferences. More precisely, define the *community* of conference X as all the authors that published at X since its first appearance (using ‘DBLP’ as the source of information). We then considered the timed co-authorship graph between the community members (including publications outside the conference). Fig 9 shows the results for three example conference (’Infocom’, ‘KDD’, and ‘WWW’) and the average over all 13 conferences examined. One can clearly observe that the results are qualitatively different from those obtained for the whole DBLP. The dominance, density and robustness values of these elites at the balance point seem to be more stable. Let us remark, though, that one must be careful interpreting these last results, since the size of the networks corresponding to the individual conferences are orders of magnitude smaller than that of the whole ‘DBLP’ network.

The above results suggests that the axiom are indeed satisfied asymptotically by the balanced edge-based partition which partitions the network at the balance point using the degree order. But, it may be the case that other partitions satisfy the axioms as well. To study this we compared three possible alternative core sizes in addition to the size *bp* at the balance point, a sublinear core of size *n*^0.75^, and two linear size cores, one containing 1% and the second containing 10% of the network nodes.

At each point in time during the network evolution and for each of the four values of *k* mentioned above, the size *k* core was calculated and the observed dominance, robustness and density measures were computed.

**Fig 10:**
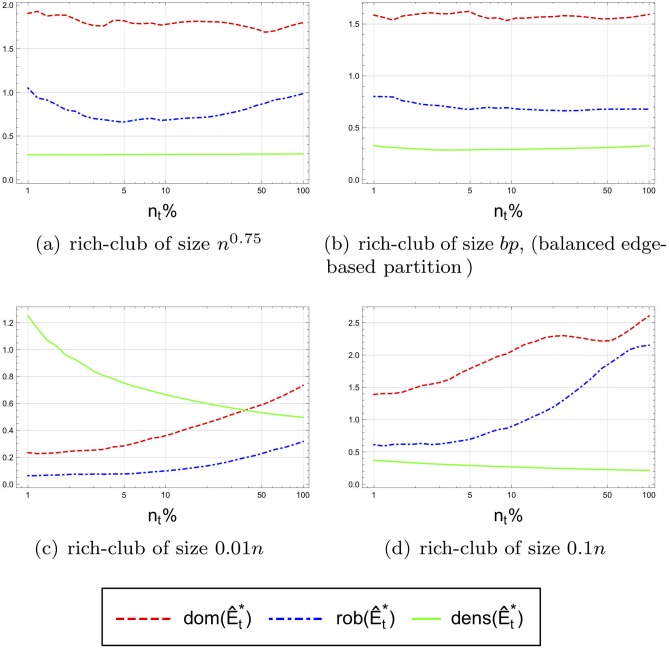
Observed mean of dominance and robustness and density of 5 networks as the networks grows in size over time. To improve the presentation, we plot $\text{dens}({\hat{E}}_{t}^{*})-1$ for the density measure.

Fig 10 shows the results of the average of all 5 networks for the four choices of the size. (The *Y* axis is scaled differently for each figure to focus more on the presentation of the trends of the observed measures and less on their absolute values.) A number of interesting features emerge from this figure. For cores of size *n*^0.75^ and *bp*, the three axioms seem to be satisfied, since the three measures *dom*(), *rob*(), and *dens*() − 1 appear to be bounded from below by a constant (or at least do not diminish to zero). While this may be less surprising for cores of size *n*^0.75^ (since by Theorem 3.3, cores that satisfy the three axioms are expected to be of sublinear size), the observation is more interesting for the balance point *bp*, as it indicates that in our networks, cores at the balance point satisfy all three axioms, namely, qualify as elites, and subsequently are also of sublinear size.

On the other hand, it can easily be seen from Fig 10 that cores of linear size (i.e. 1% and 10% cores) violate axiom (A3), implying that cores of linear size cannot satisfy our three axioms (and hence do not qualify as elites). Nevertheless, these (linear size) cores are still dominant and robust, with these measures increasing over time (which is not the case at the balance point).

Since only for *n* > 10^8^ we have *n*^0.75^ < *n*/100, in our dataset which contains networks with at most few millions nodes it may still hold that an elite of size 1% satisfies all the axioms. Thus it is important to note that these results are only an indication for the asymptotic behaviour of the elite.

### 4.3 Networks with different trends

**Fig 11:**
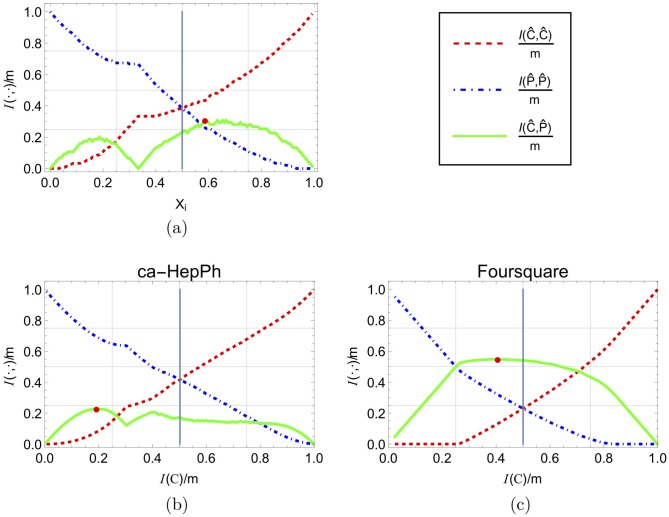
Influence shift diagrams. (a) for the graph model described in 4.3 and for (b) *ca* − *HelPh* and (c) *Foursquare*, two real world networks that exhibit different trends and behavior than most of the networks we examined, and from the average result of all networks. The plots features an edge-normalized influence scale of the *X*-axis with the balance point at *x* = 0.5.

The random configuration model is a general model for random graphs. We observe, however, that there are graph models and graph data sets that do not exhibit the same trends discussed and shown here. An example of such a graph model is as follows. Use the random configuration model to generate two separated graphs, *G*_*a*_ and *G*_*b*_, where each graph *G*_*i*_ has degree sequence **d**^*i*^ of degrees $d_{1}^{i}=d_{2}^{i}=\cdots =d_{n}^{i}=h^{i}$ for constants *h*^*a*^ and *h*^*b*^. Then join these two graphs to form a graph *G* = *G*_*a*_ ∪ *G*_*b*_, without any edge between its two subgraphs. The resulting graph *G* is clearly not a *random* configuration graph, and the resulting influence shift diagram and maximal crossing edges do not have to follow the trends described above. This model represents a network that is constructed by two separated communities. Fig 11(a) shows the influence shift diagram for such graph, with *n*^*a*^ = 50, *n*^*b*^ = 100 and *h*^*a*^ = *h*^*b*^ = 2. It can be seen that the the influence behavior is very different from the configuration model and the real world network discussed in Section 4.

**Empirical results:** Although most of the networks we studied exhibit the trends and properties discussed in the previous sections, not all networks do conform to them. Some of them are shown in Fig 11. Fig 11(b) clearly exhibits a similar trend to the theoretical model described above and shown in Fig 11(a). The influence shift diagram depicted in Fig 11(c) is more similar to the plots in Fig 6, but the maximum number of crossing edges is almost reached when the influence of periphery on itself equals the influence of the crossing edges, significantly before the balance point, and it remains at a high level until the core influence exceeds it.

## 5 Related work

Axioms are extensively used in many scientific fields, such as mathematics, physics and economy. Two examples for the use of an axiomatic paradigm in computer science areas related to ours can be found in [28, 29].

The problems of measuring influence in a network, and identifying useful core-periphery partitions, were also well-studied in the past (see the recent survey [30]). Borgatti and Everett [13] measure the similarity between the adjacency matrix of a graph and the block matrix $\left(\begin{array}{cc} 1 & 1\\ 1 & 0 \end{array}\right)$, following the intuition that social networks have a dense, cohesive core and a sparse, disconnected periphery. an intuition also reflected in the axioms postulated herein.

Methods for identifying core-periphery structures and partitioning networks can be found in [14–16, 31–34]. The recent [35] explains the core-periphery structure as the result of several overlapping communities and proposes a community detection method coping with overlap. None of these works consider the asymptotic size of a core/elite and the possibility that its size is sublinear in the population size.

The term *rich-club coefficient* was introduced in [19] for the density of the nodes of degree *k* or more. This was one of the first papers to observe that the highest degree nodes are well-connected. Xu et al. [36] shows that the rich-club connectivity has a strong influence on the assortativity and transitivity of a network. Weighted and hierarchical versions of the rich-club coefficient have been studied in [37–40]. Mislove et al. [41] defined the *core* of a network to be any (minimal) set of nodes that satisfies the following two properties: (1) the core is essential for ensuring the connectivity of the network, i.e., removing it breaks the remaining nodes into many small, disconnected clusters, and (2) the core is strongly connected with a relatively small diameter. The paper used an approximation technique based on removing increasing numbers of the highest degree nodes (rich clubs) and analyzing the connectivity of the remaining graph. The graphs studied therein have a densely connected core containing 1% to 10% of the highest degree nodes, so removing this core completely disconnects the graph. This provides further evidence that rich clubs are crucial in social networks and satisfy their core properties.

A very different perspective is offered in [42], which studies a *network formation game* where benefits from connections exhibit decreasing returns and decay with network distance. The equilibria of this game form core-periphery structures, which is consistent with our axioms. Another network formation game, where players invest in collecting information, is developed in [43]. The authors show that the economic forces are leading to a robust equilibrium where the majority of individuals to obtain most of the information from a very small (sublinear-size) subset of the group. This observation is termed “The Law of the Few”. These results hold under a more specific set of assumptions, yet they confirm the results derived from our more general axioms.

Motivated by the results about core and periphery *structure* in social networks presented in the current paper, the recent follow-up paper [44] introduced a *complementary* idea regarding computer communication network designs achieving *computational efficiency*. A set of *algorithmic axioms* that resemble a core-periphery structure was proposed as design principles, exploring and promoting the idea that communication networks that satisfy these axioms admit efficient algorithms for a variety of different problems. Indeed, it was established that these algorithmic axioms are necessary and sufficient for a large family of problems to achieve efficient distributed computation (e.g., minimum spanning tree, matrix operations, etc.). Although both the current paper and [44] use the axiomatic approach to explain phenomena, the two papers are radically different in their goals and techniques. Whereas the current paper seeks to explain social networks, [44] is entirely engineering-oriented, seeking to design efficient algorithms on communication networks, and does not address the structure of social networks.

Ideas presented in the current paper were recently used in [45] to study the influence properties of the set of *founders*, the nodes arriving first, in the preferential attachment model of [46] under different model parameters. It turns out that if the number of edges in the model is linear in the number of nodes then networks generated by preferential attachment must have a *linear* size founders set to be dominant, implying that this set will not satisfy our third axiom. On the other hand, if the number of edges in the model is *super-linear* in the number of nodes then the generated networks feature a *sublinear* size founders set that is dominant. This seems to imply that both linear and sublinear cores are possible, depending on the network type.

In the second part of this work we offer a very simple method to identify a core, based on the rich-club notion. Recent work on core-periphery identification proposes a decomposition method using statistical inference [16]. The authors suggest to use an algorithm fitting the stochastic block model with probability *p*_11_ for edges in the core > *p*_12_ for crossing edges > *p*_22_ for edges in the periphery using expectation maximization and belief propagation. This algorithm is then compared to simply using rich-clubs. They show that for very weak and very strong core-periphery structure, the degree-based distinction is the best one can do, followed by derivations on how to compute the size of the rich-club core fitting the *p*_11_ > *p*_12_ > *p*_22_ block model. For core-periphery structures of intermediate strength, their expectation A closely related approach is presented in [47]. To find the boundary of a rich-club core, they examine the escape time it takes a random walker to leave a core. Based on this, they create a decision criterion for the nodes belonging to the core and compare the obtained core size to the total number of nodes in the network. Note that we pursue a slightly different goal. We do not strive for the best partition offering a close fit to a model or a high cohesiveness in the core, but a simple method that lends itself to understanding large graphs better, with a special focus on maximizing the crossing edges.

To the best of our knowledge, the key question of quantifying which degree should be considered as the “stopping criterion”, or in other words, deciding whether a node can still be considered rich, has not received much attention except in [16]. This is one of the main questions to which this article proposes a very simple answer.

## 6 Conclusion

One of the interesting consequences of our results is that, for a core-periphery partition that satisfies the axioms used to model the influence relationships between the elite and the periphery, the core forms an elite of sublinear size in the number of network nodes. In particular, this means that an elite is much smaller than a constant fraction of the network. This is in agreement with the frequent observation that the gap between the very rich and the rest of society is widening as society grows. In turn, this also validates a weaker version of Prices’ law: a *a sublinear fraction of the population (namely the elite) holds a linear fraction of the power*.

To understand better what these axioms mean in practice, we studied a multitude of large real-world social networks. We approximated the elites by *k*-rich-clubs of various sizes *k*. Our findings indicate that in these networks, there are cores exhibiting elite properties such as disproportionate dominance, robustness, and density, as stated by the axioms.

In addition, we presented an analysis of partitioning the network into core and periphery at the balance point for rich-clubs and establish unique properties of this balanced edge-based partition. More precisely, we observed the following on real world data sets. First, at the balance point, or very close to it, the number of crossing edges $E({\hat{C}}_{k},{\hat{P}}_{k})$ reaches its maximum. This is not a mere consequence of the rich-club ordering nor the choice of the balance point, as can be observed by considering the influence shift diagrams we discuss in Section 4.3. Second, the size of the core of the balanced edge-based partition is disproportionally small, confirming the results derived by the axioms.

Some of our findings may have been observed before on an anecdotal level, or may seem obvious; our axioms allow us to quantify the forces at play and compare different core-periphery partitions. For instance, the fact that the *k*-rich-club contains the highest degree nodes does not suffice to account for the behavior of its influence ratios. In particular, inspecting the structure of the *k*-rich-club in *arbitrary* networks reveals that these properties do *not* necessarily hold. For example, when comparing our findings on real-world data to the properties exhibited by the popular Erdös-Renyi class of random graphs, the same behavior cannot be observed there. Furthermore, it is shown in [45] that also in the well-accepted preferential attachment model, that founder cores might not satisfy all our axioms. Thus, it is of a major interest to find evolutionary models in which elites as described here emerge naturally.

We believe that dividing the network to core-periphery structure at the balanced edge-based partition (at the balance point *bp*, using the degree ordering *π*_*deg*_) provides a very simple and useful partition. The properties of this partition lead to partition where the obtained two parts are much simpler for further analysis. First, we obtain a small and dense graph for the core, which is simpler to handle than the entire graph and second, we obtain the periphery, which is a large graph, however sparse, and thus also simpler to handle.

In summary, our results advance the theoretical understanding of the elite of social structures, and moreover, may also help to improve infrastructure and algorithms targeted at online social networks [44], e.g., to organize institutions better, or identify sources of power in social networks in general.

## A Appendix

### A.1 Proofs

The proofs of the statements in Section 3 are presented in this part.

#### A.1.1 Axiom independence

We prove that the axioms are independent, by constructing three examples of families of *n*-vertex (undirected, unweighted) networks and core-periphery partitions. Under the edge-based influence model, each of these partitions satisfies two of the axioms, but violates the third. The existence of an interpretation exhibiting such behavior implies axiom independence (Theorem 3.1).

*Proof of Theorem 3.1*.

The first network and partition (Fig 5(a)), depict a core that is robust and dense but whose dominance tends to zero as the network size *n* grows to infinity. The second example (Fig 5(b)) describes a core that is dominant and dense but whose robustness tends to zero as the network grows. The last example (Fig 5(c)) describes a core that is dominant and robust but whose density tends to one as the network size *n* grows to infinity, i.e., the average degree of core members and periphery members is almost the same.

#### A.1.2 Balance of power

In this part we show that for any elite that satisfies Axioms (A1) and (A2) and the compactness property (P5), the overall influence of the elite, $I(\hat{E})$, is (nearly) equal to the overall influence of the periphery and thus is close to the balance point (Theorem 3.2).

*Proof of Theorem 3.2*.

To prove this theorem we need a simple fact and two lemmas. Recall that the number of edges in the graph is *m*. As $\left(\hat{E},\hat{P}\right)$ forms a *partition* of the network vertices, we have

**Fact A.1.** $I(\hat{E},\hat{E})+I(\hat{E},\hat{P})+I(\hat{P},\hat{E})+I(\hat{P},\hat{P})=I_{tot}$.

**Lemma A.2.** *In a symmetric system, if* $\left(\hat{E},\hat{P}\right)$ *satisfies the dominance and robustness axioms (A1)-(A2), then for some constants c*_1_, *c*_2_ > 0,

1. $I(\hat{E},\hat{E})\;\geq \;c_{1}\cdot I(\hat{P},\hat{P})$,
2. $I(\hat{E},\hat{E})\;\geq \;c_{2}\cdot I_{tot}$.

*Proof*. By the symmetry property (P2) and the two axioms, we have that

$$\begin{array}{c} I\left(\hat{E},\hat{E}\right)\;\geq \;c_{r}\cdot I\left(\hat{P},\hat{E}\right)\;=\;c_{r}\cdot I\left(\hat{E},\hat{P}\right)\;\geq \;c_{r}c_{d}\cdot I\left(\hat{P},\hat{P}\right), \end{array}$$

implying the first claim with *c*_1_ = *c*_*r*_*c*_*d*_.

Also, by the symmetry property (P2) and Fact A.1, combined with the two axioms,

$$\begin{array}{c} I_{tot}\leq (1+\frac{2}{c_{r}}+\frac{1}{c_{r}c_{D}})I\left(\hat{E},\hat{E}\right). \end{array}$$

Hence $I(\hat{E},\hat{E})\geq c_{2}I_{tot}$ for *c*_2_ = (1 + 2/*c*_*r*_ + 1/(*c*_*r*_*c*_*D*_))^−1^. The second claim follows.

Let us next consider the implications of the compactness property (P5).

**Lemma A.3.** *If the elite* $\hat{E}$ *satisfies axioms (A1), (A2), (A3) and also the compactness property (P5), then* $I(\hat{E},\hat{P})=\Omega (I_{tot})$.

*Proof*. By Thm. 3.3, $|\hat{E}|\ll n$, which implies that $|\hat{P}|\geq n/2$. By the self-influence property (P3) it follows that $I(\hat{P},\hat{P})\geq n/2$. Combining this with Axiom (A1), we get that $I(\hat{E},\hat{P})\geq c_{d}\cdot n/2$. Hence if, say, $I_{tot}\leq \frac{2(1+c_{r})}{c_{2}}\cdot n$, for the constant *c*_2_ of Lemma A.2, then the lemma holds trivially. Hence herafter we consider networks where

$$I_{tot}>\frac{2(1+c_{r})}{c_{2}}\cdot n.$$ (3)

Consider an elite $\hat{E}$ that satisfies the compactness property (P5), i.e., it is a minimal set of vertices satisfying Axioms (A1) and (A2). This implies that for every vertex $v\in \hat{E}$, moving *v* from $\hat{E}$ to $\hat{P}$ violates either (A1) or (A2).

Let us first consider the case where there exists a vertex $v\in \hat{E}$ whose movement from $\hat{E}$ to $\hat{P}$ violates the robustness Axiom (A2). In other words, letting ${\hat{E}}^{\prime }=\hat{E}\backslash \{v\}$ and ${\hat{P}}^{\prime }=\hat{P}\cup \{v\}$, we have, by the additivity property (P1), that

$$\begin{array}{c} I\left({\hat{E}}^{\prime },{\hat{E}}^{\prime }\right)\;<\;c_{r}\cdot I\left({\hat{E}}^{\prime },{\hat{P}}^{\prime }\right)\;, \end{array}$$

or

$$\begin{array}{c} I\left(\hat{E},\hat{E}\right)-I\left(\hat{E},v\right)\;<\;c_{r}\cdot \left(I\left(\hat{P},\hat{E}\right)+\left(I\left(\hat{E},v\right)-1\right)-I\left(\hat{P},v\right)\right). \end{array}$$

Rearranging, we get that

$$\begin{array}{c} I\left(\hat{E},\hat{P}\right)>\frac{I\left(\hat{E},\hat{E}\right)-\left(1+c_{r}\right)I\left(\hat{E},v\right)}{c_{r}}>\frac{I\left(\hat{E},\hat{E}\right)-\left(1+c_{r}\right)n}{c_{r}}\;, \end{array}$$

where the last inequality relies on Property (P3). Applying Lemma A.2 (2) and Eq (3) we get that

$$\begin{array}{c} I\left(\hat{E},\hat{P}\right)\;>\;\frac{1}{c_{r}}\cdot (c_{2}I_{tot}-\left(1+c_{r}\right)n)\;>\;\frac{c_{2}}{2c_{r}}\cdot I_{tot}. \end{array}$$

Next, let us consider the complementary case, where for every vertex $v\in \hat{E}$, moving *v* from $\hat{E}$ to $\hat{P}$ does not violate the robustness Axiom (A2). In this case, for every vertex $v\in \hat{E}$, moving *v* from $\hat{E}$ to $\hat{P}$ necessarily violates the dominance Axiom (A1). This means that for every vertex $v\in \hat{E}$, again letting ${\hat{E}}^{\prime }=\hat{E}\backslash \{v\}$ and ${\hat{P}}^{\prime }=\hat{P}\cup \{v\}$,

$$\begin{array}{c} I\left({\hat{E}}^{\prime },{\hat{P}}^{\prime }\right)\;<\;c_{d}\cdot I\left({\hat{P}}^{\prime },{\hat{P}}^{\prime }\right)\;, \end{array}$$

or

$$\begin{array}{c} I\left(\hat{E},\hat{P}\right)+\left(I\left(\hat{E},v\right)-1\right)-I\left(\hat{P},v\right)\;<\;c_{d}\cdot \left(I\left(\hat{P},\hat{P}\right)+\left(I\left(\hat{P},v\right)+1\right)\right). \end{array}$$

On the other hand we have by Axiom (A1) that

$$\begin{array}{c} I\left(\hat{E},\hat{P}\right)\;\geq \;c_{d}\cdot I\left(\hat{P},\hat{P}\right). \end{array}$$

Adding up these two inequalities and simplifying,

$$\begin{array}{c} I\left(\hat{E},v\right)<\left(1+c_{d}\right)\cdot I\left(\hat{P},v\right)+2<2I\left(\hat{P},v\right)+2. \end{array}$$

Summing over all $v\in \hat{E}$, $2I(\hat{E},\hat{E})<2I(\hat{E},\hat{P})+2|\hat{E}|$, so

$$\begin{array}{c} I\left(\hat{E},\hat{P}\right)\;>\;I\left(\hat{E},\hat{E}\right)-\left|\hat{E}\right|\geq c_{2}I_{tot}-n\;\geq \;\frac{c_{2}}{2}\cdot I_{tot}. \end{array}$$

Theorem 3.2 now follows by the above lemmas.

#### A.1.3 Elite size

We now consider the size of the elite and prove Theorem 3.3, showing that elite-centered social networks feature an elite of sublinear size, i.e.,

$$c_{1}\cdot n^{\frac{\delta (V)}{\delta (\hat{E})}}\leq \left|\hat{E}\right|\leq n^{\frac{1}{1+c_{c}}}.$$ (4)

*Proof of Theorem 3.3*.

To prove this theorem we first observe that by Lemma A.2(2) and Eq (1) we immediatelly have the following.

**Lemma A.4.** *In a symmetric system, if* $\left(\hat{E},\hat{P}\right)$ *satisfies the dominance and robustness axioms (A1) and (A2), then* $c_{1}\cdot I_{tot}\;\leq \;I(\hat{E})\;\leq \;I_{tot}$ *for some constant c*_1_ > 0.

Recall that by the definition of *δ*(*X*) we have

$$\begin{array}{ccc} I_{tot} & = & n^{\delta (V)}, \end{array}$$ (5)

$$\begin{array}{ccc} I(\hat{E}) & = & {\left|\hat{E}\right|}^{\delta (\hat{E})}. \end{array}$$ (6)

Using Lemma A.4 we get that

$$\begin{array}{c} c_{1}\cdot n^{\delta (V)}\;\leq \;{\left|\hat{E}\right|}^{\delta (\hat{E})}\;\leq \;n^{\delta (V)}\;=\;I_{tot}\;. \end{array}$$

Raising by the power of $1/\delta (\hat{E})$ we get that

$$c_{1}\cdot n^{\frac{\delta (V)}{\delta (\hat{E})}}\leq c_{1}^{\frac{1}{\delta (\hat{E})}}\cdot n^{\frac{\delta (V)}{\delta (\hat{E})}}\leq \left|\hat{E}\right|\leq n^{\frac{\delta (V)}{\delta (\hat{E})}}.$$ (7)

The left hand side of Eq (4) follows directly from the left hand side of Eq (7). The upper bound of Eq (4) follows from the right hand side of Eq (7) by Axiom (A3), which implies that $\delta (V)/\delta (\hat{E})\leq 1/(1+c_{c})$.

Axiom (A3) is crucial for determining the core size, since for every core-periphery partition $\left(\hat{E},\hat{P}\right)$ that satisfies Axioms (A1) and (A2) but does not satisfy Axiom (A3), the core size is linear in the network size (Lemma 3.4).

*Proof of Lemma 3.4*.

By the premise of the lemma, $\left|\hat{E}|\geq I(\hat{E})\cdot n/(c\cdot I_{tot}\right)$. By Lemma A.4, $I(\hat{E})/I_{tot}\geq c_{1}$. Combining, we have $|\hat{E}|\geq (c_{1}/c)n$.

On the other hand, there are elite-centered asymmetric or unbounded networks with an elite of constant size (Lemma 3.5).

*Proof of Lemma 3.5*.

For asymmetric but bounded networks, a simple example is the *star* graph, under the edge-based influence model, where a single vertex (the center, forming the elite) has a directed edge to each of the periphery vertices (with no incoming edges). Assuming the edge-based influence model, the star center clearly dominates the periphery, and it is robust and dense.

Next consider symmetric but unbounded networks. Again using the edge-based influence model, consider a tree network with *n* = 2*k* vertices, so *m* = 4*k* − 1 (including self-loops). The weight of each self-loop is 1, totaling 2*k*. Now the tree is constructed from two stars, with uniform edge weights of 1/2, plus an edge of weight *k* connecting the two centers of the stars. It is easy to check that the sum of the edge weights is *m*. The elite consisting of the two star centers satisfies all three axioms.

For symmetric bounded networks again using the edge-based influence model, we prove a tighter square root lower bound on the elite size (Theorem 3.6).

First note that as a result of the additivity property (P1) and the bounded influence property (P4), the following holds.

**Fact A.5.** *For every X, Y* ⊆ *V*,

$$\begin{array}{c} I\left(X,Y\right)\;=\;\sum_{x\in X}\sum_{y\in Y}I\left(x,y\right)\;\leq \;c_{b}\cdot \left|X\right|\cdot |Y|. \end{array}$$

*Proof of Theorem 3.6*.

By Lemma A.2(2), $I(\hat{E},\hat{E})\;\geq \;c_{2}\cdot I_{tot}$ for some constant *c*_2_ > 0. By Fact A.5, $I(\hat{E},\hat{E})\leq c_{b}\cdot |\hat{E}{|}^{2}$, implying that $|\hat{E}|\geq \sqrt{I(\hat{E},\hat{E})/c_{b}}$. Combining these two inequalities, the theorem follows.

## B The shape of the influence shift diagram in the configuration model

In this section we study the shape of the Influence Shift Diagram in a very general random graph model known as the *configuration model* [21]. We study the behavior of $I({\hat{C}}_{k},{\hat{P}}_{k})$, $I({\hat{C}}_{k},{\hat{C}}_{k})$ and $I({\hat{P}}_{k},{\hat{P}}_{k})$ for *k* = 1, …, *n* under this model, and prove formally some of the properties discussed (and observed empirically) in this paper for any order *π*.

Let **d** = *d*_1_, *d*_2_, …, *d*_*n*_, where 1 ≤ *d*_*i*_ ≤ *n* − 1 be a positive degree sequence (it is not necessary that *d*_1_ ≥ *d*_2_ ≥ … ≥ *d*_*n*_). A *random configuration (multi)graph* $\bar{G}(n,d)$ is constructed in the following way over the set of nodes [*n*]. Let *W* = [2*m*] be the set of *configuration points* where $m=\sum_{1}^{n}d_{i}/2$ is the number of edges. Define the ranges $W_{j}=[1+\sum_{1}^{j-1}d_{i}\;,\;\sum_{1}^{j}d_{i}]$ for *j* ∈ [*n*]. Given a pairing *F* (i.e., a partition of *W* into *m* pairs) we obtain a (multi) graph *G*_*F*_, with node set [*n*] and for each pair (*u*, *v*) ∈ *F* we add an edge (*i*, *j*) to *G*_*F*_ where *u* ∈ *W*_*i*_ and *v* ∈ *W*_*j*_ (including self loops). Choosing *F* uniformly from all possible pairings of *W* we obtain a random (multi-)graph $\bar{G}(n,d)$.

First we study the behavior of the influence shift diagram for random configuration graphs. For *U* ⊆ {1, …, *n*}, let

$$\begin{array}{c} p\left(U\right)=\sum_{i\in U}\frac{d_{i}}{2m}. \end{array}$$

For 1 ≤ *k* ≤ *n*, consider the core-periphery partition ${\hat{C}}_{k}=\{1,\ldots ,k\}$ and ${\hat{P}}_{k}=\{k+1,\ldots ,n\}$, and set $Z_{k}=p({\hat{C}}_{k})$.

**Theorem B.1.** *Let* $\bar{G}(n,d)$ *be a random configuration graph of a given positive degree sequence* **d.** *Then*

1. $E[I({\hat{C}}_{k},{\hat{C}}_{k})/m]=Z_{k}^{2}$,
2. $E[I({\hat{P}}_{k},{\hat{P}}_{k})/m]=(1-Z_{k}{)}^{2}$,
3. $E[I({\hat{C}}_{k},{\hat{P}}_{k})/m]=2Z_{k}(1-Z_{k})$.

*Proof*. In the configuration model, the process of generating an edge (pairing) is to choose one end of the edge, out of the 2*m* configuration points, and then the other end. Given one end of the edge already picked to be node *i*, the probability of choosing *j* is *p*(*j*) = *d*_*j*_/(2*m*). Given a set of nodes *U* ⊂ *V*, the probability of choosing one end of an edge to be in *U* is *p*(*U*). For two (not necessarily distinct) sets of nodes *U*_1_, *U*_2_ ∈ *V*, the probability of an edge (*i*, *j*) where *i* ∈ *U*_1_ and *j* ∈ *U*_2_ is *p*(*U*_1_) ⋅ *p*(*U*_2_). (There is a slight inaccuracy in this calculation, since in the situation where *U*_1_ and *U*_2_ are not distinct, after choosing one end of the edge from *U*_1_, which may be also in *U*_2_, the probability of choosing the second end at *U*_2_ becomes smaller. However, this effect is negligible when the sets of nodes are relatively large.) The expected number of edges between *U*_1_ and *U*_2_ is $E[I(U_{1},U_{2})]=m\cdot p(U_{1})\cdot p(U_{2})$. Given a core-periphery with a partition index *k*, the expected number of edges of each component satisfies

$E[I({\hat{C}}_{k},{\hat{C}}_{k})]=m\cdot p({\hat{C}}_{k})\cdot p({\hat{C}}_{k})=m\cdot Z_{k}^{2}$,
$E[I({\hat{P}}_{k},{\hat{P}}_{k})]=m\cdot p({\hat{P}}_{k}{)}^{2}=m\cdot (1-Z_{k}{)}^{2}$,
$E[I({\hat{C}}_{k},{\hat{P}}_{k})]=m\cdot 2p({\hat{C}}_{k})\cdot p({\hat{P}}_{k})=m\cdot 2Z_{k}(1-Z_{k})$.

Fig 7(c) shows the influence shift diagram and the expected behavior of $I({\hat{C}}_{k},{\hat{P}}_{k})$, $I({\hat{C}}_{k},{\hat{C}}_{k})$ and $I({\hat{P}}_{k},{\hat{P}}_{k})$ for random configuration graphs.

## C Maximal number of crossing edges

One of the most striking properties of partitioning the network at the balance point is that in most networks, the maximal number of crossing edges occurs at the balance point or near it. We show this empirically and also prove it theoretically for the random configuration graph model. This is not a trivial phenomenon that is expected from the ordering, *π*_*deg*_, (or another ordering). It is also not the result of the fact that we focus on the point of equality between the powers of the core and periphery. Section 4.3 discusses and shows theoretical and real examples where this property does not hold. In terms of dividing the network into the most simple parts, this means that the balanced edge-based partition ‘discards’ the maximal number of edges, thus minimizing the number of meaningful edges left in the induced subgraphs of edges within the same part.

We first prove this observation theoretically for the random configuration graphs model (see Appendix B for definitions). Define the *balance point* *bp*(**d**) of the degree sequence **d** as

$$\begin{array}{c} bp\left(d\right)\;=\;\arg\min_{j}|\sum_{i=1}^{j}d_{i}/\left(2m\right)-\frac{1}{2}|\;=\;\arg\min_{j}|Z_{j}-\frac{1}{2}|. \end{array}$$

Recall that ${\hat{C}}_{k}=\{1,\ldots ,k\}$ and ${\hat{P}}_{k}=\{k+1,\ldots ,n\}$. We now prove that in expectation $E[I({\hat{C}}_{k},{\hat{P}}_{k})]$ is maximized at the balance point.

**Theorem C.1.** *Let* $\bar{G}(n,d)$ *be a random configuration graph of a given positive degree sequence* **d** *and let bp* = *bp*(**d**) *be the balance point of* **d.** *Then*

$$\begin{array}{cc} \forall k & E[I({\hat{C}}_{k},{\hat{P}}_{k})]\leq E[I({\hat{C}}_{bp},{\hat{P}}_{bp})] \end{array}$$

*Proof*. As shown in Theorem B.1, $E[I({\hat{C}}_{k},{\hat{P}}_{k})]=m\cdot 2Z_{i}(1-Z_{i})$. This function reaches its maximum at $Z_{i}=\frac{1}{2}$, which is exactly the balance point, *i* = *bp*(**d**).

**Empirical results:** In Fig 6 we can see that $I({\hat{C}}_{k},{\hat{P}}_{k})$, the number of crossing edges between the core and periphery, achieves its maximum at (or very close to) the balance point. Most of the networks that we studied exhibit this behavior. We show in Fig 6 example networks that exhibit this behavior, but also most of the networks that are not presented here show the same behavior. Fig 7(b) shows the average of all 32 networks and as we can see $I({\hat{C}}_{k},{\hat{P}}_{k})$ achieves its maximum very close to the balance point in this figure too. We must stress that although most of the networks we studied exhibit the same trend as discussed, not all networks do conform to this trend (see Section 4.3). Note that the average graph given in Fig 7(b) does not include these networks.

## Supporting information

S1 Supporting Information Elites in social networks—Datasets information. (PDF)
