# Supplementary material for: Elites in social networks: An axiomatic approach to power balance and Price’s square root law
Source: PLoS One. 2018 Oct 24;13(10):e0205820. doi: 10.1371/journal.pone.0205820 (PMC6200247; doi:10.1371/journal.pone.0205820)
Supplement: S1 Supporting Information — (PDF) [file pone.0205820.s001.pdf]

S1 Supporting Information

Elites in Social Networks - Datasets information

June 4, 2018

1 Networks' Data

No.	Data	Repository	n	m	Average Degree	Max Degree	Undirected Directed	Description
1	Blog [3][50]	ASU	97884	1668647	17.0	27849	U	Social blog directory which manages bloggers their blog
2	Buzznet [4][50]	ASU	101163	2763066	27.3	64289	U	Photo, journal, and video-sharing social media network
3	Delicious [10][50]	ASU	536408	1366136	2.5	3216	U	Social bookmarking web service for storing sharing and discovering web bookmarks
4	Digg [11][50]	ASU	771229	5907413	7.7	17643	U	Social news website
5	Douban [13][50]	ASU	154908	327162	2.1	287	U	Chinese website providing user review and recommendation services for movies books and music

Continued on next page

Table 1 – continued from previous page

No.	Data	Repository	n	m	Average Degree	Max Degree	Undirected Directed	Description
6	Flixster [17][50]	ASU	2523386	7918801	3.1	1474	U	Social movie site allowing users to share movie ratings discover new movies and meet others with similar movie taste
7	Foursquare [18][50]	ASU	639014	3214986	5.0	106218	U	Location-based social networking website software for mobile devices. This service is available to users with GPS enabled mobile devices such as iPhones and Blackberries
8	Friendster [19][50]	ASU	5689498	14067887	2.5	4423	U	Social networking website.The service allows users to contact other members maintain those contacts and share online content and media with those contacts.

Continued on next page

Table 1 – continued from previous page

No.	Data	Repository	n	m	Average Degree	Max Degree	Undirected Directed	Description
9	Hyves [21][50]	ASU	1402673	2777419	2.0	31883	U	The most popular social networking site in the Netherlands with mainly Dutch visitors and members and competes in this country with sites such as Facebook and MySpace.
10	LastFm [22][50]	ASU	1191812	4519340	3.8	5150	U	Music website founded in the United Kingdom in 2002. It has claimed over 40 million active users based in more than 190 countries.
11	Livemocha [24][50]	ASU	104103	2193083	21.1	2980	U	The world's largest online language learning community offering free and paid online language courses in 35 languages to more than 6 million members from over 200 countries around the world.
								Continued on next page

Table 1 – continued from previous page

No.	Data	Repository	n	m	Average Degree	Max Degree	Undirected Directed	Description
12	Twitter [30][50]	ASU	11316811	63555749	5.6	564795	D	Social news website. It can be viewed as a hybrid of email instant messaging and sms messaging all rolled into one neat and simple package. It's a new and easy way to discover the latest news related to subjects you care about.
13	Academia [1][36][37]	BGU	200169	1022441	5.1	10693	D	Platform for academics to share research papers.
14	AnyBeat [2][44]	BGU	12645	49132	3.9	4800	D	Online community. A public gathering place where you can interact with people from around your neighborhood or across the world
15	GooglePlus [20][37]	BGU	211186	1141650	5.4	1790	D	Google+ is a social networking service and website offered by Google

Continued on next page

Table 1 – continued from previous page

No.	Data	Repository	n	m	Average Degree	Max Degree	Undirected Directed	Description
16	TheMarkerCafe [29][36][37]	BGU	69413	1644843	23.7	8930	U	Israeli online social network site that allows its member to connect and interact
17	Catster [8][38]	Konect	149684	5448196	36.4	80634	U	This Network contains friendships between users of the website catster.com.
18	DBLP [9][38][43]	Konect	1103412	4225686	3.8	1189	U	Collaboration graph of authors of scientific papers from DBLP computer science bibliography
19	Dogster [12][38]	Konect	426816	8543548	20.0	46503	U	This Network contains friendships between users of the website dogster.com.
20	Epinions [14][38][45]	Konect	131580	711210	5.4	3558	D	Trust and distrust network of Epinions, an online product rating site
21	Facebook [15][38][49]	Konect	63731	817090	12.8	1098	U	Friendship data of facebook users

Continued on next page

Table 1 – continued from previous page

No.	Data	Repository	n	m	Average Degree	Max Degree	Undirected Directed	Description
22	Flickr [16][38][47]	Konect	2302925	22838276	9.9	27937	D	Social network of users and their friendship connections
23	Slashdot [28][38][33]	Konect	51083	116573	2.3	2915	D	Reply network of technology website
24	YouTube [32][38][46]	Konect	3223589	9376594	2.9	91751	U	Video-sharing website on which users can upload share and view videos. Social network of users and their friendship connections
25	ca-AstroPh [5][41]	SNAP	18771	198050	10.6	504	U	Arxiv ASTRO-PH (Astro Physics) collaboration network
26	ca-CondMat [6][41]	SNAP	23133	93439	4.0	279	U	Arxiv COND-MAT (Condense Matter Physics) collaboration network
27	ca-HepPh [7][41]	SNAP	12006	118489	9.9	491	U	Arxiv HEP-PH (High Energy Physics - Phenomenology) collaboration network

Continued on next page

Table 1 – continued from previous page

No.	Data	Repository	n	m	Average Degree	Max Degree	Undirected Directed	Description
28	LiveJournal [23][34][42]	SNAP	4846609	42851237	8.8	20333	D	Virtual community where Internet users can keep a blog journal or diary
29	loc-brightkite [25][35]	SNAP	58228	214078	3.7	1134	U	Location-based social networking service provider where users shared their locations by checking-in.
30	loc-gowalla [26][35]	SNAP	196591	950327	4.8	14730	U	Location-based social networking website where users share their locations by checking-in.
31	Pokec [27][48]	SNAP	1632803	22301964	13.7	14854	D	Pokec is the most popular on-line social network in Slovakia.
32	wiki-talk [31][40][39]	SNAP	2394385	4659565	1.9	100029	D	Wikipedia's registered users talk pages. A directed edge from node i to node j represents that user i at least once edited a talk page of user j.

Table 1: List of All Networks

Network	Duration (months)
DBLP	913
Epinions	31
Facebook	29
Flickr	7
Slashdot	33

Table 2: Duration of Networks with Available Time Information

2 Symmetry Point Data: $\hat{C} = \hat{C}_{sp}$

No.	Network	$\lvert\hat{C}\rvert$	$\lvert\hat{P}\rvert$	$\frac{\lvert\hat{C}\rvert}{n}$	x $(\lvert\hat{C}\rvert = n^x)$	$I(\hat{C},\hat{C})$	$I(\hat{P},\hat{P})$	$I(\hat{C},\hat{P})$
1	Blog	1234	96650	0.01	0.62	204490	204391	1259766
2	Buzznet	3472	97691	0.03	0.71	439028	438799	1885239
3	Delicious	25985	510423	0.05	0.77	295908	295894	774334
4	Digg	8271	762958	0.01	0.67	1493484	1493313	2920616
5	Douban	5562	149346	0.04	0.72	50380	50344	226438
6	Flixster	35783	2487603	0.01	0.71	1056551	1056538	5805712
7	Foursquare	19691	619323	0.03	0.74	738906	738877	1737203
8	Friendster	57702	5631796	0.01	0.70	891165	891140	12285582
9	Hyves	83294	1319379	0.06	0.80	335630	335630	2106159
10	LastFm	30474	1161338	0.03	0.74	745347	745299	3028694
11	Livemocha	6268	97835	0.06	0.76	480403	480292	1232388
12	Twitter	20805	11296006	0.00	0.61	6113208	6112570	51329971
13	Academia	21012	179157	0.10	0.82	280301	280296	461844
14	AnyBeat	377	12268	0.03	0.63	9607	9606	29919
15	GooglePlus	7515	203671	0.04	0.73	392844	392816	355990
16	TheMarkerCafe	2568	66845	0.04	0.70	357328	357263	930252
17	Catster	2814	146870	0.02	0.67	890873	890809	3666514
18	DBLP	121853	981559	0.11	0.84	1256888	1256886	1711912
19	Dogster	11765	415051	0.03	0.72	1732323	1732213	5079012
20	Epinions	2554	129026	0.02	0.67	184495	184494	342221
21	Facebook	7255	56476	0.11	0.80	261329	261329	294432
22	Flickr	21165	2281760	0.01	0.68	7093818	7093615	8650843
23	Slashdot	2389	48694	0.05	0.72	26489	26481	63603
24	YouTube	78229	3145360	0.02	0.75	2254777	2254753	4867064
25	ca-AstroPh	2359	16412	0.13	0.79	62404	62389	73257
26	ca-CondMat	3652	19481	0.16	0.82	27424	27420	38595
27	ca-HepPh	672	11334	0.06	0.69	49083	49036	20370
28	LiveJournal	348967	4497642	0.07	0.83	14108588	14108536	14634113
29	loc-brightkite	3898	54330	0.07	0.75	63154	63146	87778
30	loc-gowalla	13392	183199	0.07	0.78	293793	293780	362754

Continued on next page

Table 3 – continued from previous page

No.	Network	$\lvert\hat{C}\rvert$	$\lvert\hat{P}\rvert$	$\frac{\lvert\hat{C}\rvert}{n}$	x $(\lvert\hat{C}\rvert = n^x)$	$I(\hat{C},\hat{C})$	$I(\hat{P},\hat{P})$	$I(\hat{C},\hat{P})$
31	Pokec	213044	1419759	0.13	0.86	6312132	6312130	9677702
32	wiki-talk	5832	2388553	0.00	0.59	405723	405709	3848133

Table 3: Netwroks' Symmetry Point

3 Influence Shift Diagrams

In this section we plot the influence shift diagrams. Each plot shows the influence $I(\hat{C}, \hat{C})/m$, $I(\hat{P}, \hat{P})/m$ and $I(\hat{C}, \hat{P})/m$ for the k-rich-club in networks in the dataset. A point y on the Y axis indicates the fraction $I(\cdot, \cdot)/m$, where m is the total number of edges.

3.1 $x = \frac{I(\hat{C})}{m}$

In the figures at this section a point x (in [0,1]) represents $\frac{I(\hat{C})}{m}$.

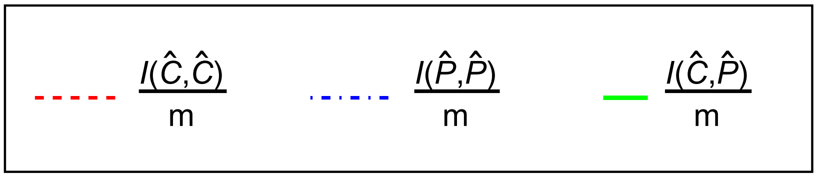

(a)

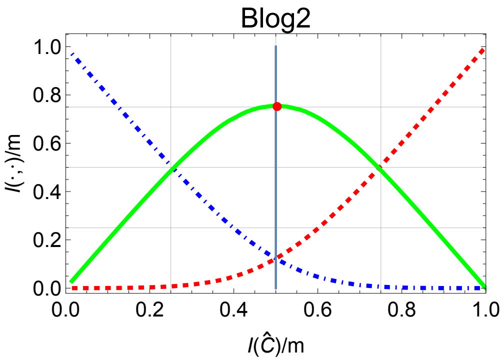

(b) Blog

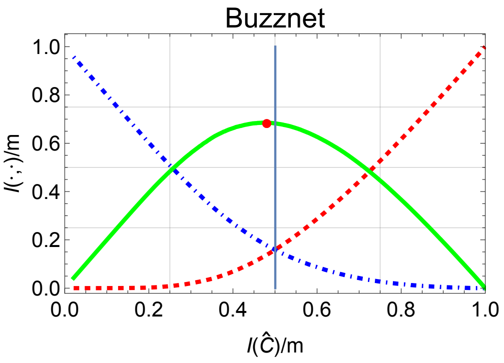

(c) Buzznet

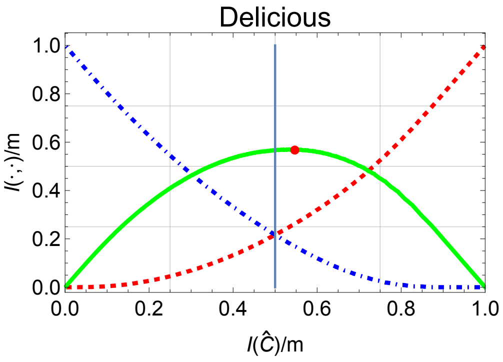

(d) Delicious

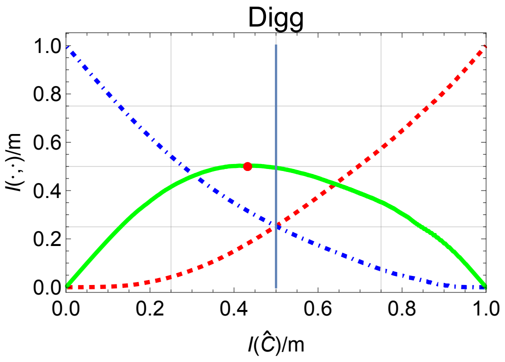

(e) Digg

Figure 1: The influence shift diagram for Blog, Buzznet, Delicious and Digg

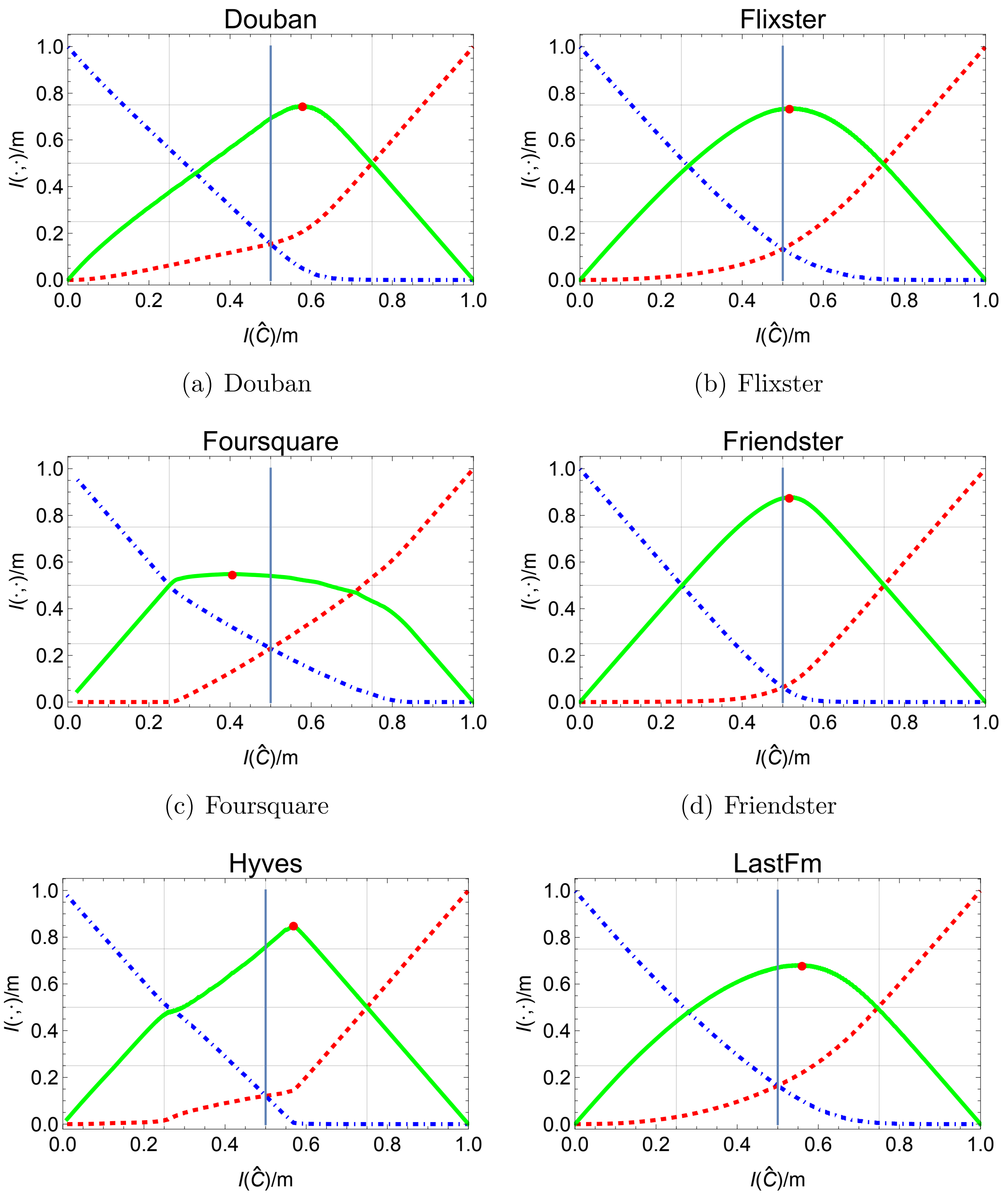

(a) Douban (b) Flixster

(c) Foursquare (d) Friendster

(e) Hyves (f) LastFm

Figure 2: Influence shift diagram for Douban and Flixster, Foursquare, Friendster, Hyves and LastFm

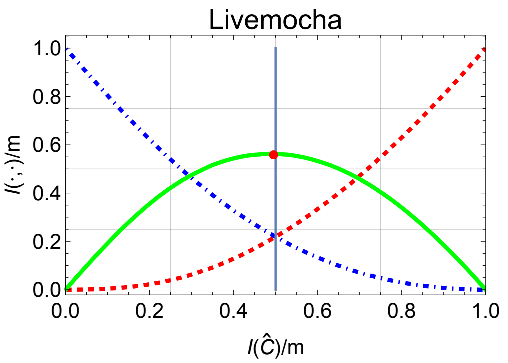

(a) Livemocha

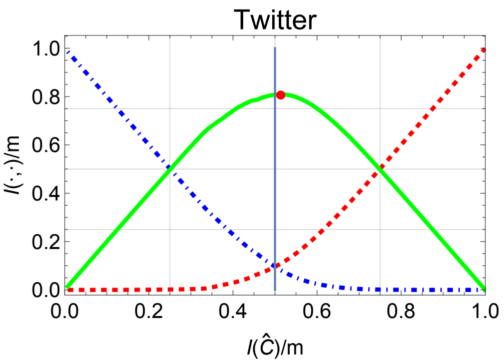

(b) Twitter

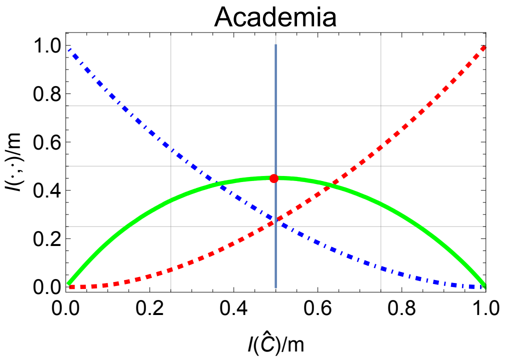

(c) Academia

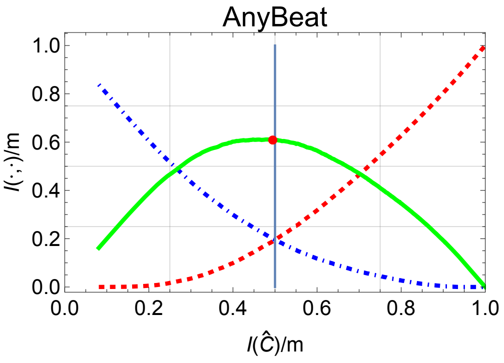

(d) AnyBeat

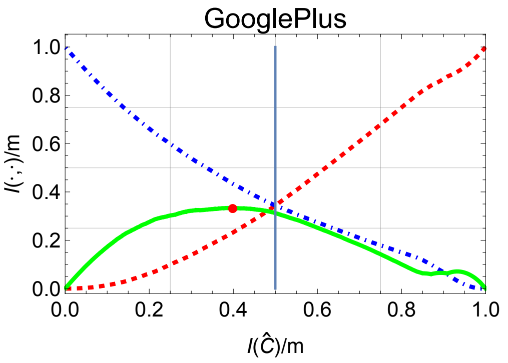

(e) GooglePlus

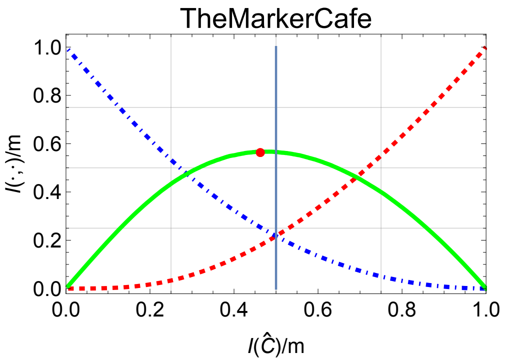

(f) TheMarkerCafe

Figure 3: Influence shift diagram for Livemocha, Twitter, Academia, AnyBeat, GooglePlus and TheMarkerCafe

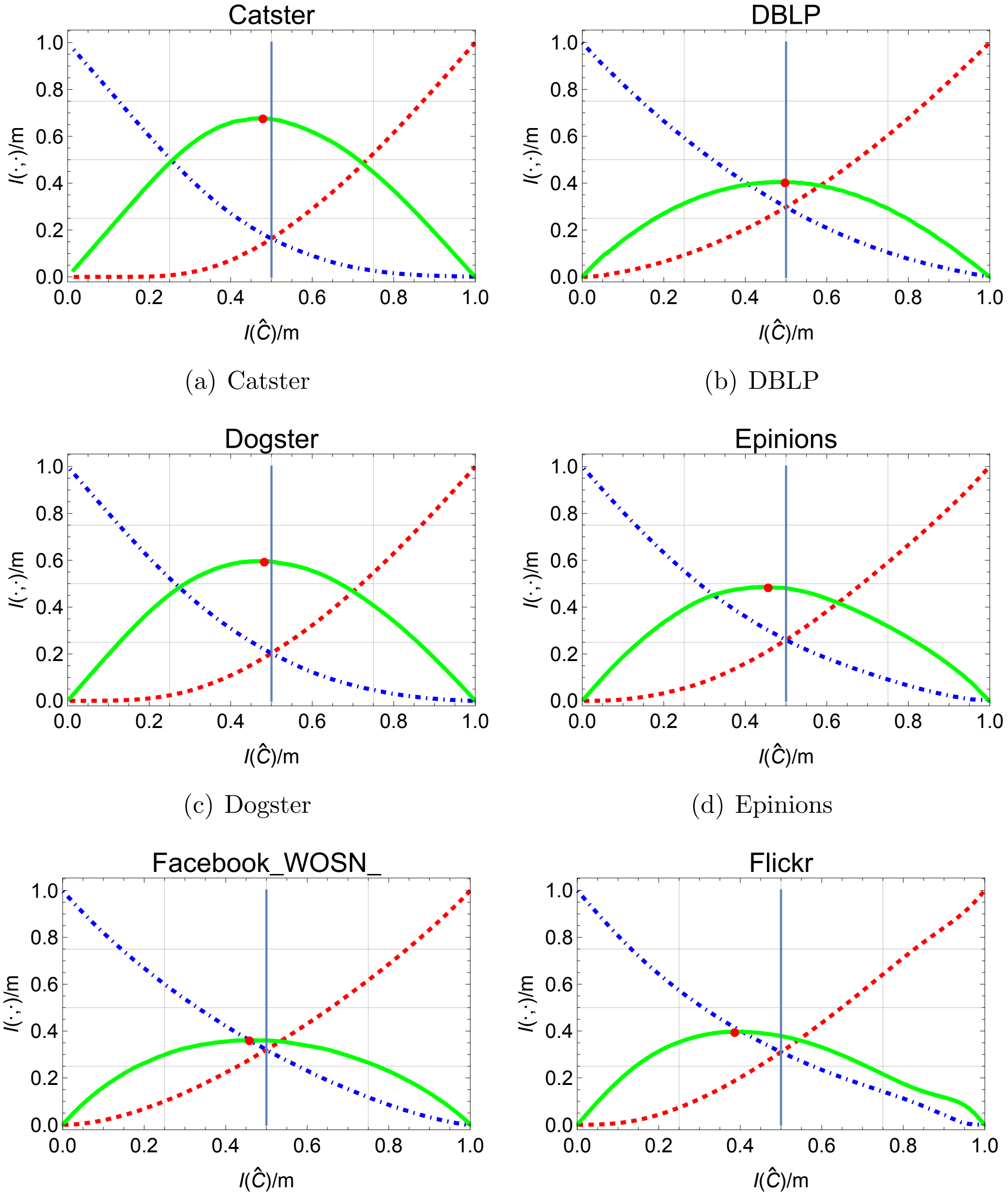

(a) Catster
(b) DBLP
(c) Dogster
(d) Epinions
(e) Facebook
(f) Flickr

Figure 4: Influence shift diagram for Catster, DBLP, Dogster, Epinions, Facebook and Flickr

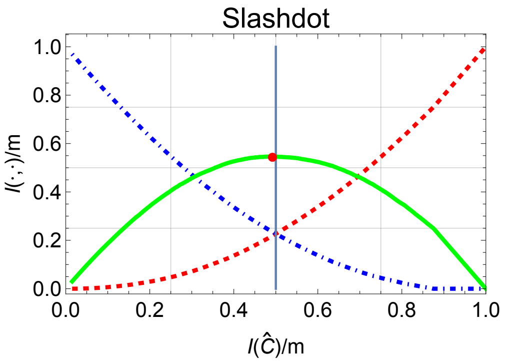

(a) Slashdot

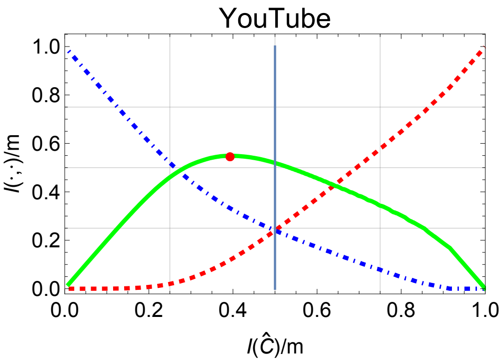

(b) YouTube

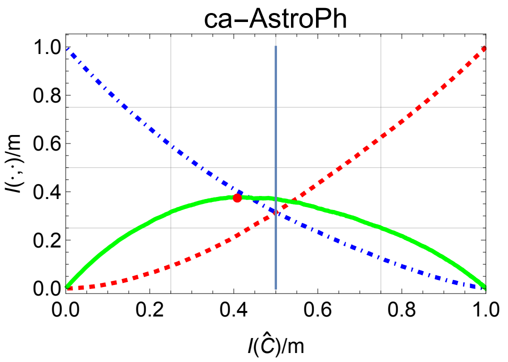

(c) ca-AstroPh

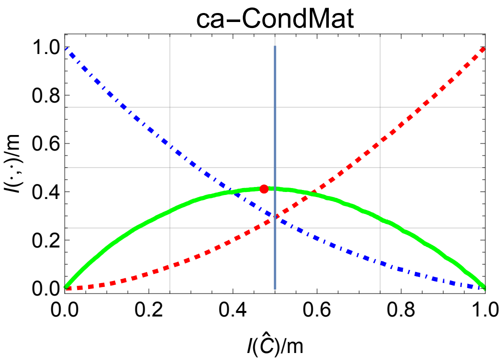

(d) ca-CondMat

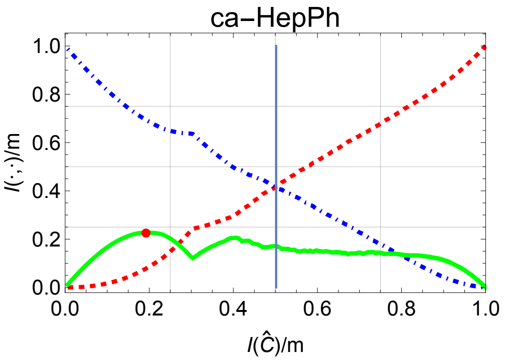

(e) ca-HepPh

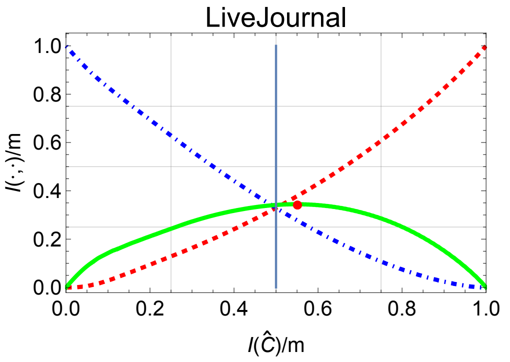

(f) LiveJournal

Figure 5: The elite influence shift diagram for Slashdot, YouTube, ca-AstroPh, ca-CondMat and ca-HepPh

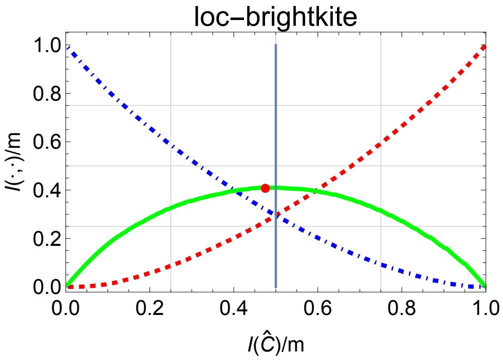

(a) loc-brightkite

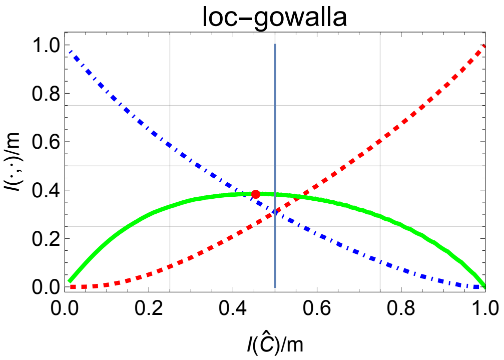

(b) loc-gowalla

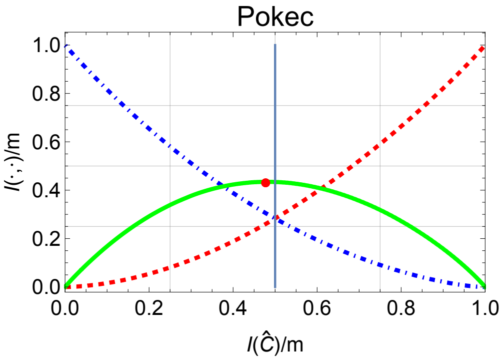

(c) Pokec

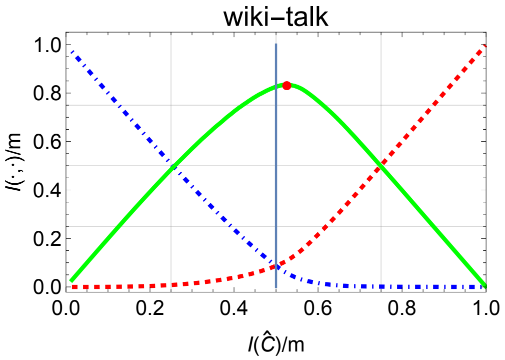

(d) wiki-talk

Figure 6: The elite influence shift diagram for loc-brightkite, loc-gowalla, Pokec and wiki-talk

3.2 $x = \frac{|\hat{C}|}{n}$

The X-axis in figures at this section is on a linerar scale, where a point x (in [0,1]) represents an core of size $\frac{|\hat{C}|}{n}$.

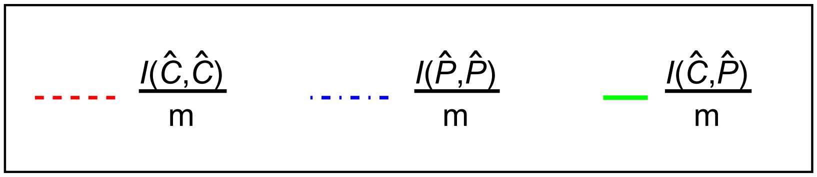

(a)

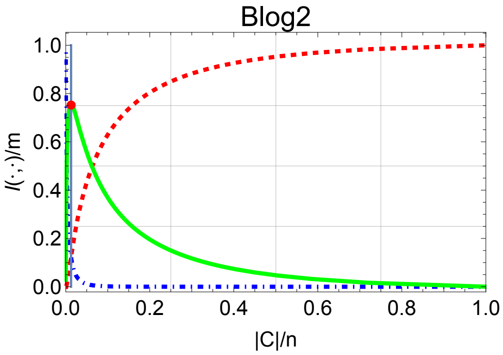

(b) Blog

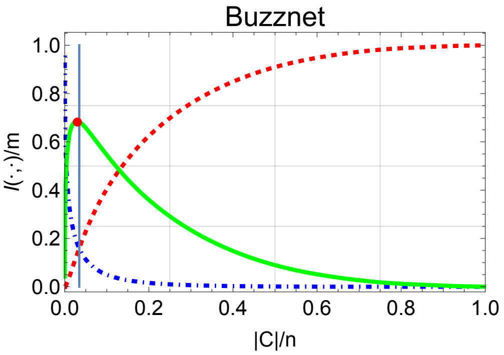

(c) Buzznet

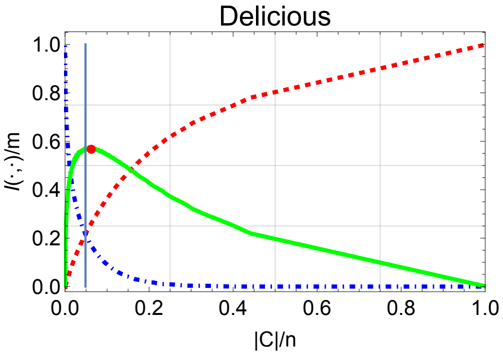

(d) Delicious

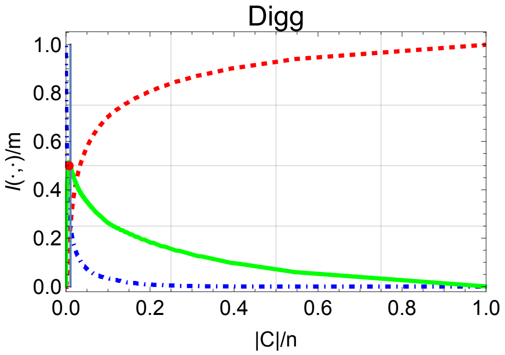

(e) Digg

Figure 7: The influence shift diagram for Blog, Buzznet, Delicious and Digg

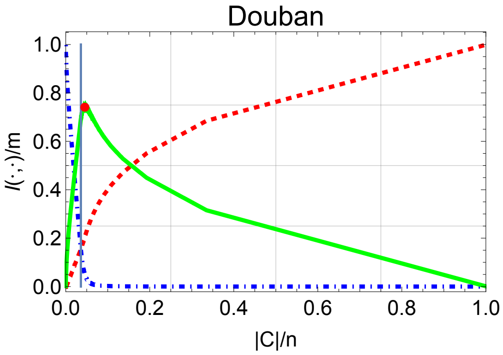

(a) Douban

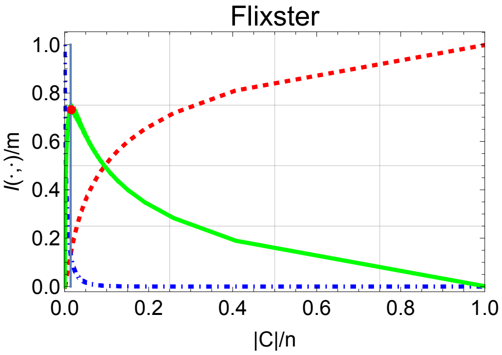

(b) Flixster

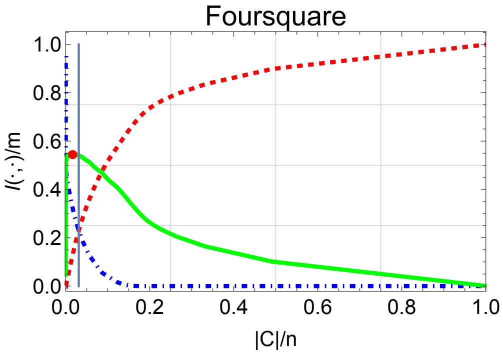

(c) Foursquare

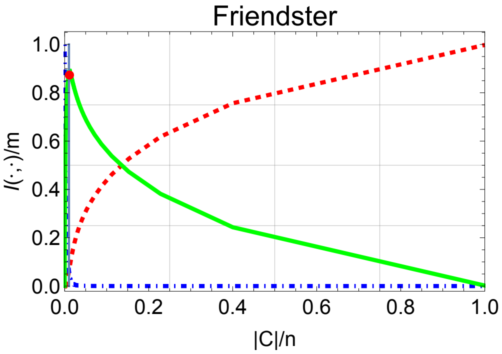

(d) Friendster

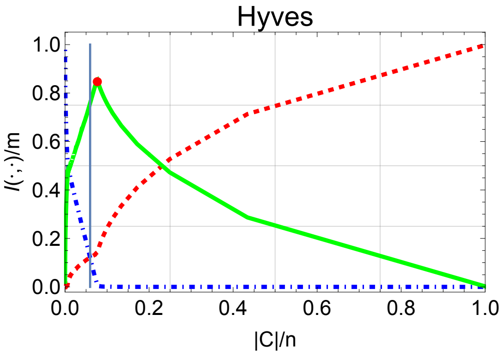

(e) Hyves

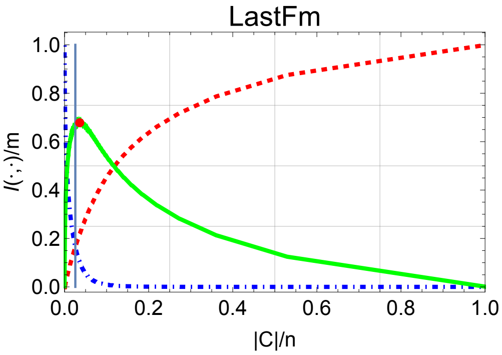

(f) LastFm

Figure 8: Influence shift diagram for Douban and Flixster, Foursquare, Friendster, Hyves and LastFm

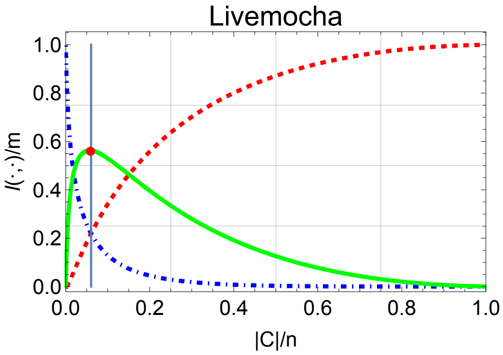

(a) Livemocha

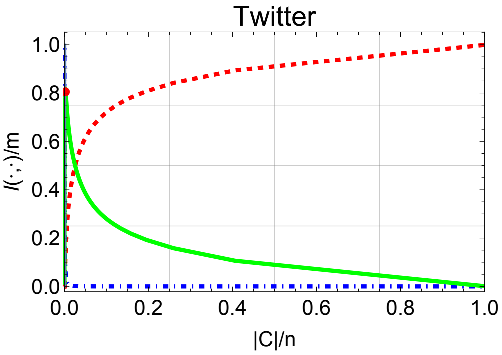

(b) Twitter

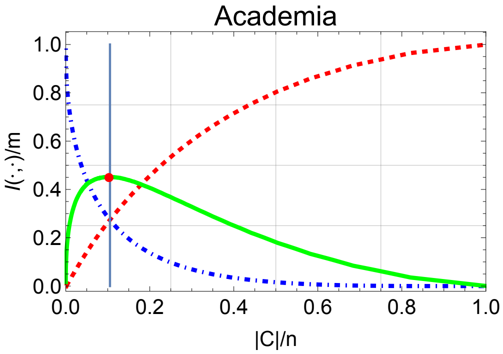

(c) Academia

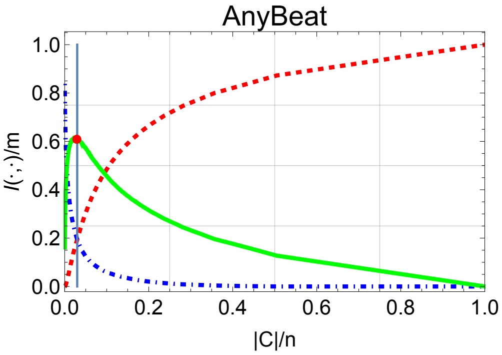

(d) AnyBeat

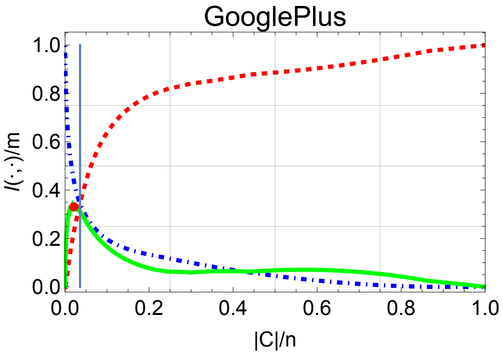

(e) GooglePlus

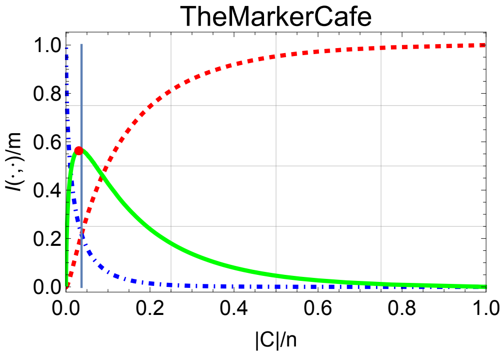

(f) TheMarkerCafe

Figure 9: Influence shift diagram for Livemocha, Twitter, Academia, AnyBeat, GooglePlus and TheMarkerCafe

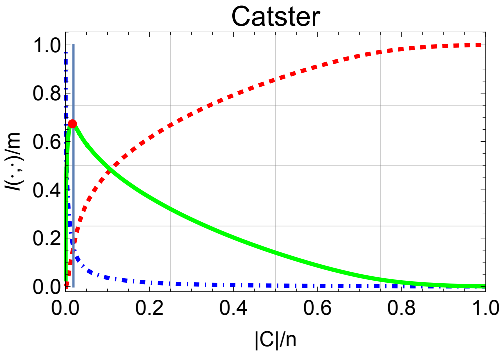

(a) Catster

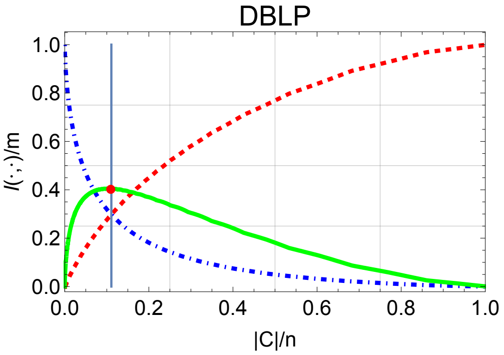

(b) DBLP

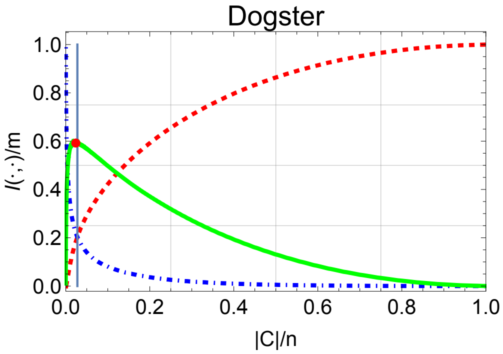

(c) Dogster

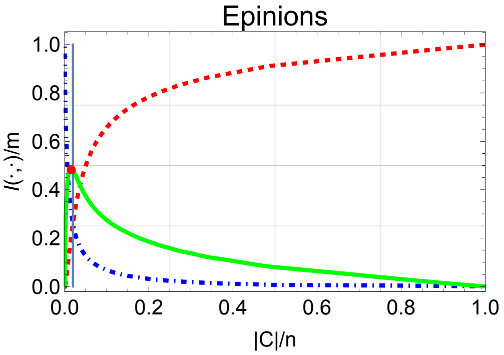

(d) Epinions

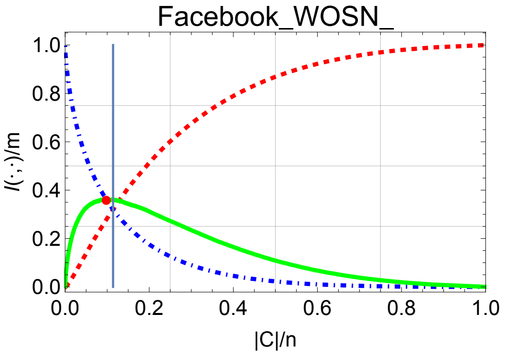

(e) Facebook

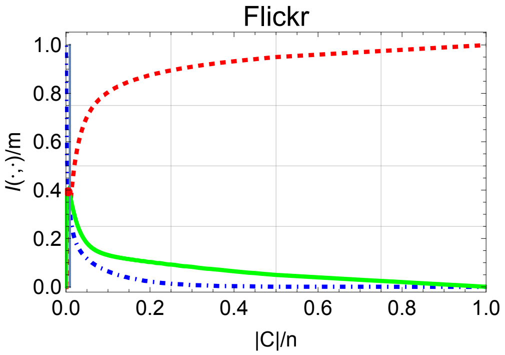

(f) Flickr

Figure 10: Influence shift diagram for Catster, DBLP, Dogster, Epinions, Facebook and Flickr

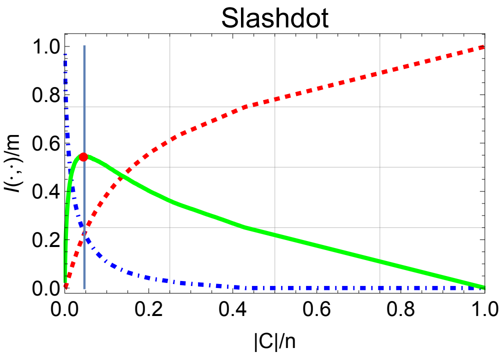

(a) Slashdot

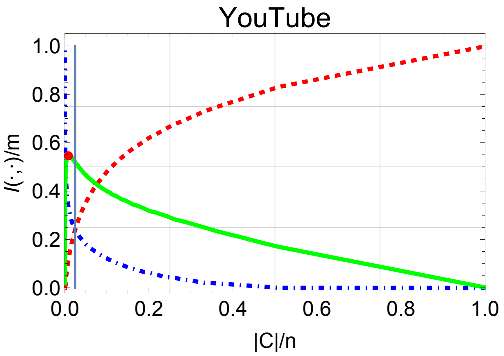

(b) YouTube

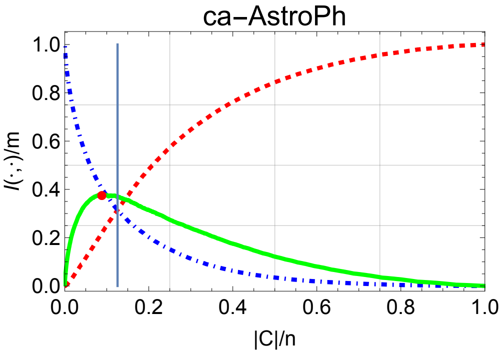

(c) ca-AstroPh

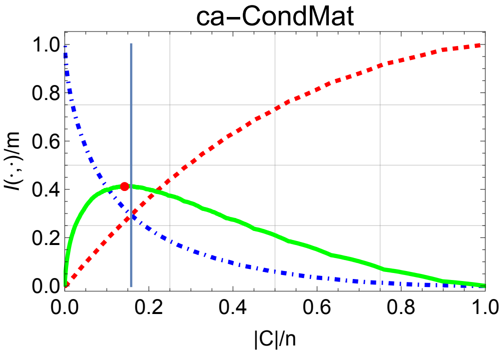

(d) ca-CondMat

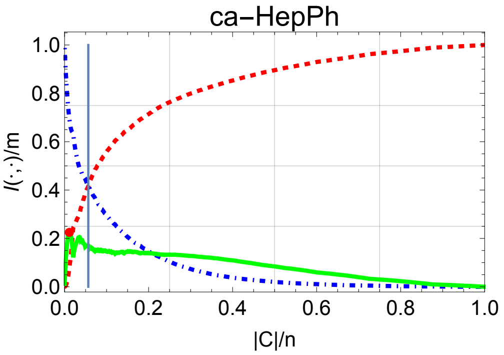

(e) ca-HepPh

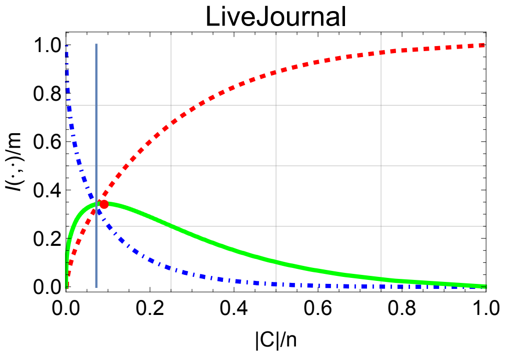

(f) LiveJournal

Figure 11: The elite influence shift diagram for Slashdot, YouTube, ca-AstroPh, ca-CondMat and ca-HepPh

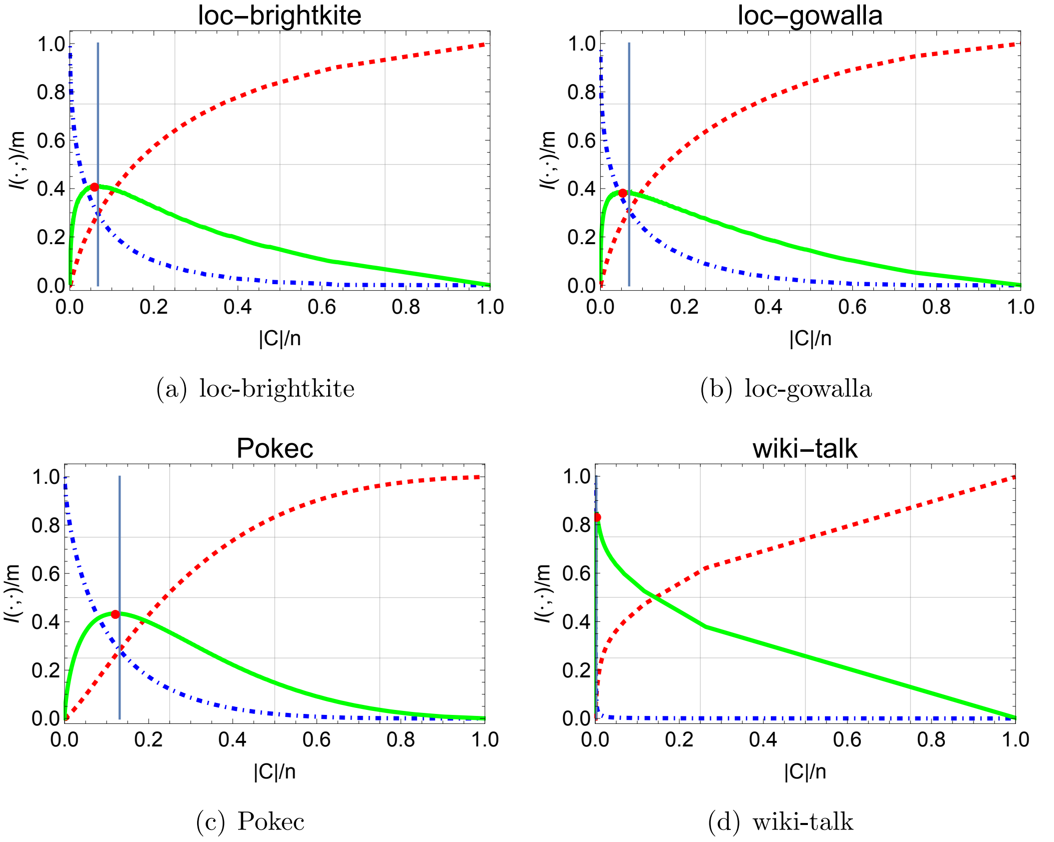

(a) loc-brightkite

(b) loc-gowalla

(c) Pokec

(d) wiki-talk

Figure 12: The elite influence shift diagram for loc-brightkite, loc-gowalla, Pokec and wiki-talk

3.3 $|\hat{C}| = n^x$ and $x = \log|\hat{C}|/\log n$

The X-axis in figures at this section is on a logarithmic scale, where a point x (in [0,1]) represents an core of size n^x.

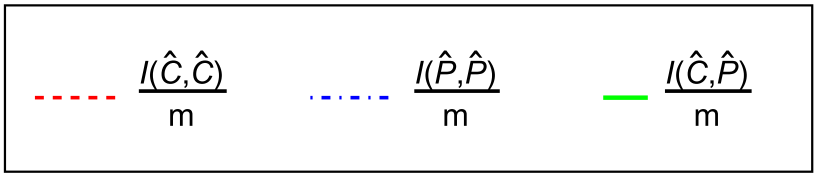

(a)

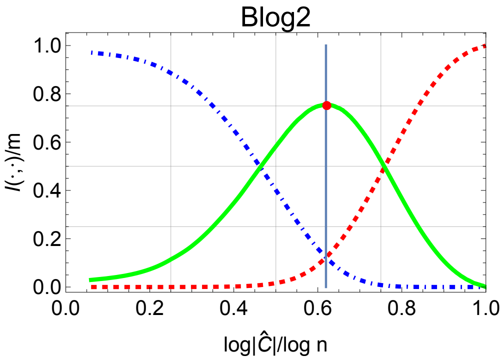

(b) Blog

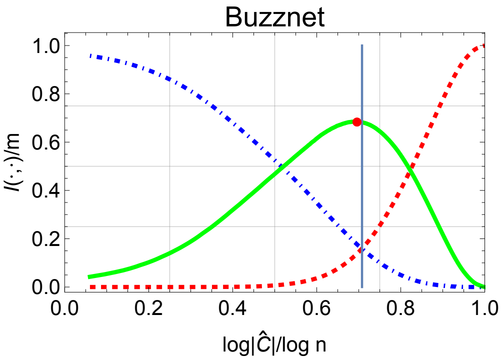

(c) Buzznet

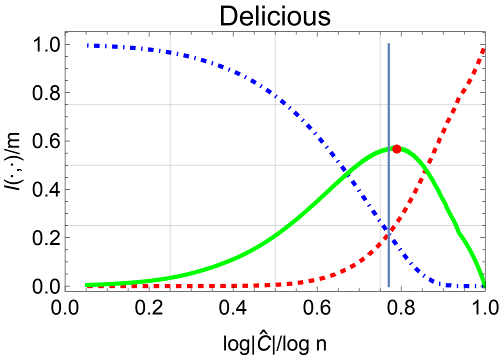

(d) Delicious

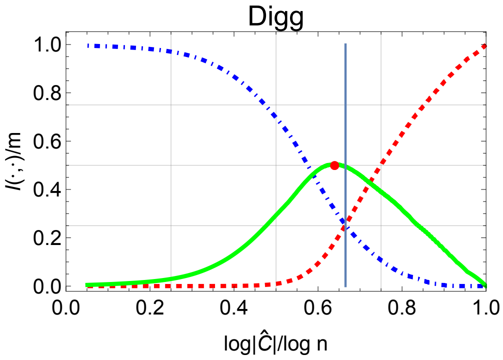

(e) Digg

Figure 13: The influence shift diagram for Blog, Buzznet, Delicious and Digg

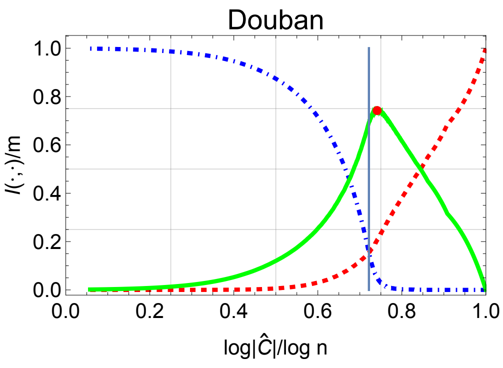

(a) Douban

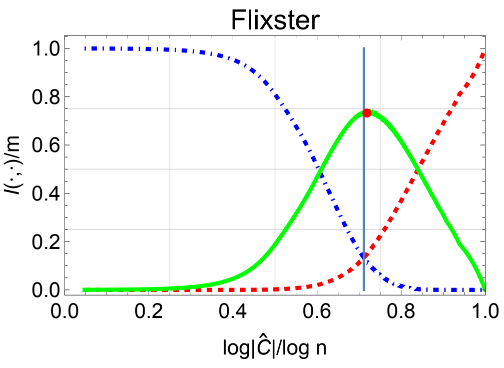

(b) Flixster

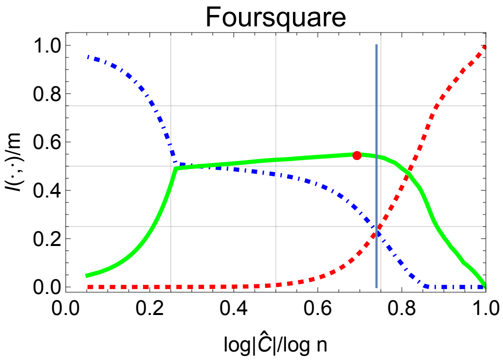

(c) Foursquare

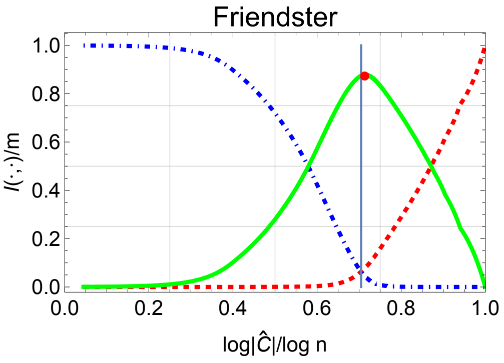

(d) Friendster

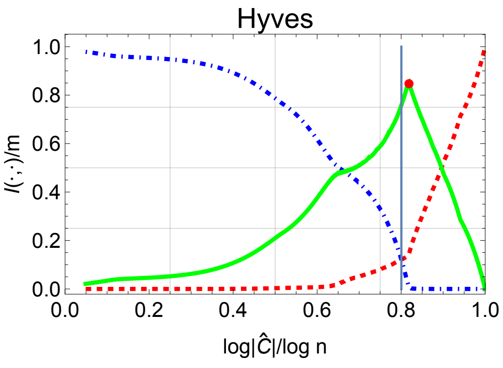

(e) Hyves

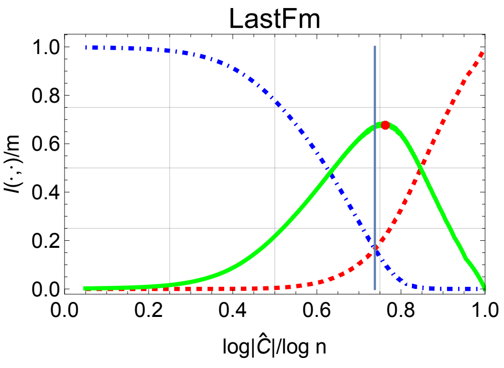

(f) LastFm

Figure 14: Influence shift diagram for Douban and Flixster, Foursquare, Friendster, Hyves and LastFm

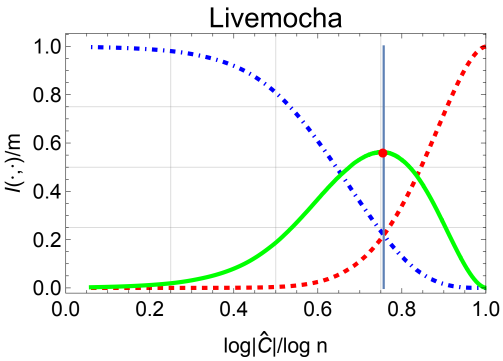

(a) Livemocha

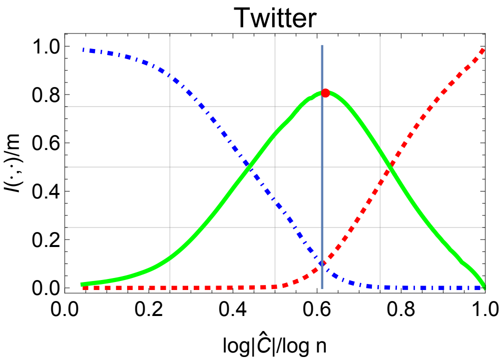

(b) Twitter

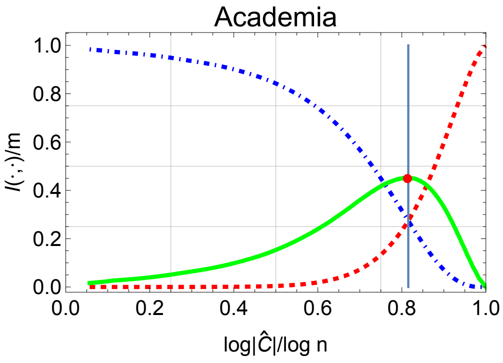

(c) Academia

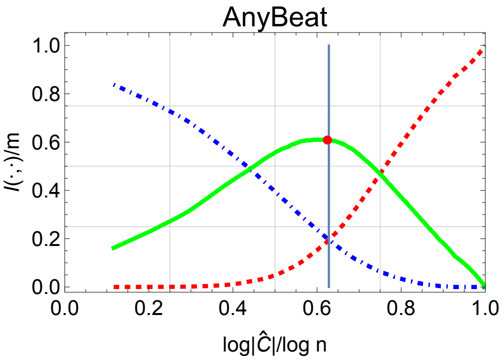

(d) AnyBeat

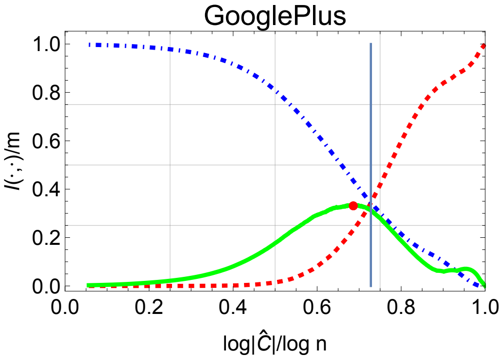

(e) GooglePlus

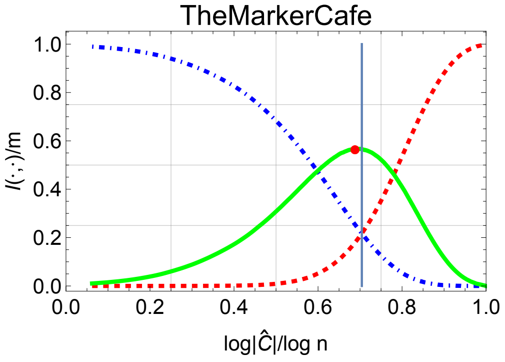

(f) TheMarkerCafe

Figure 15: Influence shift diagram for Livemocha, Twitter, Academia, AnyBeat, GooglePlus and TheMarkerCafe

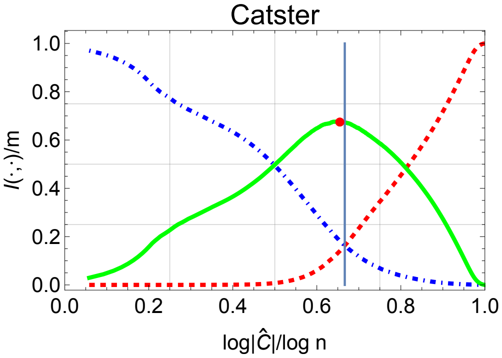

(a) Catster

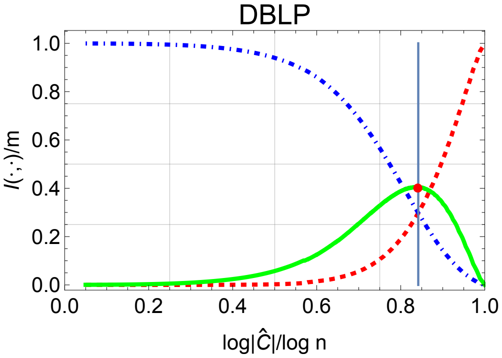

(b) DBLP

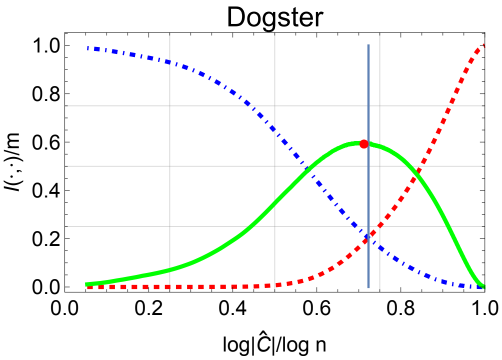

(c) Dogster

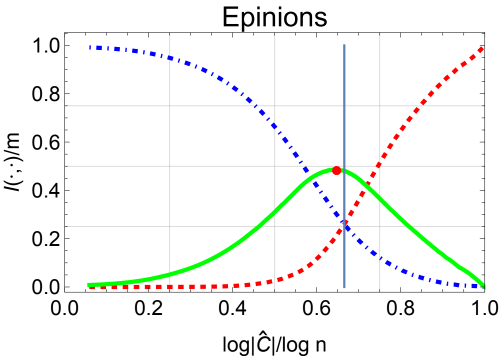

(d) Epinions

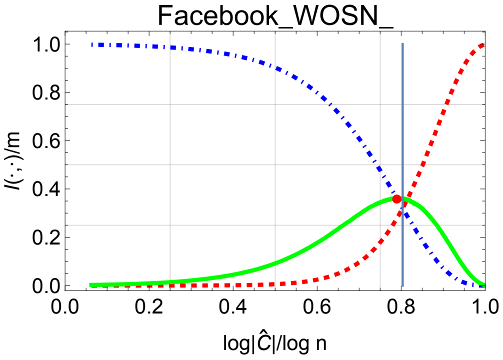

(e) Facebook

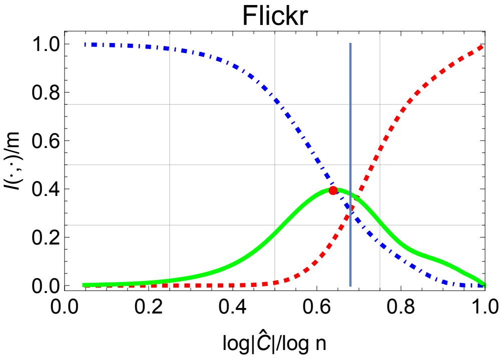

(f) Flickr

Figure 16: Influence shift diagram for Catster, DBLP, Dogster, Epinions, Facebook and Flickr

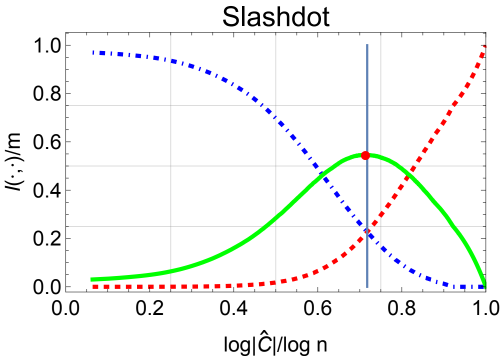

(a) Slashdot

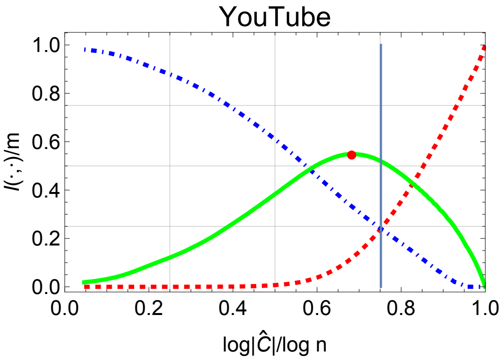

(b) YouTube

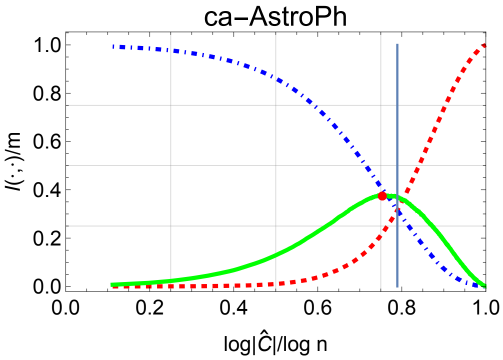

(c) ca-AstroPh

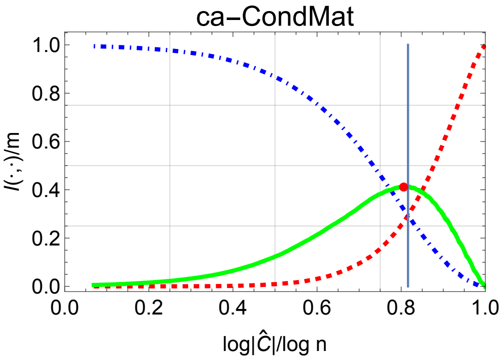

(d) ca-CondMat

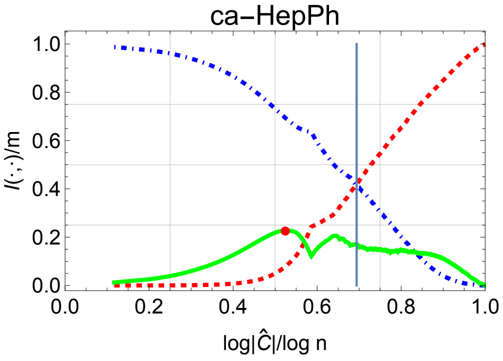

(e) ca-HepPh

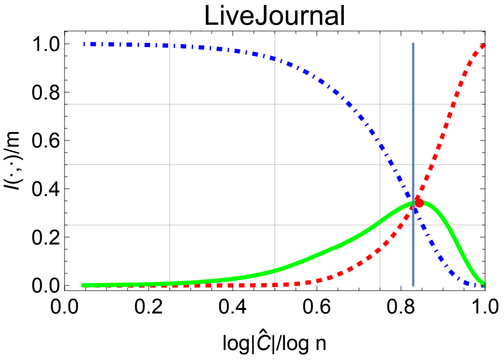

(f) LiveJournal

Figure 17: The elite influence shift diagram for Slashdot, YouTube, ca-AstroPh, ca-CondMat and ca-HepPh

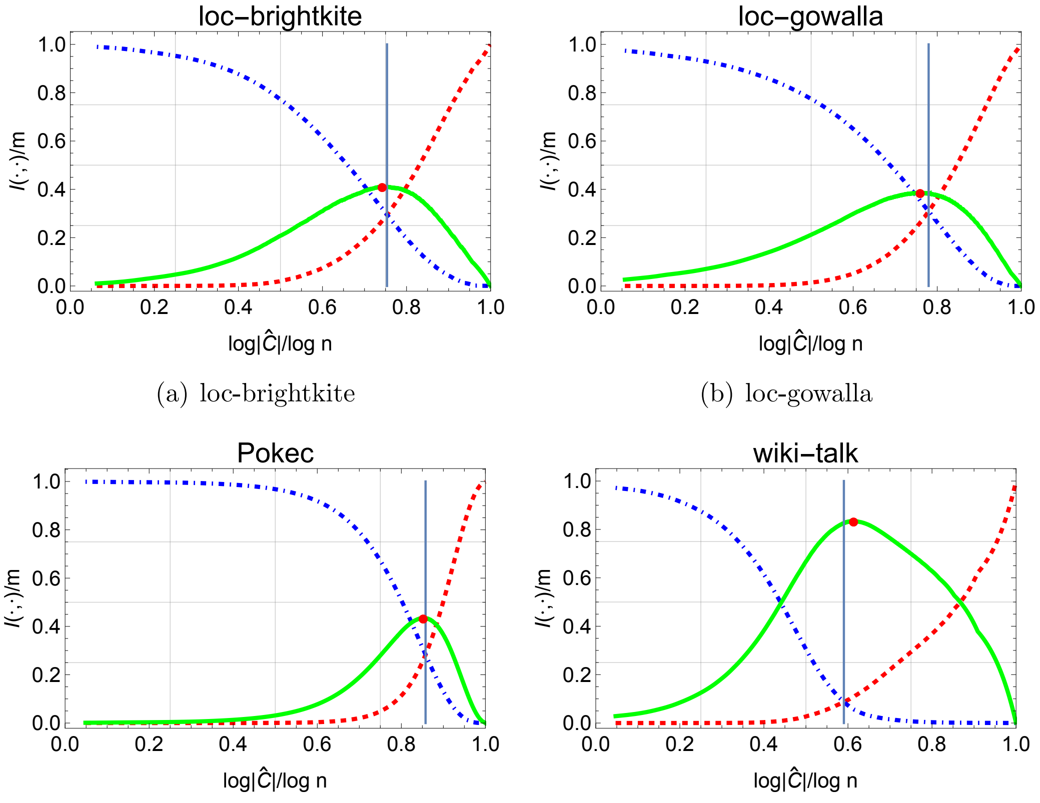

(a) loc-brightkite

(b) loc-gowalla

(c) Pokec

(d) wiki-talk

Figure 18: The elite influence shift diagram for loc-brightkite, loc-gowalla, Pokec and wiki-talk

3.4 Axioms in Growing Networks

In this section we show the values of the observed dominance $\mathrm{dom}(\hat{E}_t)$, robustness $\mathrm{rob}(\hat{E}_t)$ and density $\mathrm{dens}(\hat{E}_t)$ as the networks grow in size over time. We present cores of k-rich-club in 5 networks in the dataset, with available time information. We focus on four possible core sizes: the size k_{sp} at the symmetry point, a sub-linear core of size $n^{0.75}$, and linear size cores containing 1% or 10% of the network vertices. The X Axis represents the growing size of the networks as percentage of the final size and it's on a logarithmic scale.

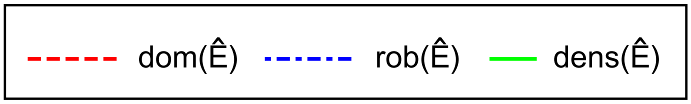

Cores of Size of 1% of the Network Size

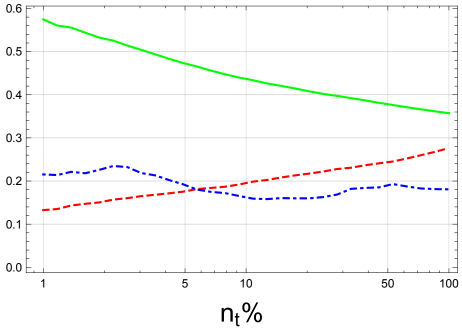

(a) DBLP

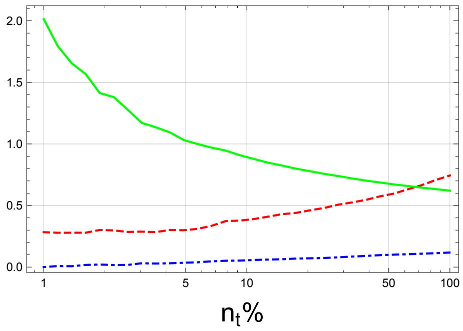

(b) Slashdot

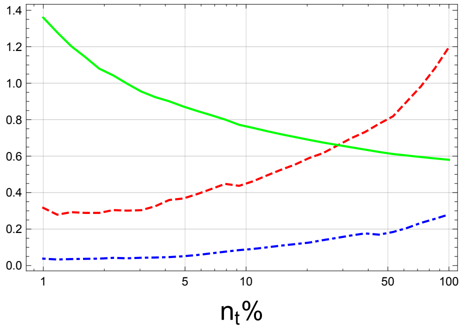

(c) Epinions

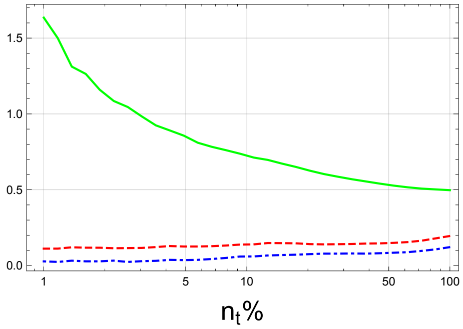

(d) Facebook

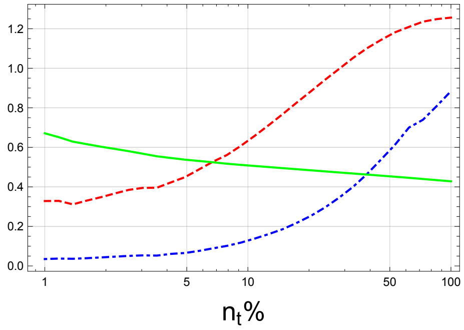

(e) Flickr

Figure 19: Domninace, Robustness and Density Over Time for DBLP, Slashdot, Epinions, Facebook and Flicker

Cores of Size of 10% of the Network Size

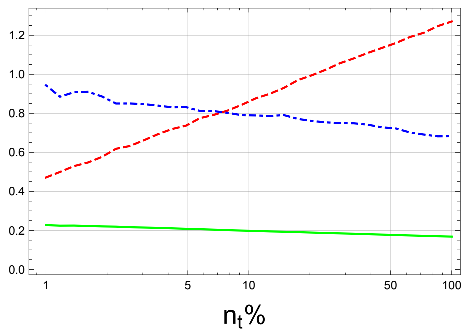

(a) DBLP

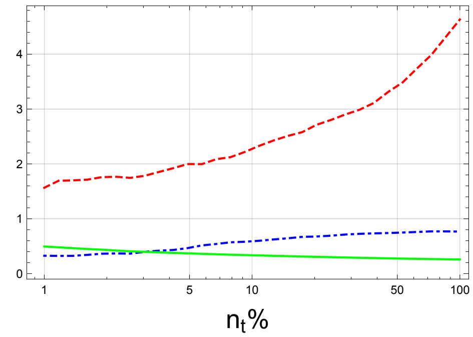

(b) Slashdot

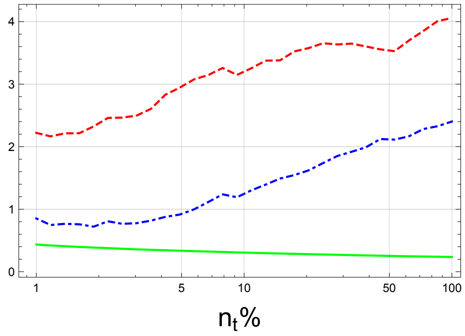

(c) Epinions

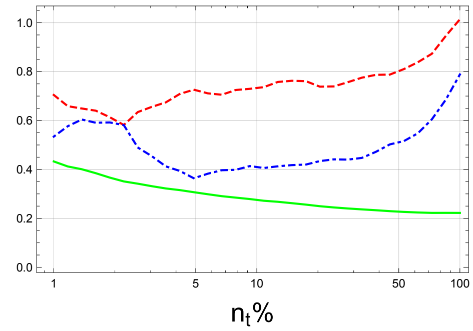

(d) Facebook

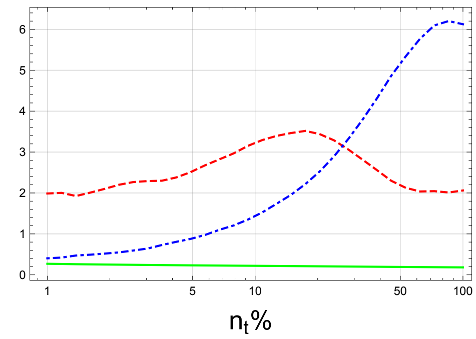

(e) Flickr

Figure 20: Domninace, Robustness and Density Over Time for DBLP, Slashdot, Epinions, Facebook and Flickr

Cores of Size of $n^{0.75}$

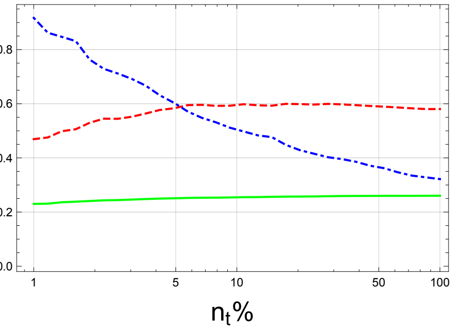

(a) DBLP

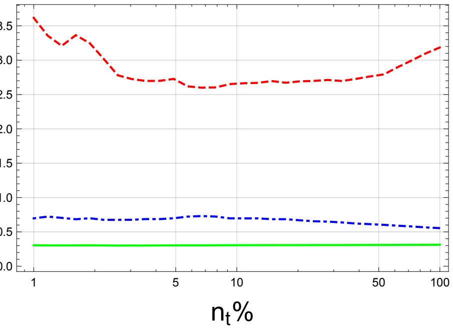

(b) Slashdot

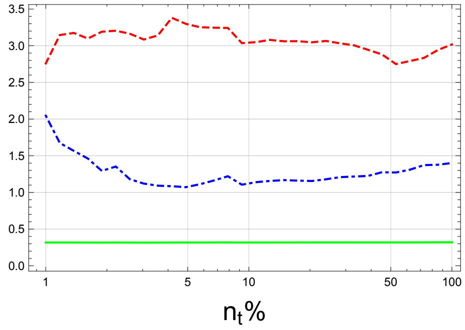

(c) Epinions

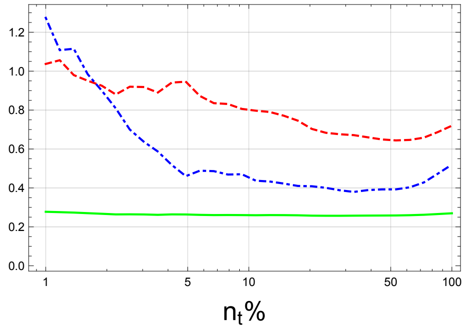

(d) Facebook

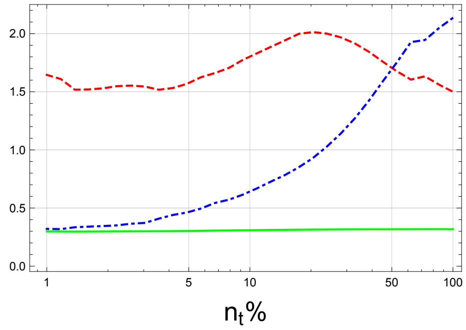

(e) Flickr

Figure 21: Domninace, Robustness and Density Over Time for Slashdo, Epinions , Facebook and Flickr

Cores at the Symmetry Point

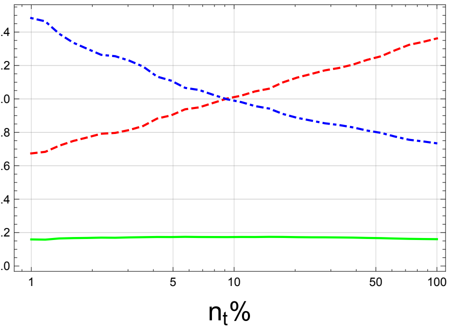

(a) DBLP

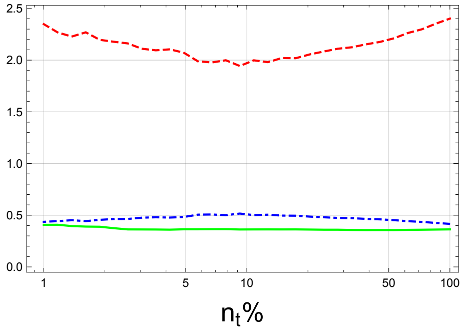

(b) Slashdot

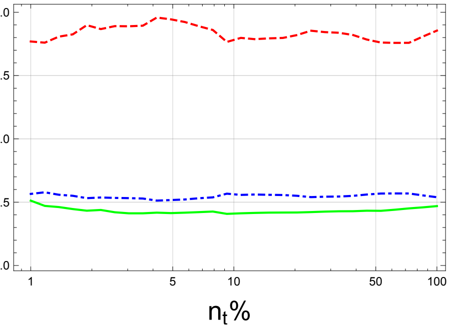

(c) Epinions

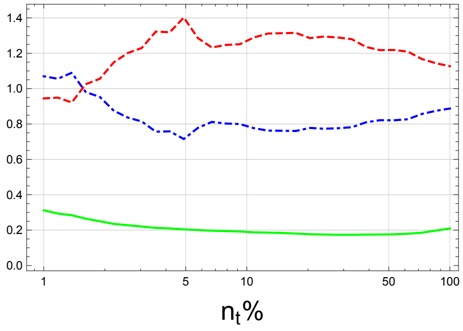

(d) Facebook

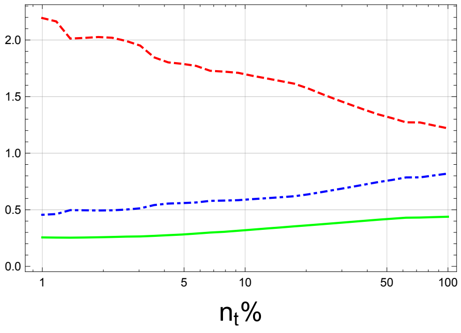

(e) Flickr

Figure 22: Domninace, Robustness and Density Over Time for Slashdot, Epinions , Facebook and Flicker

4 Conferences Networks

Conference	n	m	Duration (years)
CASES	1095	4425	20
EC	1128	4878	20
Infocom	8783	59221	20
IPSN	1753	7809	20
KDD	4584	29379	20
MOBIHOC	1381	6614	19
PODC	1847	9030	19
SOCG	1468	8727	19
SODA	2846	21584	19
SPAA	1738	8682	20
STOC	2252	17506	20
UIST	2055	9912	20
WWW	4297	24358	20

Table 4: List of All Tested Conferences

	Rich Club				
Conference	$\lvert\hat{E}_{sp}\rvert$	x ($\lvert\hat{E}_{sp}\rvert = n^x$)	$I(\hat{E}, \hat{E})$	$I(\hat{P}, \hat{P})$	$I(\hat{E}, \hat{P})$
CASES	235	0.78	1284	1277	1864
EC	190	0.75	1439	1437	2002
Infocom	1267	0.79	16494	16471	268543
IPSN	347	0.78	2439	2433	2937
KDD	736	0.78	8888	8876	11615
MOBIHOC	276	0.78	1867	1859	2888
PODC	312	0.76	2619	2605	3806
SOCG	184	0.72	2566	2557	3604
SODA	466	0.77	6070	6060	9454
SPAA	321	0.77	2544	2531	3607
STOC	384	0.77	4862	4836	7808
UIST	349	0.77	2931	2923	4058
WWW	753	0.79	7315	7301	9742

Table 5: Conferences' Symmetry Point

	Rich Club		
Network	dom($\hat{E}$)	rob($\hat{E}$)	dens($\hat{E}$)
CASES	0.12	0.05	1.59
EC	0.14	0.04	1.67
Infocom	0.18	0.07	1.05
IPSN	0.16	0.05	1.43
KDD	0.17	0.06	1.20
MOBIHOC	0.12	0.04	1.51
PODC	0.15	0.05	1.45
SOCG	0.20	0.05	1.71
SODA	0.13	0.04	1.37
SPAA	0.13	0.03	1.44
STOC	0.13	0.04	1.45
UIST	0.16	0.06	1.39
WWW	0.14	0.05	1.15

Table 6: Dominance, Robustness and Density at Cores of Size $0.01n$

	Rich Club		
Network	dom($\hat{E}$)	rob($\hat{E}$)	dens($\hat{E}$)
CASES	0.75	0.33	0.64
EC	0.89	0.44	0.67
Infocom	1.16	0.44	0.55
IPSN	0.75	0.42	0.61
KDD	0.92	0.47	0.59
MOBIHOC	0.85	0.31	0.65
PODC	0.94	0.40	0.64
SOCG	1.17	0.58	0.73
SODA	0.99	0.38	0.67
SPAA	0.86	0.38	0.64
STOC	0.98	0.35	0.70
UIST	0.99	0.41	0.63
WWW	0.88	0.41	0.57

Table 7: Dominance, Robustness and Density at Cores of Size $0.1n$

	Rich Club		
Network	dom($\hat{E}$)	rob($\hat{E}$)	dens($\hat{E}$)
CASES	1.20	0.55	0.52
EC	1.43	0.73	0.55
Infocom	1.18	0.46	0.55
IPSN	1.02	0.64	0.52
KDD	1.04	0.59	0.55
MOBIHOC	1.31	0.51	0.55
PODC	1.36	0.60	0.55
SOCG	1.77	0.88	0.61
SODA	1.34	0.51	0.61
SPAA	1.23	0.58	0.55
STOC	1.38	0.51	0.62
UIST	1.28	0.61	0.54
WWW	1.03	0.52	0.53

Table 8: Dominance, Robustness and Density at Cores of Size $n^{0.75}$

	Rich Club		
Network	dom($\hat{E}$)	rob($\hat{E}$)	dens($\hat{E}$)
CASES	1.46	0.69	0.47
EC	1.39	0.72	0.55
Infocom	1.59	0.63	0.49
IPSN	1.21	0.83	0.47
KDD	1.31	0.77	0.50
MOBIHOC	1.55	0.65	0.51
PODC	1.46	0.69	0.53
SOCG	1.41	0.71	0.67
SODA	1.56	0.64	0.57
SPAA	1.43	0.71	0.51
STOC	1.61	0.62	0.59
UIST	1.39	0.72	0.51
WWW	1.33	0.75	0.47

Table 9: Dominance, Robustness and Density at Cores at the Symmetry Point

4.1 Axioms in Growing Networks

In this section we show the values of the observed dominance dom($\hat{E}_k$), robustness rob($\hat{E}_k$) and density dens($\hat{E}_k$) as the networks grow in size over time. We present cores of k-rich-club in 13 conferences networks, for cores at the size k_{sp} at the symmetry point. The X Axis represents the growing size of the networks as percentage of the final size and it's on a logarithmic scale.

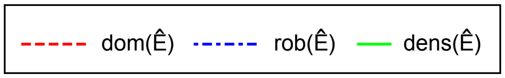

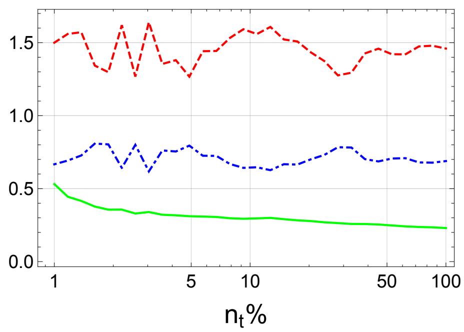

(a) CASES

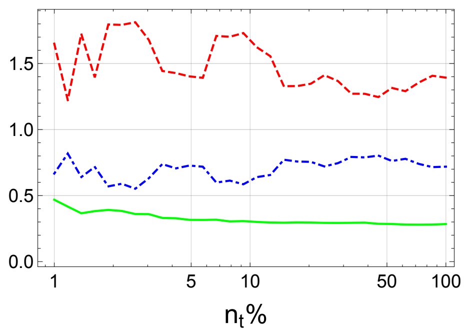

(b) EC

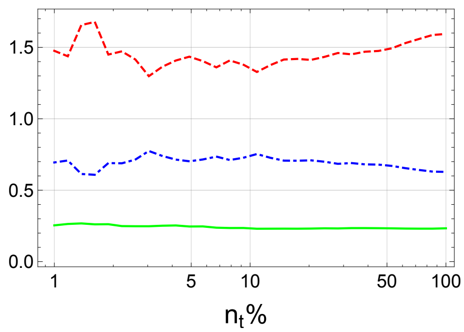

(c) Infocom

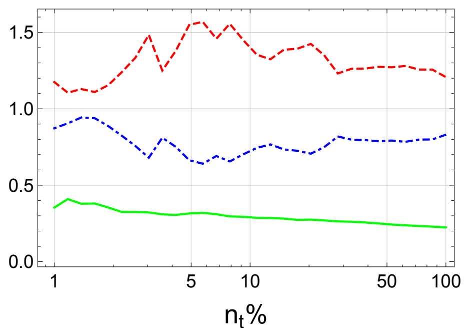

(d) IPSN

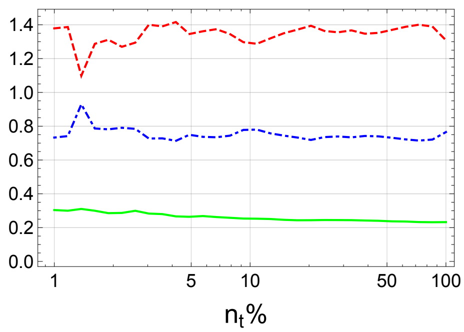

(e) KDD

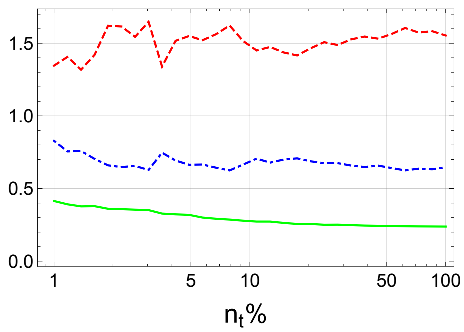

(f) MOBIHOC

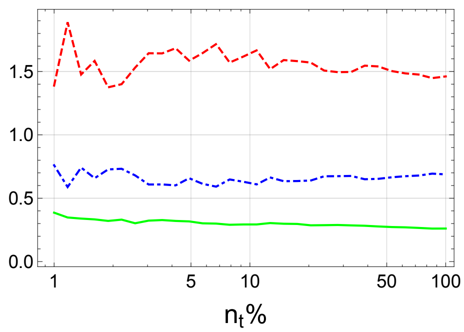

(g) PODC

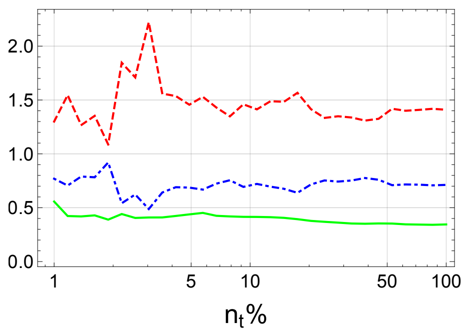

(h) SOCG

Figure 23: Domninace, Robustness and Density Over Time for CASES, EC, Infocom, IPSN, KDD, MOBIHOC, PODC and SOCG

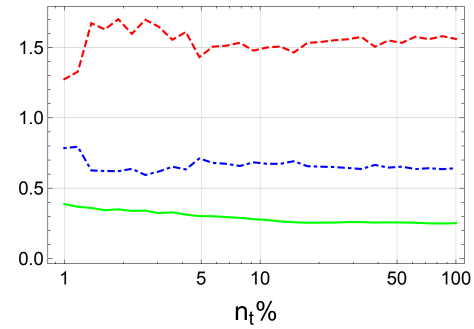

(a) SODA

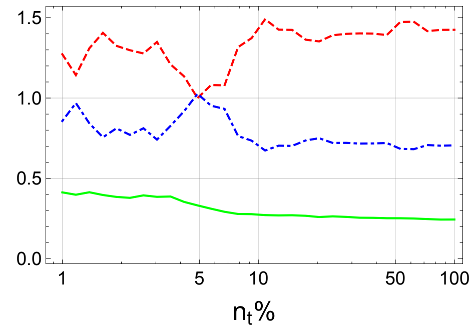

(b) SPAA

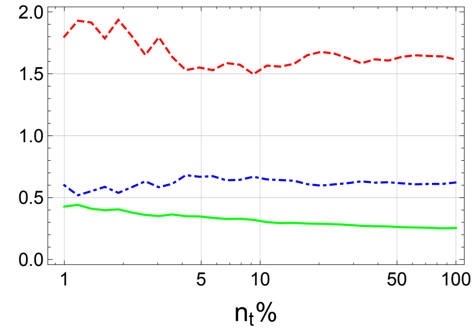

(c) STOC

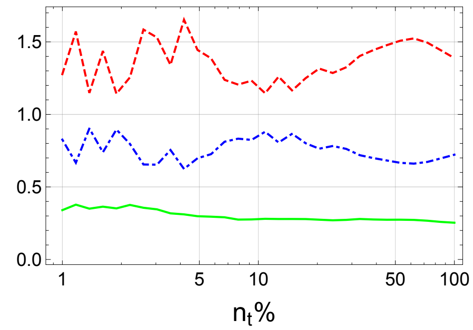

(d) UIST

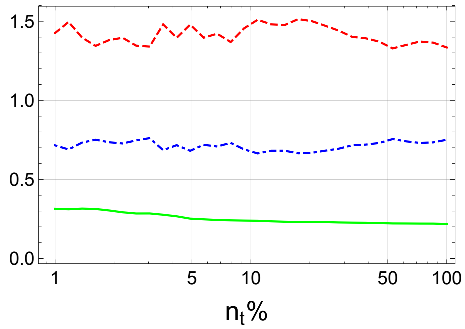

(e) WWW

Figure 24: Domninace, Robustness and Density Over Time for SODA, SPAA, STOC, UIST and WWW

References

[1] Academia. http://proj.ise.bgu.ac.il/sns/academia.html.

[2] Anybeat. http://proj.ise.bgu.ac.il/sns/anybeat.html.

[3] Blog2. http://socialcomputing.asu.edu/datasets/BlogCatalog2.

[4] Buzznet. http://socialcomputing.asu.edu/datasets/Buzznet.

[5] ca-astroph. http://snap.stanford.edu/data/ca-AstroPh.html.

[6] ca-condmat. http://snap.stanford.edu/data/ca-CondMat.html.

[7] ca-hepph. http://snap.stanford.edu/data/ca-HepPh.html.

[8] Catster-social. http://konect.uni-koblenz.de/networks/petster-friendships-cat.

[9] Dblp-contact. http://konect.uni-koblenz.de/networks/dblp-coauthor.

[10] Delicious. http://socialcomputing.asu.edu/datasets/Delicious.

[11] Digg. http://socialcomputing.asu.edu/datasets/Digg.

[12] Dogster-social. http://konect.uni-koblenz.de/networks/petster-friendships-dog.

[13] Douban. http://socialcomputing.asu.edu/datasets/Douban.

[14] Epinions-social. http://konect.uni-koblenz.de/networks/epinions.

[15] Facebook-wosn–social. http://konect.uni-koblenz.de/networks/facebook-wosn-links.

[16] Flickr-social. http://konect.uni-koblenz.de/networks/flickr-growth.

[17] Flixster. http://socialcomputing.asu.edu/datasets/Flixster.

[18] Foursquare. http://socialcomputing.asu.edu/datasets/Foursquare.

[19] Friendster. http://socialcomputing.asu.edu/datasets/Friendster.

[20] Googleplus. http://proj.ise.bgu.ac.il/sns/googlep.html.

[21] Hyves. http://socialcomputing.asu.edu/datasets/Hyves.

[22] Lastfm. http://socialcomputing.asu.edu/datasets/Last.fm.

[23] Livejournal. http://snap.stanford.edu/data/soc-LiveJournal1.html.

[24] Livemocha. http://socialcomputing.asu.edu/datasets/Livemocha.

[25] loc-brightkite. http://snap.stanford.edu/data/loc-brightkite.html.

[26] loc-gowalla. http://snap.stanford.edu/data/loc-gowalla.html.

[27] Pokec. http://snap.stanford.edu/data/soc-pokec.html.

[28] Slashdot-communication. http://konect.uni-koblenz.de/networks/slashdot-threads.

[29] Themarkercafe. http://proj.ise.bgu.ac.il/sns/themarker.html.

[30] Twitter. http://socialcomputing.asu.edu/datasets/Twitter.

[31] wiki-talk. http://snap.stanford.edu/data/wiki-Talk.html.

[32] Youtube-social. http://konect.uni-koblenz.de/networks/youtube-u-growth.

[33] Statistical analysis of the social network and discussion threads in Slashdot. In *Proc. Int. World Wide Web Conf.* (2008), pp. 645–654.

[34] Backstrom, L., Huttenlocher, D., Kleinberg, J., and Lan, X. Group formation in large social networks: membership, growth, and evolution. In *Proc. 12th ACM SIGKDD Int. Conf. on Knowledge discovery and data mining* (2006), pp. 44–54.

[35] Cho, E., Myers, S. A., and Leskovec, J. Friendship and mobility: user movement in location-based social networks. In *Proc. 17th ACM SIGKDD Int. Conf. on Knowledge discovery and data mining* (2011), ACM, pp. 1082–1090.

[36] Fire, M., Tenenboim, L., Lesser, O., Puzis, R., Rokach, L., and Elovici, Y. Link prediction in social networks using computationally efficient topological features. In *Privacy, Security, Risk and Trust (PASSAT), 2011 IEEE Third International Conference on and 2011 IEEE Third International Confernece on Social Computing (SocialCom)* (2011), IEEE, pp. 73–80.

[37] Fire, M., Tenenboim, L., Puzis, R., Lesser, O., Rokach, L., and Elovici, Y. Computationally efficient link prediction in variety of social networks.

[38] Kunegis, J. Konect - the koblenz network collection. pp. 1343–1350.

[39] Leskovec, J., Huttenlocher, D., and Kleinberg, J. Predicting positive and negative links in online social networks. In *Proc. 19th Int. Conf. on World wide web* (2010), ACM, pp. 641–650.

[40] Leskovec, J., Huttenlocher, D., and Kleinberg, J. Signed networks in social media. In *Proc. SIGCHI Conf. on Human Factors in Computing Systems* (2010), ACM, pp. 1361–1370.

[41] Leskovec, J., Kleinberg, J., and Faloutsos, C. Graph evolution: Densification and shrinking diameters. *ACM Trans. Knowl. Discov. Data 1*, 1 (Mar. 2007).

[42] Leskovec, J., Lang, K. J., Dasgupta, A., and Mahoney, M. W. Community structure in large networks: Natural cluster sizes and the absence of large well-defined clusters. *CoRR abs/0810.1355* (2008).

[43] Ley, M. The DBLP computer science bibliography: Evolution, research issues, perspectives. In *Proc. Int. Symposium on String Processing and Information Retrieval* (2002), pp. 1–10.

[44] M., F., R., P., and Y., E. Link prediction in highly fractional data sets. *Handbook of Computational Approaches to Counterterrorism* (2012).

[45] Massa, P., and Avesani, P. Controversial users demand local trust metrics: an experimental study on epinions.com community. In *Proc. American Association for Artificial Intelligence Conf.* (2005), pp. 121–126.

[46] Mislove, A. *Online Social Networks: Measurement, Analysis, and Applications to Distributed Information Systems.* PhD thesis, Rice University, 2009.

[47] Mislove, A., Koppula, H. S., Gummadi, K. P., Druschel, P., and Bhattacharjee, B. Growth of the Flickr social network. In *Proc. Workshop on Online Social Networks* (2008), pp. 25–30.

[48] Takac, L., and Zabovsky, M. Data analysis in public social networks. In *International Scientific Conference and International Workshop Present Day Trends of Innovations* (2012).

[49] Viswanath, B., Mislove, A., Cha, M., and Gummadi, K. P. On the evolution of user interaction in Facebook. In *Proc. Workshop on Online Social Networks* (2009), pp. 37–42.

[50] Zafarani, R., and Liu, H. Social computing data repository at ASU, 2009.
